# Measuring the intangible resources caregivers need to provide nurturing care during the complementary feeding period: a scoping review in low- and lower-middle-income countries

**DOI:** 10.1017/S1368980024000065

**Published:** 2024-01-15

**Authors:** Stephanie L Martin, Amanda A Zongrone, Hope C Craig, Kate Litvin, Peyton Fort, Stephanie Cooper, Mia Haller, Katherine L Dickin

**Affiliations:** 1 Department of Nutrition, Gillings School of Global Public Health, University of North Carolina at Chapel Hill, Chapel Hill, NC, USA; 2 Carolina Population Center, University of North Carolina at Chapel Hill, CB 7461, Chapel Hill, NC, 27599-7461, USA; 3 Independent Consultant, Washington, DC, USA; 4 Division of Nutritional Sciences, Cornell University, Ithaca, New York, USA; 5 USAID Advancing Nutrition, Arlington, Virginia, USA; 6 Global Studies, University of North Carolina at Chapel Hill, Chapel Hill, NC, USA; 7 Department of Health Behavior, Gillings School of Global Public Health, University of North Carolina at Chapel Hill, Chapel Hill, NC, USA; 8 Department of Public and Ecosystem Health, Cornell University, Ithaca, New York, USA

**Keywords:** nutrition, maternal capabilities, maternal capacities, family caregivers, resources for care, social and behavior change, gender roles

## Abstract

**Objective:**

Caregivers require tangible (e.g. food and financial) and intangible resources to provide care to ensure child health, nutrition and development. Intangible resources include beliefs and knowledge, education, self-efficacy, perceived physical health, mental health, healthy stress levels, social support, empowerment, equitable gender attitudes, safety and security and time sufficiency. These intangible caregiver resources are included as intermediate outcomes in nutrition conceptual frameworks yet are rarely measured as part of maternal and child nutrition research or evaluations. To facilitate their measurement, this scoping review focused on understudied caregiver resources that have been measured during the complementary feeding period in low- and lower-middle-income countries.

**Design:**

We screened 9,232 abstracts, reviewed 277 full-text articles and included 163 articles that measured caregiver resources related to complementary feeding or the nutritional status of children 6 months to 2 years of age.

**Results:**

We identified measures of each caregiver resource, though the number of measures and quality of descriptions varied widely. Most articles (77 %) measured only one caregiver resource, mental health (*n* 83) and social support (*n* 54) most frequently. Psychometric properties were often reported for mental health measures, but less commonly for other constructs. Few studies reported adapting measures for specific contexts. Existing measures for mental health, equitable gender attitudes, safety and security and time sufficiency were commonly used; other constructs lacked standardised measures.

**Conclusions:**

Measurement of caregiver resources during the complementary feeding period is limited. Measuring caregiver resources is essential for prioritising caregivers and understanding how resources influence child care, feeding and nutrition.

Adequate maternal and child care practices were first included as an underlying determinant of child survival, growth and development in UNICEF’s 1991 framework for malnutrition^(1)^. In the 1990s, Engle, Menon and Haddad expanded the UNICEF framework and defined three categories of resources caregivers need to provide adequate care for a child: food/economic resources, health resources and ‘resources for care’^(2,3)^. While tangible resources are clearly necessary for improved nutrition, they are not sufficient in the absence of key intangible resources. Resources for care reflect the intangible resources caregivers need and include caregiver education, knowledge and beliefs, self-confidence, physical health and nutritional status, mental health and lack of stress, control of resources and autonomy, social support and time availability and workload^(2,3)^. The critical importance of resources for care as an underlying determinant of child health, nutrition and development has since been recognised in several seminal child nutrition and health frameworks^(4–7)^. Further, many observational and intervention studies have highlighted the critical role that caregivers play in young children’s growth and nutrition^(8)^.

Drawing from Engle *et al.* ^(3)^ and the frameworks described above, we use the term *caregiver resources* as a broad label for the range of intangible resources caregivers need to enact recommended nutrition and caregiving practices to provide nurturing care. We further refined Engle *et al.*’s original list of caregiver resources based on subsequent related conceptual work and empirical evidence related to resources for care^(9)^, maternal capabilities^(10)^ and maternal capacities^(11–13)^. We added safety and security^(6)^ and equitable gender attitudes^(10,14)^. We use the more comprehensive term *empowerment* that encompasses autonomy and control of resources and their relationship with child feeding and nutritional status^(15–17)^. We replaced self-confidence with self-efficacy because of the prominence of the latter in behavioural theory^(18)^ (Table 1).

**Table 1:** Caregiver resources constructs and definitions

Construct	Definition	Boundaries, dimensions and related terms
Self-efficacy	Beliefs about their ability to perform actions that affect children’s health^(3,18,57)^.	Generalised self-efficacy as well as self-efficacy specific to parenting and feeding: Mothering self-efficacy Feeling capable Perceptions of self-worth Self-esteem
Perceived physical health	Perceptions of health and energy level to do daily activities, including caregiving^(3)^.	Perceptions of how health impacts quality of life Excluded: Biomedical measures (e.g. anaemia, BMI)
Mental health	A state of well-being in which individuals can realise their abilities, cope with daily stresses, work and learn productively and make a contribution to their community^(3,63)^.	Depressive symptoms/depression Anxiety Mood disorders Maternal distress Excluded: Biomedical measures (e.g. measure of neurotransmitter levels)
Healthy stress levels	Stress level that is (or perceived to be) low, manageable levels not resulting in chronic activation of the stress response or associated physical, emotional or behavioural symptoms^(3,64,65)^.	General life stresses as well as stresses related to feeding and parenting
Equitable gender attitudes	Attitudes that reflect acceptance of equitable or egalitarian gender norms^(66)^.	Caregivers’ individual attitudes about: Intimate partner violence and domestic violence against women Gender roles and responsibilities Women’s *v*. men’s work Equitable access to education Excluded: Community-level attitudes about gender norms
Safety and security	Feeling safe from any experiences of domestic abuse, including physical, psychological and/or sexual abuse, as well as violence outside the home, including armed conflict, displacement, kidnapping and personal attack^(6)^.	Overall experience of domestic violence Intimate partner violence Excluded: Community/household-level experiences of violence that are not measured at the individual level
Social support	Received or perceived resources and interactions with others that influence a person’s ability to manage a problem or practice a behaviour^(3,60,67–71)^.	Emotional support Informational support Instrumental support (including support with caregiving) Belonging support Social networks Social capital
Time sufficiency	Perception of the adequacy of time to attend to different roles, including time trade-offs and time-use patterns^(3)^.	Time sufficiency Time stress Time-use patterns Workloads Excluded: Employment status (e.g. employed or not employed in specific sectors)

There is strong evidence of the efficacy of nutrition interventions to improve infant and young child health and nutritional status^(4,19)^. Despite this evidence, it has frequently been asked why many programmes do not achieve intended outcomes when implementing nutrition interventions at scale^(20–22)^. As most interventions to improve nutrition require behaviour change on the part of caregivers, interventions may work through caregiver resources (mediation) and leverage caregiver resources (effect modification) to achieve intended outcomes^(10–12,23,24)^. Thus, programme effectiveness could be improved by enhancing caregiver resources and addressing what caregivers need to participate in nutrition programmes and adopt care and feeding recommendations. Although caregiver resources are typically measured at the individual level, they exist within family and community contexts and are influenced by larger systems (Fig. 1). Supportive services and enabling policies and environments offer ways to enhance caregiver resources, facilitating provision of the components of nurturing care. Programmes cannot improve child nutrition without understanding the resources caregivers need to provide nurturing care. Prioritising caregivers and the resources they need acknowledges the value and complexity of providing nurturing care for children and the constraints caregivers face in adopting recommended practices. These caregiver resources not only allow caregivers, who are most frequently women, to care for their children but are also essential for caregivers’ own health and well-being.

**Fig. 1:**
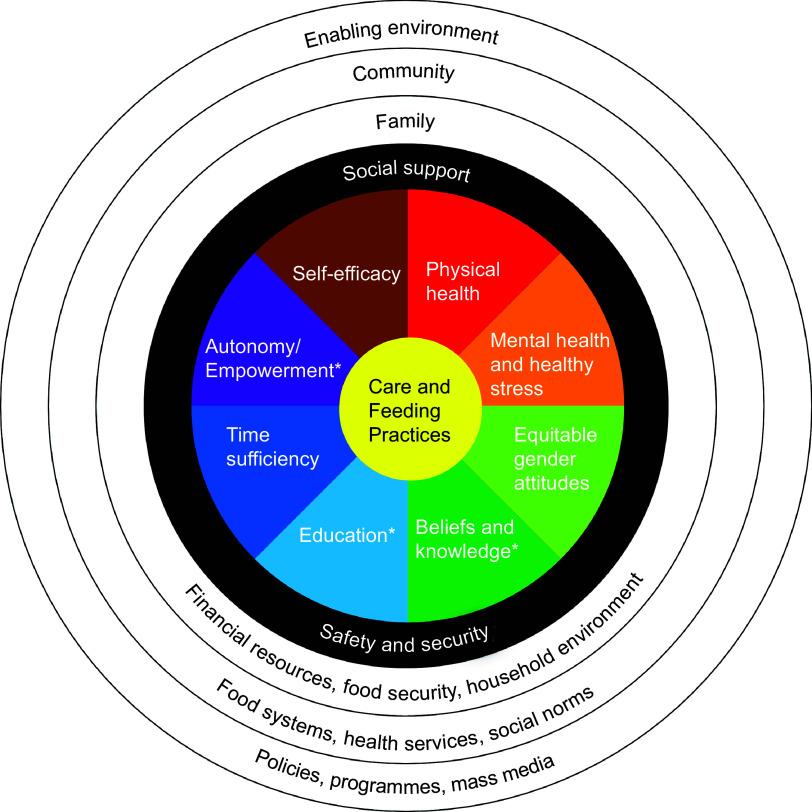
Multilevel factors influence caregiver resources *Caregiver resources not included in this review

Given the critical role of caregiver resources in achieving child health, nutrition and development and the 25 years since Engle *et al.* first presented resources for care, it is remarkable that researchers and programme implementers lack a comprehensive source that (1) details caregiver resources concepts, definitions and measures that have been developed and tested and (2) identifies gaps in the development of adequate measures. To begin to address this gap, we conducted a systematic scoping review to investigate how caregiver resource constructs have been measured in the peer-reviewed literature from low- and lower-middle-income countries. We focus on the complementary feeding period, which requires multiple complex caregiving practices and is a time of high risk for malnutrition and long-term health implications^(25)^.

## Methods

We conducted a scoping review of peer-reviewed publications in low- and lower-middle-income countries to identify quantitative measures of at least one caregiver resource used in the context of studies on complementary feeding or the nutritional status of children 6 months to 2 years of age. We used the Joanna Briggs Institute guidelines to conduct this review^(26)^.

We define caregiver resources as factors *measured* at the level of individual caregivers (although they reflect multilevel factors outside the individual) that influence caregivers’ ability to provide care that produces positive child nutrition, health and development outcomes or to participate in programmes or activities to improve those outcomes. Socio-demographic variables (e.g. age, sex and marital status) do not fit our definition of caregiver resources, although they are related to access to or development of these resources. We selected the following eight caregiver resources constructs as a focus for this review: (1) self-efficacy; (2) perceived physical health; (3) mental health; (4) healthy stress levels; (5) equitable gender attitudes; (6) safety and security; (7) social support and (8) time sufficiency. These constructs are defined in Table 1. We excluded three categories of constructs presented by Engle *et al.* ^(3)^ from our review. Caregiver education was excluded because it is a commonly used socio-demographic variable; knowledge and beliefs were excluded because these measures vary extensively depending on the goal of the programme or research and autonomy and control of resources were excluded because there are reliable recent reviews and analyses of these constructs as dimensions of women’s empowerment^(15–17,27,28)^.

### Search strategy

We systematically searched four digital reference databases on 11 August 2021: CINAHL, PubMed, Scopus and Web of Science. The search terms were restricted to title and abstract words and relevant medical subject heading words or subheadings. The full PubMed search is available (see online supplementary material, Supplemental Table S1). To identify literature published after the Engle *et al.* ^(3)^ article, we searched for peer-reviewed articles published after 1 January 1999. We used the World Bank 1999 country income classifications^(29)^. We also searched the PubMed database to identify articles in five upper-middle income countries (i.e. Botswana, Brazil, Gabon, Mexico and South Africa) where caregiver resources research had been conducted. While these five countries did not meet the World Bank 1999 income classification, some were categorised as lower-middle income not long before or after 1999 and they each had GINI coefficients (reflecting unequal income distribution) similar to included neighbouring countries in Central and Southern Africa and Latin America^(30)^. We reviewed the reference lists of all included articles to identify additional relevant articles. Search results were imported into Covidence Online Software (https://www.covidence.org) to screen articles, extract data and manage the review process.

### Inclusion and exclusion criteria

The search included articles that measured at least one caregiver resource in the context of complementary feeding or child nutritional status from ages 6 months to 2 years. To focus on settings where caregivers were actively engaged in child feeding, we excluded articles that took place in a clinical setting or with participants hospitalised for reasons other than wasting. Articles not available in English were also excluded.

Using an inclusion-criteria checklist, two reviewers independently screened titles and abstracts for inclusion. Titles and abstracts from the PubMed search of the five additional country contexts and those identified through hand search were screened by one author. All articles that passed the title and abstract review were sent to full-text review and were independently evaluated by two authors, using a full-text review checklist. Discordances between the two reviewers during either the title and abstract review or the full-text review were resolved through discussion and consensus with a third reviewer.

To strengthen reliability between reviewers, a series of training exercises was performed before beginning each stage of the review. Three rounds of practice were conducted for title and abstract screening on a sample of 200 citations.

### Data extraction

Data were extracted on study characteristics, objectives, development and properties of caregiver resources measures and results related to caregiver resources, complementary feeding and nutrition outcomes. If reported, data regarding the following psychometric properties were extracted: face validity, content validity, construct validity, criterion validity, internal consistency, test-retest reliability, predictive validity, responsiveness, acceptability, reliability, feasibility, revalidation and cross-cultural adaptation. Data extraction was managed in Covidence Online Software.

## Results

**Fig. 2:**
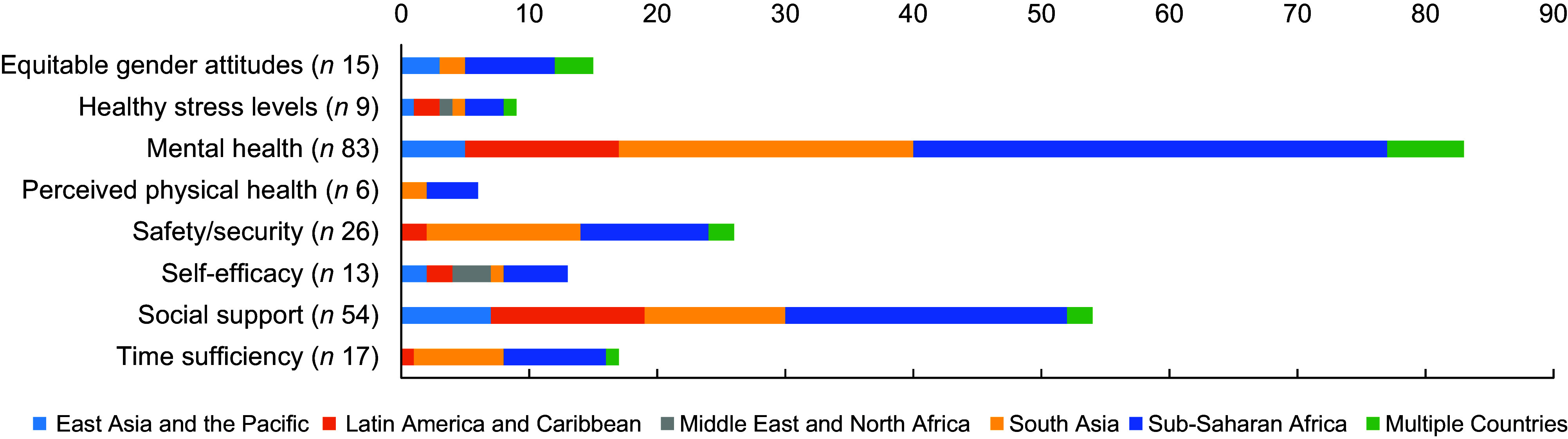
Number of included articles by caregiver resource construct and World Bank region (*N* 163)

**Fig. 3:**
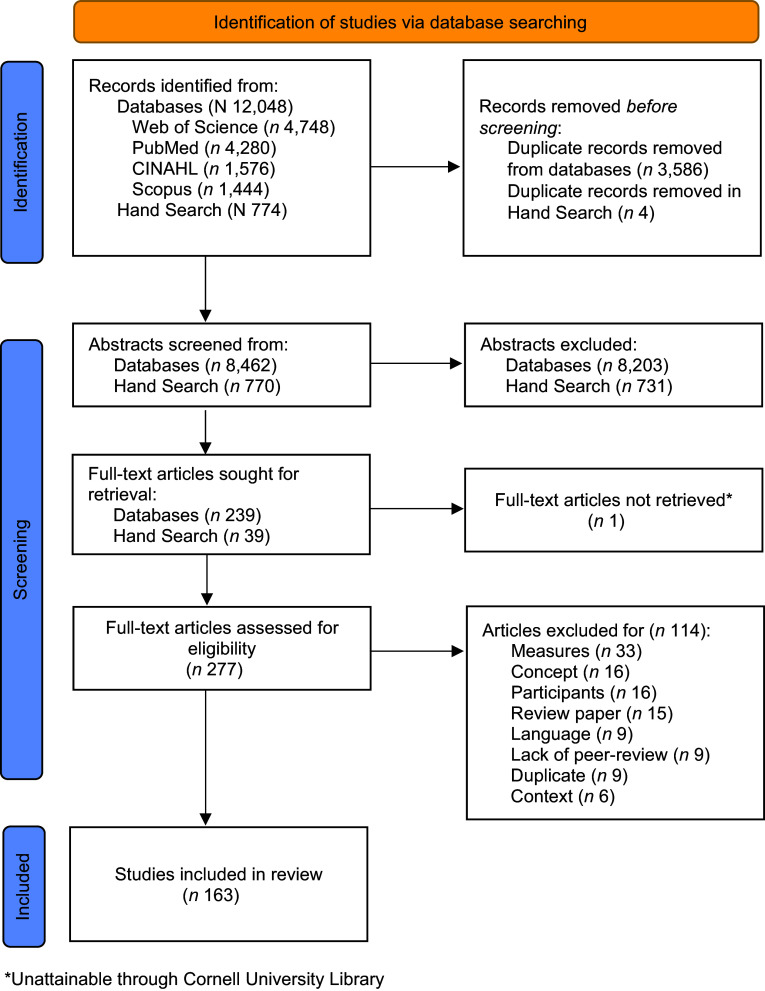
PRISMA flow diagram of systematic search, screening and selection of articles

We identified 163 articles that measured at least one caregiver resource in relation to complementary feeding or the nutritional status of children 6 months to 2 years of age (Fig. 2). Two-thirds of included articles measured caregiver resources in sub-Saharan Africa or South Asia (Fig. 3). Most articles (*n* 125; 77 %) measured only one caregiver resource (see online supplementary material, Supplemental Fig. S1). Table 2 provides a summary of the measurement of each caregiver resources construct and the frequency of adaptation and psychometric testing. Mental health or social support, or both, was measured in eighty-three, fifty-four and twenty-four articles, respectively. The caregiver resource measured least often was perceived physical health (*n* 6) (Table 2). See online supplementary material, Supplemental Table S3 for an overview of each caregiver resource measure used in the articles in our review including country, description, number of items, formative research used, adaptations made and cognitive interviewing, pretesting and psychometric assessments conducted. See online supplementary material, Supplemental Table S4 for a summary of each article, including design, sample size, participant characteristics and related findings.

**Table 2:** Summary of caregiver resource construct measures used during the complementary feeding period

			Number of articles reporting					
Construct	Number of measures	Number of articles	Formative research	Adaptation	Cognitive interviews	Pretesting	Validity testing	Reliability testing
Self-efficacy	11	13	0	4	4	5	1	3
Perceived physical health	4	6	0	1	2	2	1	2
Mental health	21	83	0	9	2	6	15	12
Healthy stress levels	8	9	0	1	1	0	4	4
Equitable gender attitudes	6	15	0	2	1	5	1	1
Safety and security	14	26	1	5	0	6	1	2
Social support	46	54	3	10	2	4	7	7
Time sufficiency	9	17	1	1	1	5	0	1

Shading represents percent of measures that were developed or adapted using formative research, adapted from an existing measure, or reported conducting psychometric assessments related to validity and reliability. 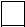, 0–20%; 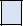, 21–40%; 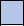, 41–60%; 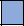, 61–80%; 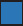, 81–100%.

### Self-efficacy

We identified thirteen articles that measured self-efficacy (Table 2). Most used the term *self-efficacy* with or without specification (e.g. maternal, parenting, infant care and complementary feeding). Other terms used that fit our definition of self-efficacy included: parenting self-esteem, perceived behavioural control and social power. In most articles (*n* 8), authors developed their own measure of self-efficacy, but three articles reported adapting pre-existing measures of self-efficacy (see online supplementary material, Supplemental Table S3). Several articles (*n* 7) reported finalising measures after pretesting, discussions with experts and qualitative interviews.

### Perceived physical health

Six articles measured perceived physical health, all of which used pre-existing tools (see online supplementary material, Supplemental Table S3). Most articles (*n* 5) reported adapting, translating and/or pretesting the pre-existing tool to meet population needs.

### Mental health

We identified eighty-three articles that measured maternal or parental mental health (Table 2). Most often, articles assessed maternal depression, depressive symptoms or depressed mood (*n* 43) (see online supplementary material, Supplemental Table S3). Articles also measured common mental disorders (*n* 13), maternal psychological distress (*n* 8), postpartum depression (*n* 7) or risk of common mental disorders or probable depression (*n* 2). Others measured psychological well-being (*n* 2), overall maternal mental health (*n* 5) or parental mental health (*n* 1). Four pre-existing instruments were commonly used: Self-Reporting Questionnaire-20 (*n* 23)^(31)^; Edinburgh Postnatal Depression Scale (*n* 16)^(32)^; Center for Epidemiological Studies Depression Scale (*n* 13)^(33)^ and Patient Health Questionnaire-9 (*n* 7)^(34)^. Other pre-existing, validated measures of mental health were used in twenty-one articles. In almost half of the articles using pre-existing measures (*n* 37), authors describe pretesting and adaptation, including translation and cultural adaptations. Two articles used author-developed measures of life satisfaction^(35,36)^ and a suffering scale pictogram^(37)^.

### Healthy stress levels

Nine articles measured types and levels of perceived stress. Maternal stress and distress were the most common terms used to describe stress; other terms included parenting stress, caregiver stress regarding feeding, economic stress, partner stress, domestic violence, community violence and worry (see online supplementary material, Supplemental Table S3). Pre-existing measures were used in six articles and author-developed measures were used in three articles. Two articles described adapting pre-existing measures for the context.

### Equitable gender attitudes

We identified fifteen articles that measured gender attitudes (Table 2). Twelve of these included a construct related to women’s acceptance of domestic violence, typically using an original or adapted version of a measure from the Demographic and Health Survey, Multiple Indicator Survey, or India’s National Family Health Survey. All three of these nationally representative cross-sectional surveys assess women’s views on whether domestic violence (or wife beating) is justified in certain scenarios^(38–40)^ (see online supplementary material, Supplemental Table S3). Each of these surveys is translated and pretested in each country’s context and questions remain the same from year to year to allow for comparison over time.

### Safety and security

We identified twenty-six articles that measured aspects of safety and security. Most measured women’s overall experience of domestic violence or intimate partner violence (distinct from the previous construct which focused on *attitudes* related to intimate partner violence), including controlling behaviour, emotional violence, sexual violence or physical violence (see online supplementary material, Supplemental Table S3). Sixteen articles used the original or an abbreviated version of the domestic violence module in the Demographic and Health Survey and India’s National Family Health Survey, which uses a shortened, adapted version of the Conflict Tactics Scale from the WHO multi-country study on women’s health and domestic violence to measure spousal violence^(41,42)^. Several articles only used a shortened or modified version of the Conflict Tactics Scale^(41)^. Six articles used author-developed measures. In most articles, authors described methods of adaptation, translation or pretesting the measure (Table 2).

### Social support

We identified fifty-four articles that measured social support, which included dimensions of social support (e.g. informational, emotional and instrumental) (*n* 26), social networks (*n* 8) and social capital (*n* 9) (see online supplementary material, Supplemental Table S3). Existing, validated, general social support measures^(43–45)^ were used in seven articles. Most articles used author-developed social support measures to assess whether mothers/caregivers received support in general, from certain individuals (e.g. husbands, grandmothers), or for specific tasks (e.g. child care, child feeding and household chores). Two articles also collected data from the people providing support (i.e. fathers and grandmothers). Women’s social capital was measured most frequently using the Short Social Capital Assessment Tool^(46)^. One article examined fathers’ social capital. In several articles, authors used a single proxy measure of social capital (e.g. group membership, social participation and religious affiliation). Social network measures varied considerably. Typically, they measured the general composition and size of women’s networks, though two articles used measures that asked about the number of network members who adopted recommended infant-feeding practices or with whom they discussed infant feeding (see online supplementary material, Supplemental Table S3). Of the fifty-four articles that measured social support constructs, twenty-four reported adapting, pretesting, using a previously validated scale or using previous research to inform the development of their measure for their context (see online supplementary material, Supplemental Table S4).

### Time sufficiency

We identified seventeen articles with measures related to time sufficiency (Table 2), ranging from a single question to extensive 24-h recalls of time spent on and/or frequency of one or more activities (e.g. agricultural or productive work, childcare or domestic activities or leisure). One article used observation to document time allocation^(47)^. Most articles measured time use or workload rather than time sufficiency *per se*, asking about frequency and amount of time spent on different activities to estimate totals or patterns of time use over days, weeks or seasons. One measure asked women specifically about time stress^(14,48)^. Several articles asked about satisfaction with amount of leisure time and/or set a cut-off for designating excessive workloads or time poverty. This is the approach to measuring time use in the Women's Empowerment in Agriculture Index (WEAI) ^(49)^. Eight articles involved secondary analysis of data collected with the WEAI. Few articles described development of measures of time use, beyond pretesting or adaptation of pre-existing measures such as WEAI (see online supplementary material, Supplemental Table S4). Several authors noted that time allocated to care is often underreported because caregiving is undertaken simultaneously with other domestic or productive activities.

### Psychometric properties

There was considerable variation in the presentation of psychometric properties both between and within caregiver resources constructs (see online supplementary material, Supplemental Table S4). Most articles that measured mental health (*n* 51 of 83) reported or cited previously assessed psychometric properties of the measure. It was less common to report psychometric testing or previous validation activities for other constructs.

### Relationships between caregiver resources and child nutrition outcomes and complementary feeding practices

While not the focus of this review, we summarised the findings on relationships between caregiver resources and complementary feeding practices or child nutrition outcomes (see online supplementary material, Supplemental Table S4). There were fairly consistent significant positive relationships between self-efficacy, mental health and safety and security (operationalised as intimate partner violence) and complementary feeding or nutrition outcomes. In contrast, the relationships between perceived physical health, stress, social support, time sufficiency and complementary feeding or nutrition outcomes were mixed.

## Discussion

In this review, we identified a range of measures for eight caregiver resource constructs assessed in the context of complementary feeding in low- and lower-middle-income countries. Though the importance of caregiver resources in child nutrition, health and development is documented in seminal frameworks^(4–7,50)^, there is inconsistency in whether, how and when caregiver resources are measured and reported. By collating evidence of existing measures, this review informs efforts to assess, and thereby investigate the impact of, caregiver resources. The available information on measures varied substantially by construct. Often, even when a caregiver resource was measured, little information was reported on how the measure was developed. Lack of reporting on these measures and how constructs are conceptualised and operationalised in context limits understanding of caregiver resources and the ability to use them in research and evaluation. There is a need for thorough and transparent reporting of how caregiver resource constructs are measured.

In addition to inconsistent reporting, the quality of the measures themselves varied substantially. Although several constructs (i.e. mental health, equitable gender attitudes, safety and security and time allocation) were measured in relatively consistent ways, others lacked standardised measures that can be applied in cross-cultural contexts. Some articles, particularly those based on large data sets such as the Demographic and Health Survey, used proxies to assess caregiver resources constructs. For example, most measures of equitable gender attitudes assessed women’s attitudes towards gender-based violence. However, conceptually, the construct applies more broadly to views of the equal status between genders – rights, roles and responsibilities and access to power and resources, which influence care and feeding practices. In some cases, attitudes towards domestic violence were used as a proxy for women’s self-esteem and empowerment. Similarly, intimate partner violence was typically measured rather than all aspects of safety and security. Proxies for social support were also common, and these measures often did not adequately reflect the social support construct. Overall, lack of consistency in how constructs are conceptualised, measured and reported inhibits their potential to inform and strengthen interventions.

For most constructs, measures were not specific to complementary feeding or child caregiving, even though our search included only papers with this focus. For self-efficacy, however, caregiving or complementary feeding-specific measures were used. Bandura^(56,57)^ promoted the use of domain- and task-specific measures for self-efficacy. As such, self-efficacy measures often assessed maternal, parenting or caregiving self-efficacy, but it was less common to measure self-efficacy for complementary feeding. This contrasts with breast-feeding self-efficacy research, which has several scales validated in multiple contexts^(58,59)^. This highlights the need to develop validated complementary feeding self-efficacy scales. Most measures of social support assessed support in general, with few focused on support for child care or complementary feeding. Contextually appropriate, validated measures of social support specific to complementary feeding are needed, as studies that measure behaviour-specific social support have found stronger associations with health outcomes when compared with general social support measures^(60)^. Similar measures for breast-feeding-specific social support exist^(61,62)^. Measures of time use are also not specific to feeding and nutrition-related care practices. Measures often included an assessment of time spent on caregiving in general, such as in the Women’s Empowerment in Agriculture Index^(49)^, which included caregiving for children and the elderly. Limited time is a well-documented barrier to optimal care and feeding practices^(5)^, but measuring time for infant and young child care and feeding continues to be a challenge, and a specific measure is needed to assess trade-offs between caregivers’ other responsibilities and caregiving.

Scale development and validation ensure tools accurately and reliably measure intended outcomes^(61)^. Adaptation enables researchers to contextualise a tool to a given setting; however, few articles reported adapting measures using methods such as formative research, cognitive interviewing, pretesting or cross-cultural equivalency. When standardised tools are used, it is important to contextualise the items within a measure to specific settings, as is done in the National Family Health Survey in India^(39)^. Although existing measures for social support have been adapted and validated in multiple contexts, author-developed measures of social support were used most often, and the process for their development and validation was rarely described. Time allocation measures must be adapted to fit the usual activities of caregivers, which vary considerably by context, particularly between rural and urban areas. Time sufficiency or time use is challenging to measure due to daily and seasonal variability, difficulty in estimating time spent on informal or unstructured work and the large number of activities people engage in, sometimes concurrently.

Most measures we identified assessed a deficiency or a problem, with researchers using terms such as *time poverty* (rather than time sufficiency) or *violence* (rather than safety and security). We reframed these constructs with positive labels to acknowledge the capabilities that people bring to the caregiving role and avoid blaming individuals or highlighting deficiencies that may originate in social and environmental constraints. This framing is consistent with the updated UNICEF Nutrition Conceptual Framework^(7)^.

Caregiver resources affect maternal and child nutrition broadly; however, we limited our review to articles about complementary feeding and child nutrition status from 6 months to 2 years of age. It is likely that there are many existing measures of caregiver resources constructs that have not been used in complementary feeding and child nutrition research but are applicable to this developmental stage. Our focus on nutrition and complementary feeding may have omitted relevant measures. However, our focus was intended to gauge the scope of attention being paid to caregiver resources constructs in complementary feeding research and programmes. There is considerable research investigating the relationship between individual caregiver resources and breast-feeding, and it is likely that specific measures, particularly those related to breast-feeding self-efficacy^(59)^, knowledge and social support^(62)^ that were not captured in this review may be relevant to additional aspects of maternal and child nutrition. Our review focused on low- and lower-middle-income countries. Other measures used in upper-middle- or high-income countries may provide tools that can be adapted, but this review provides a sense of the degree to which caregiver resources are measured in low- and lower-middle-income countries. Although this focus helps narrow the measures to those more likely to be appropriate in these settings, we note the limited detail provided on adaptation and testing in different contexts and the lack of psychometric testing reported. Given recent reviews of women’s empowerment and child nutritional status^(15–17,27,28)^, we did not include articles related to women’s empowerment. These extensive reviews likely capture many of the relevant measures; however, there may be articles published after these reviews that included relevant measures which are not included in this review.

### Conclusion

While many nutrition interventions focus on caregiver knowledge and beliefs, other intangible caregiver resources such as self-efficacy, physical health, mental health, healthy stress levels, equitable gender attitudes, time sufficiency, social support, safety and security and empowerment are integral to optimal complementary feeding practices. This review identified measures of caregiver resources to facilitate future research and programme evaluation about how these factors influence participation in nutrition programmes and the adoption of complementary feeding recommendations. Caregiver resources are relevant to multiple aspects of household well-being, such that strengthening caregiver resources provides a lever by which the uptake and effectiveness of multifaceted interventions can be improved.

Measurement of caregiver resources during the complementary feeding period is limited. Developing, adapting, testing and utilising measures of caregiver resources are essential for understanding caregivers’ ability to adopt complementary feeding recommendations. We found wide variation in measurement approaches. This summary is a first step towards more widespread, careful and validated measures to assess caregiver resources, the foundation on which improved nutritional practices are built. A framework that highlights the resources caregivers bring to child nurturance may facilitate a shift away from deficit models used to explain lack of uptake of social and behaviour change nutrition interventions and identify strategies to build resources, prioritise caregivers and strengthen caregiver resources to increase intervention effectiveness.

## Supporting information

Martin et al. supplementary material Martin et al. supplementary material

## Supplementary material

For supplementary material accompanying this paper visit https://doi.org/10.1017/S1368980024000065
