# Supplementary material for: Measuring the intangible resources caregivers need to provide nurturing care during the complementary feeding period: a scoping review in low- and lower-middle-income countries
Source: Public Health Nutr. 2024 Jan 15;27(1):e78. doi: 10.1017/S1368980024000065 (PMC10966882; doi:10.1017/S1368980024000065)
Supplement: Martin et al. supplementary material [file S1368980024000065sup001.pdf]

Supplementary Materials

Figure S1. Number of caregiver resource constructs measured in each article

Table S1. PubMed search strategy

Table S2. Scoping review inclusion and exclusion criteria

Table S3. Description of caregiver resource measures included in this review

Table S4. Caregiver Resource measures and findings in complementary feeding and responsive feeding articles

References for supplementary materials

Figure S1. Number of caregiver resource constructs measured in each article (N=163)

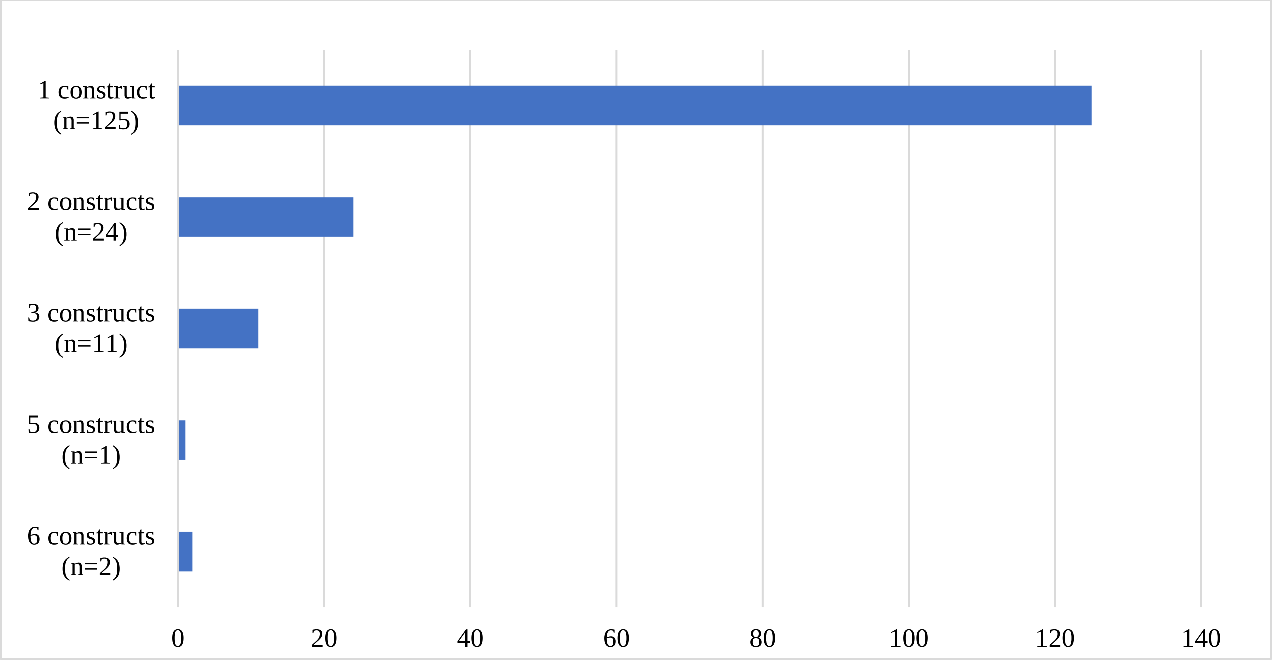

Table S1: PubMed search strategy

Search domain	Search terms
#1 Participants	infant*[tiab] OR infancy[tiab] OR child*[tiab] OR baby[tiab] OR babies[tiab] OR babys[tiab] OR toddler*[tiab] OR "first 1000 days"[tiab] OR "first 1,000 days"[tiab] OR "child"[MeSH Terms]
#2 Caregiver resources	((mother*[tiab] OR maternal[tiab] OR caregiver*[tiab]) AND (capabilit*[tiab] OR capacit*[tiab])) OR “gender norm attitudes”[tiab] OR “gender equality”[tiab] OR “gender inequality” [tiab] OR “gender role”[tiab] OR “gender roles”[tiab] OR “gender norm”[tiab] OR “gender norms”[tiab] OR “gender relations”[tiab] OR “women's status”[tiab] OR “woman's status”[tiab] OR “status of women”[tiab] OR “egalitarian beliefs”[tiab] OR stress*[tiab] NOT “oxidative stress”[tiab] OR “mental health”[tiab] OR “depressive symptoms”[tiab] OR depression[tiab] OR anxiety[tiab] OR anxious[tiab] OR “mood disorder”[tiab] OR “mood disorders”[tiab] OR “cope”[tiab] OR “coping”[tiab] OR trauma*[tiab] OR “psychological health”[tiab] OR “psychological wellbeing”[tiab] OR “psychological well being”[tiab] OR “psychological well-being”[tiab] OR “maternal distress”[tiab] OR helplessness[tiab] OR passivity[tiab] OR "depression"[MeSH Terms] OR "depressive disorder"[MeSH Terms] OR "anxiety"[MeSH Terms] OR “perceived health”[tiab] OR “self-reported health”[tiab] OR “self reported health”[tiab] OR ((“perceived”[tiab] OR “self-reported”[tiab] OR “self reported”[tiab]) AND "physical health"[tiab]) OR "wellbeing"[tiab] OR "well-being"[tiab] OR "well being"[tiab] OR "quality of life"[tiab] OR "intimate partner violence"[Title/Abstract] OR "domestic violence"[Title/Abstract] OR "domestic abuse"[Title/Abstract] OR "violence against women"[Title/Abstract] OR "gender-based violence"[Title/Abstract] OR "gender-based violence" [Title/Abstract] OR "gendered violence"[Title/Abstract] OR "sexual abuse"[Title/Abstract] OR "physical abuse"[Title/Abstract] OR "psychological abuse"[Title/Abstract] OR "emotional abuse"[Title/Abstract] OR "emotional violence"[Title/Abstract] OR "harassment"[Title/Abstract] OR "humanitarian"[Title/Abstract] OR "displace*" [Title/Abstract] OR "emergenc*"[Title/Abstract] OR "war"[Title/Abstract] OR "wars"[Title/Abstract] OR "kidnap*"[Title/Abstract] OR "armed conflict"[Title/Abstract] OR “emergencies"[MeSH Terms] OR "armed conflicts"[MeSH Terms] OR "violence"[MeSH Terms] OR “self-efficacy”[tiab] OR “self efficacy”[tiab] OR “capable”[tiab] OR “parenting efficacy”[tiab] OR “self-worth”[tiab] OR “self worth”[tiab] OR “self-confidence”[tiab] OR “self confidence”[tiab] OR “confident”[tiab] OR “self esteem”[tiab] OR “self-esteem”[tiab] OR "self efficacy"[MeSH Terms] OR "self concept"[MeSH Terms] OR “social support”[tiab] OR “social networks”[tiab] OR “social network”[tiab] OR “social capital”[tiab] OR loneliness[tiab] OR supportiveness[tiab] OR “emotional support”[tiab] OR “practical support”[tiab] OR “material support”[tiab] OR “informational support”[tiab] OR “instrumental support”[tiab] OR “appraisal support”[tiab] OR “family support”[tiab] OR "social support"[MeSH Terms] OR “time use”[tiab] OR “time to”[tiab] OR “time availability”[tiab] OR “time burden”[tiab] OR “time trade-off”[tiab] OR “time trade off”[tiab] OR “workload”[tiab] OR “work burden”[tiab] OR “work hours”[tiab] OR "women's work*"[tiab] OR "woman's work*"[tiab] OR “men’s work*"[tiab] OR “man’s work*"[tiab] OR "maternal work*"[tiab] OR "mother's work*"[tiab] OR "mothers' work*"[tiab] OR "mothers work*"[tiab] OR “father’s work*”[tiab] OR “fathers’ work*”[tiab] OR “fathers work*”[tiab] OR "parent's work*"[tiab] OR “parents’ work*”[tiab] OR “parents work*”[tiab] OR "parental work*"[tiab] OR “paternal work*"[tiab] OR “caregiver’s work*”[tiab] OR “caregivers’ work*”[tiab] OR “caregivers work*”[tiab] OR "women's time"[tiab] OR "woman's time"[tiab] OR "mother's time"[tiab] OR "mothers time"[tiab] OR "mothers' time"[tiab] OR "maternal time"[tiab] OR “father’s time”[tiab] OR “fathers’ time”[tiab] OR “fathers time”[tiab] OR "parental time"[tiab] OR “parents’ time”[tiab] OR “parents work”[tiab] OR “caregiver’s time”[tiab] OR “caregivers’ time”[tiab] OR “caregivers time”[tiab]
#3 Child feeding and nutrition	“complementary feeding”[tiab] OR “complementary food”[tiab] OR “complementary foods”[tiab] OR “weaning”[tiab] OR food*[tiab] OR diet*[tiab] OR meal*[tiab] OR intake*[tiab] OR “feeding frequency”[tiab] OR “responsive child feeding”[tiab] OR “responsive feeding”[tiab] OR “responsive parenting”[tiab] OR “active feeding”[tiab] OR “hunger cue*”[tiab] OR “satiety cue*”[tiab] OR stunt*[tiab] OR wasted[tiab] OR wasting[tiab] OR underweight[tiab] OR undernutrition[tiab] OR undernourished[tiab] OR malnutrition[tiab] OR malnourished[tiab] OR “micronutrient deficiency”[tiab] OR “micronutrient deficiencies”[tiab] OR anemi*[tiab] OR “nutritional status”[tiab] OR "diet"[MeSH Terms] OR "food"[MeSH Terms] OR "growth disorders"[MeSH Terms] OR "malnutrition"[MeSH Terms] OR "micronutrients"[MeSH Terms] OR "deficiency"[MeSH Subheading] OR "infant nutritional physiological phenomena"[MeSH Terms]
#4 Context	low-income countr*[tiab] OR low income countr*[tiab] OR lower-middle-income countr*[tiab] OR lower middle income countr*[tiab] OR low- and middle-income countr*[tiab] OR low and middle-income countr*[tiab] OR low and middle income countr*[tiab] OR developing countr*[tiab] OR afghan*[tiab] OR albania*[tiab] OR algeria*[tiab] OR angola*[tiab] OR armenia*[tiab] OR Azerbaijan*[tiab] OR bangladesh[tiab] OR belarus*[tiab] OR belize*[tiab] OR benin*[tiab] OR Bhutan*[tiab] OR Bolivia*[tiab] OR bosnia*[tiab] OR “Bosnia and Herzegovina”[tiab] OR Bulgaria*[tiab] OR “Burkina Faso”[tiab] OR Burundi*[tiab] OR “Cabo Verde*”[tiab] OR “Cape verde*”[tiab] OR Cambodia*[tiab] OR Cameroon*[tiab] OR “Central African Republic”[tiab] OR chad*[tiab] OR china*[tiab] OR colombia*[tiab] OR comoros[tiab] OR comores[tiab] OR comoro[tiab] OR congo[tiab] OR “costa rica”[tiab] OR “Côte d'Ivoire”[tiab] OR “Ivory Coast”[tiab] OR cuba*[tiab] OR Djibouti*[tiab] OR “Dominican Republic”[tiab] OR Ecuador*[tiab] OR Egypt*[tiab] OR “El Salvador”[tiab] OR “Equatorial Guinea”[tiab] OR Eritrea*[tiab] OR Eswatini*[tiab] OR Ethiopia*[tiab] OR fiji[tiab] OR gambia*[tiab] OR gaza*[tiab] OR Georgia*[tiab] OR Ghana*[tiab] OR Guatemala*[tiab] OR guinea*[tiab] OR guyana[tiab] OR haiti[tiab] OR hondura*[tiab] OR india*[tiab] OR Indonesia*[tiab] OR iran*[tiab] OR Iraq*[tiab] OR Jamaica*[tiab] OR Jordan*[tiab] OR Kazakhstan*[tiab] OR Kenya*[tiab] OR Kiribati*[tiab] OR korea*[tiab] OR kyrgyz[tiab] OR kirghizia[tiab] OR kirghiz[tiab] OR kirgizstan[tiab] OR kyrgyzstan[tiab]

	OR “Lao PDR”[tiab] OR laos[tiab] OR Latvia*[tiab] OR lesotho[tiab] OR Liberia*[tiab] OR Lithuania*[tiab] OR madagascar[tiab] OR Malawi*[tiab] OR maldiv*[tiab] OR mali[tiab] OR “Marshall Islands”[tiab] OR Mauritania*[tiab] OR Micronesia*[tiab] OR moldova*[tiab] OR Mongolia*[tiab] OR morocc*[tiab] OR mozambique[tiab] OR myanmar[tiab] OR Namibia*[tiab] OR nepal[tiab] OR nicaragua[tiab] OR niger*[tiab] OR Nigeria*[tiab] OR “North Macedonia”[tiab] OR macedonia*[tiab] OR Pakistan*[tiab] OR “Papua New Guinea”[tiab] OR paraguay[tiab] OR peru*[tiab] OR philippines[tiab] OR phillippines[tiab] OR philipines[tiab] OR phillipines[tiab] OR principe[tiab] OR Romania*[tiab] OR russia*[tiab] OR Rwanda*[tiab] OR ruanda[tiab] OR samoa*[tiab] OR “Sao Tome”[tiab] OR Senegal*[tiab] OR “Sierra Leone”[tiab] OR “Solomon Islands”[tiab] OR somalia[tiab] OR “Sri Lanka”[tiab] OR “St. Vincent and the Grenadines”[tiab] OR sudan*[tiab] OR suriname*[tiab] OR Syria*[tiab] OR “Syrian Arab Republic”[tiab] OR tajikistan[tiab] OR tadzhikistan[tiab] OR tadjikistan[tiab] OR tadzhik[tiab] OR Tanzania*[tiab] OR Thai*[tiab] OR togo[tiab] OR tonga[tiab] OR turk*[tiab] OR Uganda*[tiab] OR ukrain*[tiab] OR uzbek[tiab] OR uzbekistan[tiab] OR vanuatu[tiab] OR Vietnam*[tiab] OR “West Bank”[tiab] OR yemen[tiab] OR Zambia*[tiab] OR Zimbabwe[tiab] OR "developing countries"[MeSH Terms]
#5 Publication date	[Filters: from 1999-2021]
Full search	#1 AND #2 AND #3 AND #4 AND #5

Table S2. Scoping review inclusion and exclusion criteria

Inclusion criteria	Exclusion criteria
• Publication date: January 1999 or later<br>• Conducted in low- or lower-middle–income country (per 1999 World Bank classification) and five upper-middle–income countries relevant for a global nutrition-programming context<br>• Mentions, discusses, or reports complementary feeding at ages 6–23 months or child nutritional status from 6 months to 2 years<br>• Uses quantitative methods to measure at least one caregiver resource: equitable gender attitudes, healthy stress levels, mental health, perceived physical health, safety and security, self-efficacy, social support, time sufficiency	• Article not available in English<br>• Participants are not home-based caregivers (e.g., health clinic or hospital staff or daycare)<br>• Participants are hospitalized populations, except those being treated for severe acute malnutrition

Table S3. Description of caregiver resource measures included in this review

Note: Dots note that authors reported adapting an existing measure or conducting cognitive interviews, pretesting, or psychometric assessments related to validity and reliability. Dots do not reflect the quality or the results of the adaptation, pretesting or assessment.

Measure	Countries where measure use	Measure description	Items	Adaptation	Cog. Interviews	Pretesting	Validity	Reliability
**Self-efficacy (n=13)**								
Maternal Self-Efficacy for Complementary Feeding (1)	**Bangladesh** (1)	Maternal self-efficacy for feeding family cooked foods, avoiding store-bought snacks, deciding on types of food to feed a child, and raising a healthy child.	4		•			•
Maternal Self-Efficacy Scale (2)	**Brazil** (3); **Iran** (4)	Mothers perceived beliefs regarding their performance of specific caregiving tasks.	10	•		•	•	•
Mothering Self-Efficacy (5)	**Zimbabwe** (6,7)	Women's belief in their competence as a mother. Adapted from the Parenting Self-Agency Measure (8) and the Parenting Sense of Competence Scale (9).	10	•	•	•		•
Parenting Self-Esteem (10)	**Jamaica** (10)	Perceived self-efficacy and satisfaction derived from parenting.	10			•		
Perceived Behavioral Control (PBC) (11)	**Kenya** (12)	Perceived behavioral control (ability to perform a behavior) by including items related to perceived self-efficacy and controllability.	6	•		•		
Perceived Self-Efficacy (13)	**Burkina Faso** (13)	Asked women whether they felt capable of giving key food groups (meat, fish or poultry, dark green leafy vegetables, and carrot, squash or sweet potato) to their child daily.	3					
Self-Efficacy (14)	**Iran** (14)	Self-efficacy was a subscale of a larger instrument on Social Cognitive Theory related to complementary feeding practices.	5		•	•		
Self-Efficacy (15)	**Lao PDR** (15)	Mother's self-efficacy to access health services and borrow money when needed.	2					
Self-Efficacy (16)	**Iran** (16)	Self-efficacy regarding complementary feeding of their infants	13	•				
Self-Efficacy in Infant Care Scale (SICS) (17)	**Thailand** (17)	Five domains of infant-care tasks including child development, interaction, general healthcare, safety, and diet.	67		•			
Social power (18)	**Mali** (18)	Operationalized self-efficacy as felt control (i.e. in charge and action-oriented) and passivity/helplessness (i.e. lower perceived self-efficacy).	20					
**Perceived Physical Health (n=6)**								
Euro-Qol (EQ 5D) (19)	**Pakistan** (20)	Five health dimensions including mobility, usual activities, pain/discomfort, self-care, and anxiety/depression.	5					

Measure	Countries where measure use	Measure description	Items	Adaptation	Cog. Interviews	Pretesting	Validity	Reliability
RAND 36-item Health Survey (21)	**Zimbabwe** (6,7)	Quality of life with eight health concepts: physical functioning, role limitations caused by physical health problems, role limitations caused by emotional problems, social functioning, emotional well-being, energy/fatigue, pain, and general health perceptions.	11	•	•	•		•
PedsQL Family Impact Module (22)	**Ghana** (23,24)	Physical health across six self-reported functioning subscales (physical, emotional, social, cognitive, worry and communication) and two-family functioning subscales (daily activities, family relationships).	36		•	•	•	•
WHO Quality of Life (WHOQoL) (25)	**Pakistan** (26)	Participants' physical health, psychological health, social relationships, and environment in relation to their goals, expectations, standards, and concerns in the context of culture and value systems.	100					
**Mental Health (n=83)**								
Aga Khan University Anxiety and Depression Scale (27)	**Pakistan** (28)	Maternal somatic and psychological depressive symptoms.	25				•	
Beck Depression Inventory (29)	**Brazil** (30)	Levels of maternal depression	63				•	•
Beck Depression Inventory Second Edition (BDI II) (31)	**Malaysia** (32)	Reflections on emotional state over the past two weeks, including 21 symptoms of depression.	21				•	•
Center for Epidemiological Studies-Depression (CES-D) (33)	**Antigua** (34) **Bangladesh** (35–37); **Brazil** (3,38); **Ethiopia** (39); **Ghana** (40,41); **India** (42); **Jamaica** (10,34); **St. Lucia's** (34); **Tanzania** (43)	Maternal mental health as it relates to the ability to be emotionally available for her child, including six aspects of depression (depressed mood, guilt/worthlessness, helplessness/hopelessness, lethargy/fatigue, loss of appetite, and sleep disturbance).	20	•	•	•	•	•
Everyday Feeling Questionnaire (EFQ) (44)	**Vietnam** (45)	Psychological well-being over the past four weeks in terms of symptoms of anxiety and depression.	20				•	•
Edinburgh Postnatal Depression Scale (EPDS) (46)	**Bangladesh** (47,48); **China** (49); **Ethiopia** (50); **Gambia** (51); **India** (52,53); **Pakistan** (20); **South Africa** (54–57); **Tanzania** (58); **Zimbabwe** (6,7)	Symptoms of depressed mood including, lack of enjoyment, blaming oneself, anxiousness (including panic), worry, being overwhelmed, difficulty sleeping, sadness, thoughts of self-harm.	10	•		•	•	•

Measure	Countries where measure use	Measure description	Items	Adaptation	Cog. Interviews	Pretesting	Validity	Reliability
General Health Questionnaire (GHQ-12) (59)	**Brazil** (60,61)**; South Africa** (62)	Maternal mental health across three classifications: common mental disorders, severe mental disorders, and depression. Designed to be used by general practitioners and non-psychiatrists.	12	•		•	•	
Hamilton Depression Rating Scale (63)	**Pakistan** (20,64,65)	Symptoms of depression experienced over the previous week.	17					
Hospital Anxiety and Depression Scale (66)	**Pakistan** (67)	Depression and anxiety in the setting of a hospital or medical outpatient clinic	21					
Hopkins Symptom Checklist (HSCL) (68)	**Ethiopia** (69); **Rwanda** (70); **South Africa** (71); **Tanzania** (72)	Maternal distress in terms of symptoms of depression, anxiety, and somatic distress.	58	•			•	•
Life Satisfaction Interview (73)	**Chad** (73)	Caregiver-reported satisfaction with life as a proxy for mental health and lack of stress.	NR					
Life Satisfaction Scale (74)	**India** (74)	Mothers' happiness and satisfaction with married life as a proxy measure for the mother's stress and mental health.	NR			•		
PedsQL Family Impact Module (22)	**Ghana** (24)	Impact of pediatric conditions on multiple dimensions of caregiver quality of life, including physical, emotional, social, and cognitive functioning, worry, communication, family daily activities and family relationships	36	•	•			
Patient Health Questionnaire (PHQ-9) (75)	**Botswana** (76); **Ethiopia** (77); **Kenya** (78); **Nepal** (79); **Peru** (80); **Sudan** (81); **Tanzania** (82)	Screening, diagnosis, and measurement of the severity of depressive symptoms, and where validated, screen for depression.	9	•		•	•	•
Pictogram Suffering Scale (83)	**Cameroon** (83)	Maternal psychosocial distress and suffering as depicted by a picture of person carrying rock on shoulder of increasing size	NR					
Pitt Inventory (84)	**South Africa** (85)	Current clinical experience of depressive illness and special anxieties of childbearing women including sleep, irritability, hypochondriasis, appetite, depression, cognition, libido, retardation, guilt, anxiety, depersonalization, and dependency.	24				•	•
Psychological Satisfaction Scale (86)	**Uganda** (86)	Maternal psychological wellbeing via self-rating of level of satisfaction for life overall, food, housing, income, health, work, perceived safety, freedom from physical violence, education, dignity, ability to help others, and freedom to express spiritual beliefs.	16	•			•	•
Schedule for Clinical Assessment in Neuropsychiatry (SCAN) (87)	**Pakistan** (88)	Identification and classification of mental disorders.	NR				•	•

Measure	Countries where measure use	Measure description	Items	Adaptation	Cog. Interviews	Pretesting	Validity	Reliability
Structured Clinical Interview for the DSM-IV Axis I Disorders (SCID-I) (89,90)	**India** (91); **Nigeria** (92); **Pakistan** (65); **Vietnam** (93)	Screening women for depression using criteria for a DSM-IV major depressive episode.	NR	•			•	•
Self-Report Questionnaire (SRQ-20) (94)	**Bangladesh** (95–98); **Brazil** (97,99); **Burkina Faso** (100); **Ethiopia** (96,98,101–105); **Ghana** (100); **India** (101,102,104,106); **Malawi** (100,107–109); **Nigeria** (110); **Pakistan** (26,88,111); **Peru** (26,101,102,104,112); **South Africa** (113); **Vietnam** (96,98,101,102,104)	Self-reported measure to detect non-specific psychological distress with subscales pertaining to depression/anxiety, somatic symptoms, reduced vital energy, and depressive thoughts.	20	•		•	•	•
Zung Self-Rating Depression Scale (ZSDS) (114)	**China** (115)	Depressive symptoms and depression screening.	20				•	
**Healthy Stress Levels (n=9)**								
Caregiver Feeding Stress (95)	**Bangladesh** (95)	Caregivers rate their feelings regarding their child's feeding difficulties and recall the amount of time spent feeding.	NR					•
Distress Scale (116)	**Vanuatu** (116)	Symptoms of anxiety, depression, nervousness, and stress over the previous week. Meaning of score not stated. Adapted from the Kessler-10 Distress Scale and the CES-D Scale.	15					
Maternal Stress Questionnaire (117)	**Iran** (117)	Maternal stress related to feeling anger, worry, anxiety, quality of sleep, feeling control over one's life, eating on the run, being easily irritated, losing one's temper, feeling overwhelmed.	7					
Parenting Stress Index Short Form (118)	**South Africa** (62)	Areas of stress in the parent-child relationship over the past 12 months: child characteristics, parent characteristics, and stress stemming from situational or demographic conditions.	36				•	•
PedsQL Family Impact Module (22)	**Ghana** (24)	Subscale used to assess parent's self-reported worry	NR	•	•		•	•
Perceived Stress Scale (119)	**Burkina Faso** (100); **Ghana** (100);	Perceptions of stressful feelings and thoughts during the last month	10-14				•	

Measure	Countries where measure use	Measure description	Items	Adaptation	Cog. Interviews	Pretesting	Validity	Reliability
	**Malawi** (100); **Mexico** (120)							
Self-Report Questionnaire (SRQ-20) (94)	**Burkina Faso** (121)	Non-specific psychological distress; subscales include anxiety/depression, somatic symptoms, reduced vital energy and depressive thoughts.	20					
Stressors Questionnaire (10)	**Jamaica** (10)	Issues commonly faced by low-income mothers including economic stress, partner stress, domestic violence, and community violence.	9				•	•
**Equitable Gender Attitudes (n=15)**								
DHS Questionnaire, Women's Empowerment Module (122)	**Ghana** (123)**; Lao PDR** (15)**; Malawi** (124)**; Myanmar** (125); **Nigeria** (126); **Uganda** (127); **28 countries globally**[1] (128); **5 countries in sub-Saharan Africa**[2] (129); **10 countries in sub-Saharan Africa**[3] (130)	Assessment of attitudes about reasons that justify wife-beating (i.e. burning food, arguing with partner, going out without telling partner, neglecting the children, and refusing sexual intercourse)	5	•		•	•	
MICS Questionnaire (131)	**Lao PDR** (132)	Agreement with specific reasons that justify wife-beating.	5			•		
National Family Health Survey-2 (133)	**India** (134)	Reasons given that justify a husband beating his wife.	6			•		
National Family Health Survey-3 (135)	**India** (136)	Caregiver's justification of domestic violence.	5			•		
Gender Norm Attitudes Scale (137)	**Zimbabwe** (6,7)	Two dimensions related to gender norms: (1) concern with promoting equity for women and girls, and (2) belief in the maintenance of men's rights and privileges.	6	•	•	•		•

[1] Bangladesh, Burkina Faso, Benin, Bolivia, Cambodia, Cameroon, Colombia, Dominican Republic, Egypt, Ethiopia, Ghana, Haiti, Jordan, Kenya, Lesotho, Madagascar, Mali, Malawi, Mozambique, Nigeria, Namibia, Nepal, Rwanda, Senegal, Tanzania, Uganda, Zambia, Zimbabwe.

[2] Ethiopia, Kenya, Rwanda, Tanzania, and Uganda

[3] Benin, Burkina Faso, Ethiopia, Mali, Niger, Nigeria, Rwanda, Sierra Leone, Uganda and Zimbabwe

Measure	Countries where measure use	Measure description	Items	Adaptation	Cog. Interviews	Pretesting	Validity	Reliability
Perception of Equality Score (138)	**Zambia** (74)	Perceptions of gender equality.	6					
**Safety & Security (n=26)**								
Conflict Tactic Scale (139)	**Bangladesh** (140,141)**; Brazil** (61)	Intimate partner violence including physical and sexual violence. Experiencing physical violence includes a wife being beaten if she went out without telling her partner, neglected the children or argued with her partner. Experiencing sexual violence was identified based on a women's response to whether she had ever been physically forced to have sexual intercourse when she did not want to.	80	•				
Domestic Violence Module of DHS Questionnaire (122)	**Bangladesh** (142); **Ethiopia** (143); **Liberia** (144)**; Rwanda** (70)**; Tanzania** (58,82,145); **28 countries globally**[4] (146); **5 countries globally**[5] (147)	Exposure to physical and/or sexual violence and experience seeking help.	23	•		•		
Domestic Violence (148)	**South Africa** (55)	Domestic violence as assessed by whether women were slapped or had anything thrown at them (y/n); were pushed or shoved (y/n); were punched with a fist or another object (y/n); or were attacked with a weapon by their partner (y/n).	4	•				
Domestic Violence Measure (149)	**Pakistan** (149)	Domestic violence as measured by whether a woman has faced violence from husbands/family elders.	1			•		
Domestic Violence Sub-scale (10)	**Jamaica** (10)	Domestic violence as it pertains to violence in the community, yard, and household as well as food security, insufficient income, and stressful interpersonal relationships.	9				•	•
Family Violence Against Women (150)	**Bangladesh** (150)	Exposure to family violence assessed via women's self-reported exposure to physical, sexual, or emotional violence including a high level of controlling behavior. Short modified version of WHO collaborative study questionnaire based on the Conflict Tactic Scale (151,152).	NR	•				
Hurt, Insult, Threaten and	**Ethiopia** (77)	Intimate partner violence screening	5					

[4] Azerbaijan, Bangladesh, Bolivia, Burkina Faso, Cambodia, Cameroon, Colombia, Dominican Republic, Gabon, Ghana, Haiti, Honduras, India, Kenya, Liberia, Malawi, Mali, Mozambique, Nepal, Nigeria, Peru, Republic of Moldova, Rwanda, Sao Tome and Principe, Timor-Leste, Uganda, United Republic of Tanzania, Zambia, Zimbabwe.

[5] Egypt, Honduras, Kenya, Malawi, Rwanda.

Measure	Countries where measure use	Measure description	Items	Adaptation	Cog. Interviews	Pretesting	Validity	Reliability
Scream (HITS) Scale (153)								
Intimate Partner Violence (154)	**India** (154)	Maternal exposure to physical violence in the two years preceding the survey.	NR					
National Family Health Survey-2 (133)	**India** (155)	Women's self-reported physical abuse by a family member more than once in the previous 12 mo, once in the previous 12 mo, since age 15 years but not in the previous 12 mo, or never since age 15 years.	3			•		
National Family Health Survey-3 (135)	**India** (156,157)	Women's experience with emotional violence.	3			•		
National Family Health Survey-4 (158)	**India** (159,160)	Lifetime prevalence of three types of violence by male partners: less severe violence, severe physical violence, and sexual violence.	17			•		
Prevalence of Domestic Violence (161)	**India** (161)	Mother's experience of psychological abuse and sexual coercion.	5			•		•
Psychological Wellbeing Scale (86)	**Uganda** (86)	Mothers' satisfaction across several attributes including perceived safety and freedom from physical violence. Adapted from Organisation for Economic Cooperation and Development Guidelines (162).	16	•				
WHO's Violence Against Women Instrument (VAWI) (163)	**Togo** (164)	Women's experience with controlling behavior, emotional violence, or physical violence from their partner over the past 12 months.	16					
**Social Support (n=54)**								
**Social Capital**								
Group membership (112)	**Peru** (112)	Membership in groups related to work or trade, religion, community association, cooperatives for saving, gender, communal kitchens, mothering, sports, irrigation, security, or politics.	N/A					
Maternal Social Capital and Grandmaternal Childcare Support (120)	**Mexico** (120)	Maternal support based on who mothers would turn to for advice and support in certain situations, relatives and friends with whom mothers had a close relationship, support from social groups, and support from a grandmother.	N/A					
Religious affiliation (165)	**Mozambique** (165)	Women who said that they belonged to a church versus women who said that they did not belong any church; affiliation with any organized religion in the previous 2 weeks.	N/A					
Short Adapted Social Capital Assessment Tool (166)	**Ethiopia** (102,167)**;** **India** (102); **Peru** (102); **Philippines** (168)**; Vietnam** (102)	Operationalized social capital as structural social capital ('connectedness') and cognitive social capital ('reciprocity, sharing, trust).	18	•				

Measure	Countries where measure use	Measure description	Items	Adaptation	Cog. Interviews	Pretesting	Validity	Reliability
Social Capital (169)	**Myanmar** (170)	Details not stated.	9	•		•	•	•
Social Capital (138)	**Zambia** (138)	Part of a women's empowerment measure.	9					
Social Participation (171)	**Nepal** (171)	Participation in groups related to agriculture, water use, land/forest use, credit or microfinance, insurance, trade or business, civics, religion, mother's group or other women's group.	N/A	•		•		
Trust in religious leaders and institutions (172)	**Philippines** (172)	Trust in religious leaders and institutions.	1					
**Social Network**								
Composition of Mothers' Social Networks (173)	**India** (173)	Mothers' social networks within and outside their household (comprised of the six individuals a mother talks to the most), value was a composite of network size, literacy, sex, % of network members living outside of the household, % of members not related to mother, and presence of mother/MIL/husband in network.	N/A					
In-camp network size (174)	**Uganda** (174)	Number of households from respondent's pre-displacement farmers' group living in their current administrative block in the camp. Local social network has two components: long-standing ties and daily contact.	N/A					
Kin Network (175)	**Tanzania** (175)	Kin network was defined as the number of full brothers and number of sisters a mother had living in the study area. Mother's place of birth was a proxy for kin support network.	N/A					
Personal Network (176)	**Mexico** (176)	Mothers asked to name 4 alters or people that they know, how close they were to each alter, and whether they had ever discussed infant and young child feeding practices with each other. Mothers reported on each alter's sex, age, number of children, level of education and ability to speak an indigenous language.	N/A					
Social Network (60)	**Brazil** (60)	Number of friends and relatives participant feels comfortable with and can talk to about almost anything.	N/A					
Social Network (177)	**Mexico** (177)	Type of networks (within or outside the family), size of networks, frequency of interactions and type of support (economic, childcare, etc.)	N/A					
Social Network (62)	**South Africa** (62)	If they received a child support grant and the size of a mother's social network, calculated as the number of close friends and relatives multiplied by the frequency of contact in the past month.	N/A					
Social Network (178)	**Bangladesh** (178)	Mothers' knowledge of other mothers who had adopted optimal infant and young child feeding practices in the community, and any personal connection to those mothers.	N/A					
Social Network/Support Network (179)	**Brazil** (61)	Number of friends and relatives with whom the woman could rely on in the last 12 months.	N/A					
**Social Support**								
Adequacy of Social Support (43)	**Tanzania** (180)	Types of help women received from their husbands by asking if partners had participated in each of 6 tasks that men rarely do, as identified in an iterative pile-sort formative research (181).	6	•				

Measure	Countries where measure use	Measure description	Items	Adaptation	Cog. Interviews	Pretesting	Validity	Reliability
Breastfeeding Support (182)	**Vietnam** (182)	Received breastfeeding support from a health worker (e.g., nurse, doctor, or village health worker) and from a family member, relative, or friend during pregnancy or within 3 days after birth.	2					
Community Membership (183) and Community Cohesion (184)	**India** (183)	Community membership (asked the head of the household if he/she is a member of any community groups) and community cohesion (used community cohesion questionnaire from (184).	9					
Duke UNC Functional Social Support Scale (185)	**Nicaragua** (186); **Tanzania** (72,187); **Uganda** (86)	Three types of social support: confidant (having someone to talk with about important topics), affective (being shown love and caring) and instrumental (such as financial help during an emergency).	10-15	•			•	•
Family Support Scale (188)	**Indonesia** (188)	Family support in terms of emotional, material, and informational support provided by families in selecting complementary feeding.	13				•	•
Household Bargaining Power Sub-scale (189)	**Nepal** (189)	Respondent reported being an active group member in groups related to agriculture, water use, land/forest use, credit or microfinance, mutual help or insurance, trade or business, civics, religion, or other women's groups.	9					
Male Involvement in Housework (190)	**Benin** (190)	Whether women get help from adult males in their household to prepare meals, clean, do laundry, make purchases, take care of kids. A nonfamily groups' domain included participating in an environment for creating a sense of solidarity with other women.	2					
Maternity Social Support Scale (MSSS) (191)	**Ethiopia** (77)	Social factors associated with postpartum depression (i.e. lack of family support, lack of partner support, conflict with partner, feeling controlled by or unloved by partner, low friend network).	6					
Medical Outcomes Study (MOS) Social Support Survey (192)	**Jamaica** (10)	Tangible and emotional support.	4				•	•
Multi-Dimensional Scale for Perceived Social Support (MSPSS) (193)	**Pakistan** (26,65)	Perceived social support including support from family, friends, and significant other.	12	•				
Non-Maternal Adult Female Household Members (194)	**Ethiopia** (194)	Number of non-maternal adult female household members; specifically, the number of non-maternal AFHMs relative to the number of under-5 children.	N/A					

Measure	Countries where measure use	Measure description	Items	Adaptation	Cog. Interviews	Pretesting	Validity	Reliability
Oslo Social Support Scale (OSSS-3) (195)	**Pakistan** (20)	Relationships with and support from family, friends, and neighbors.	3				•	•
Paternal Involvement in Childcare (196)	**Vietnam** (196)	Time fathers spent involved in 5 child-care activities over the previous week: feeding, supporting his wife with child feeding, playing with child, putting child to bed, and bathing child. Providing health care defined by 2 variables: caring for a sick child and bringing the child for immunizations. Paternal involvement was the amount of time fathers spent during the previous week doing household chores and caring for older children.	9				•	•
Perceived Social Support (197)	**Nigeria** (197)	The degree to which six types of family members or friends (respondent's mother, husband's mother, husband, her sisters, other women in extended family, and her friends) would support their feeding method.	6	•				
Perceived Social Support (5)	**Zimbabwe** (6,7)	Four functional social support domains (tangible, emotional/informational, affectionate, and positive social interaction) and a question about the number of close family and friends. Adapted from Interpersonal Support Evaluation List (198) and the Medical Outcomes Study Social Support Survey (192).	16	•	•	•		•
Social Support (38)	**Brazil** (38)	Combined items from the Medical Outcomes Study Social Support Survey (179) and questions to assess material, practical, relationship, and emotional support (199).	20	•			•	
Social Support (78)	**Kenya** (78)	Social support from others (yes/no).	1					
Social Support (127)	**Uganda** (127)	Marital status as an indicator of social support and media access as an indicator for informational support.	2					
Social Support (200)	**Brazil** (200)	Three dimensions of social support, social interaction, presumed support, and received support.	11					
Social Support (73)	**Chad** (73)	Assistance for caregivers in accomplishing daily activities in addition to the age of the father, household size, dependency ratio, help available for usual tasks, family structure, and ethnic group.	NR					
Social Support Index (201)	**Kenya** (201)	Support received or provided by the father or grandmother related to household activities, childcare, emotional support, and financial support. Based on formative research.	12					
Social Support Measurement (106)	**India** (106)	Economic or emotional help or assistance from groups or leaders related to work/trade, politics, religion, credit or funerals, sports, family, friends, government/civil service, charitable organizations/NGOs in the last 12 mo.	NR					
Social Support Resources (202)	**Jamaica** (202)	Availability and use of child care, partner presence in the household, financial or service contributions from household members, community residence, proximity to family members, formal sources of support.			•			
Social Support System Adequacy (200)	**Myanmar** (203)	Not stated.	NR	•				
Social Support Scale (204)	**South Africa** (205)	Number of close contacts, frequency of contact with friends/family, and frequency of practical support.	3					

Measure	Countries where measure use	Measure description	Items	Adaptation	Cog. Interviews	Pretesting	Validity	Reliability
Sources of Breastfeeding Support (206)	**Brazil** (206)	Not stated.	NR					
Support from Husband (74)	**India** (74)	Social support obtained from the husband in helping to care for the child and in-home tasks	NR			•		
Support in Household Chores and Perceived Instrumental Support (96)	**Bangladesh** (96); **Ethiopia** (96); **Vietnam** (96)	Support with household chores and perceived instrumental support identified as help with accommodation, money, and food.	11-12					
Women's Perceived Social Networks (18)	**Mali** (18)	Perceived social networks based on material, practical, cognitive, and emotional support including mother-in-law support, husband support, household cooperation, natal kin network, and total network size. Based on formative research.	NR					
**Time Sufficiency (n=17)**								
Caregiver Workload (73)	**Chad** (73)	Mean, minimum, and maximum hours spent on domestic tasks, agricultural work, handicrafts, drying vegetables, leisure, and total hours worked/day over the past 6 months.	N/A			•		
Income Related Work Hours (207)	**Nepal** (207)	Employment status (formal, informal, domestic) and number of income-related work hours per week	2			•		
Leisure Time/Day (74)	**India** (74)	Mother's workload was assessed by the amount of leisure time available per day.	N/A			•		
Maternal Time Use (208)	**Mali** (208)	Observation of daily amount of time spent on categories of activity: productive workload or maternal income-generating activities, domestic work, child-care activities, social activities, and miscellaneous activities.	N/A					
Maternal Work Patterns (202)	**Jamaica** (202)	Hours and days of work per week (income-generating activities, maternal food production, childcare) as well as distance and time from home to workplace. Based on formative research.	N/A					
Number of Days Spent Farming (209)	**Tanzania** (209)	Time mothers spent in agricultural activities and in cultivation of various crops during the last main agricultural season.		•		•		
Perceived Time Stress (5)	**Zimbabwe** (6,7)	Perceptions of time stress, workload, and time available for caregiving.	6		•	•		•
Time Allocation Module from Women's Empowerment in Agriculture Index (WEAI) (210)	**Bangladesh** (211); **Nepal** (171,189,212,213); **Tanzania** (43);	Time allocation module (1 of 5 domains in WEAI) includes 2 indicators: workload and leisure. (1) Workload estimated as sum of total time spent on domestic work, care for children and elders, wage work or employment and subsistence activities such as farming and livestock and schoolwork, based on 24-hour recall of all activities undertaken the day before. (2) Subjective satisfaction with time available for leisure is assessed with a single item.	N/A					

Measure	Countries where measure use	Measure description	Items	Adaptation	Cog. Interviews	Pretesting	Validity	Reliability
	**Zambia** (138); **5 countries**[6] (214)							
Women's Time Use (215)	**Burkina Faso** (215)	Self-reported estimates of average time spent on agricultural activities (planting, weeding, and harvesting) and average time spent on other activities (livestock, agriculture, hunting/gathering, and child care) and domestic activities during the 7 days prior to the survey.	N/A					

N/A: not applicable
NR: not reported

[6] Bangladesh, Nepal, Cambodia, Ghana, Mozambique

Table S4: Caregiver Resource measures and findings in complementary feeding and responsive feeding papers (N=163)
Presented alphabetically by measure (pre-existing followed by author-developed) then author of included paper

CR term used by authors	Location of included paper	Measure (citation)	Study Design	Sample size and description of population where measure implemented	Adaptation, translation, cross-cultural equivalency, or pretesting	Caregiver Resource Specific Findings
**Self-Efficacy**						
Maternal Self-Efficacy for Complementary Feeding (MSE-CF)	Bangladesh (1)	Author-developed (1)	Cross-sectional	n=457 rural mothers of children 6-24 months of age.	Questions translated from English to Bengali. Cognitive interviewing conducted with 15 mothers not in the survey sample.	"Moderated mediation." Intervention impact mediated by increased MSE-CF in the case of feeding green leafy vegetables in the last 24h; mothers with higher MSE-CF gained the most from the intervention on this behavioral outcome. Intervention did not work through increased MSE-CF to effect timely introduction of egg; MSE-CF did not potentiate the effect on timely introduction of egg.
Maternal Self-Efficacy	Iran (4)	Maternal Self-Efficacy Scale (2)	Cross-sectional	n=423 urban and rural mothers of children <2 years old. Mean maternal age: 27.4 +/-5 years, range 17-40; Mean infant age: 15.1 +/- 5.8 months.	Adapted with reference to Lakshman et al. 2021 (216). Based on the score, maternal self-efficacy was categorized into four levels: very good (30–40), good (20–30), poor (10–20) and very poor (<10). Questionnaire translated into Farsi. Pilot study to test questionnaire yielded no major changes.	Converse association with increased use of pressure style. Significant association with maternal feeding styles, including control of home food access, pressure to eat, restriction for weight control, restriction for health, encouragement and modeling.
Maternal/Parenting Self-Efficacy	Brazil (3)	Maternal Self-Efficacy Scale (2)	Cross-sectional	n=595 low-income urban mothers of children 6-24 months of age.	High and low categories defined as above and below the median of the 10 summed items; scores were based on the average of the items answered.	Low maternal self-efficacy was not associated with short stature, nor did it mediate or modify the relationship between depressive symptoms and short stature. Underweight was not associated with maternal self-efficacy.
Mothering Self-Efficacy	Zimbabwe (6)	Mothering Self-Efficacy (5)	2x2 cluster randomized community-based trial	n=3181 mother-infant pairs; recruited at pregnancy and enrolled in SHINE trial, followed through 18mo in rural Zimbabwe.	Not stated.	No significant relationship was observed between mothering self-efficacy and child diet diversity (OR 1.07 (0.90-1.28); p = 0.424).
Mothering Self-Efficacy	Zimbabwe (7)	Mothering Self-Efficacy (5)	2x2 cluster randomized community-based trial	n=4025 mothers (measured growth in 4,073 children); recruited at pregnancy and enrolled in SHINE trial, followed through 18mo in rural Zimbabwe.	Not stated.	No significant relationship was observed between mothering self-efficacy and child LAZ at 18 months (OR 0.93 (0.78, 1.09), p=0.36).
Parenting Self-Esteem, including perceived self-efficacy and satisfaction from parenting	Jamaica (10)	Author-developed (10)	Case control study	Mothers of undernourished IYC (n=139 cases) and adequately nourished IYC (n=71 controls) 9-30mo old; recruited from urban government health centers.	Questionnaire was pretested with 20 non-study mothers on two occasions, two weeks apart.	Mothers of undernourished children had lower parenting self-esteem (p<0.01).

CR term used by authors	Location of included paper	Measure (citation)	Study Design	Sample size and description of population where measure implemented	Adaptation, translation, cross-cultural equivalency, or pretesting	Caregiver Resource Specific Findings
Perceived Behavioral Control (PBC)	Kenya (12)	Perceived Behavioral Control (11)	Cross-sectional	n=665 female caregivers of children <2 years old (n=202 mothers of children 6-23mo), received nutrition education from SUSTAIN.	Adapted to IYCF. Pretested on 60 respondents; checked for comprehension, clarity, and cultural appropriateness and ease of administration of the scales.	PBC had a significant effect on a number of IYCF practices, such that one unit increase in PBC index increased the expected number of IYCF practices by 33%. However, PBC did not have a significant effect on use of IYCF practices for mothers of children 6-23 mo alone.
Perceived Self-Efficacy	Burkina Faso (13)	Author-developed (13)	Cross-sectional	n=1889 mother-infant pairs 6-23 months old in rural villages. Average maternal age 28 years old.	Not Stated.	Among mothers who introduced soft, semi-solid, and/or solid foods, 14.6% (CI 12.2, 17.4) felt capable giving meat, fish or egg every day to child; dark green leafy vegetables 62.9% (CI 57.9, 67.6); carrot, squash, sweet potato 11.2% (CI 9.0, 14.0).
Self-Efficacy	Iran (14)	Author-developed (14)	Quasi-experimental intervention	n=170 mothers of 6-24-month-old children, in an urban area, mean age 30 +/- 5.04 years old; range 18-49 years. Children mean age 14.11 +/- 4.94.	Review of literature and related tools. Two focus groups discussions with mothers of 6 to 24-month-old children (n=7). Pilot test on 36 participants.	Intervention improved self-efficacy of mothers.
Self-Efficacy	Lao PDR (15)	Author-developed (15)	Cross-sectional	n=100 mothers (17-46 years of age) of children 0-4 years of age in a semi-urban area. (n=23 0-11 months; n=17 12-23 months).	Not Stated. Sample question: “How easy do you find it to access health services when you need to?”; “How easy do you find it to borrow money when you need to?”.	Mean self-efficacy score for health services was significantly lower (p= 0.067) for mothers with stunted children than for those without stunted children. No difference between groups in mean self-efficacy score for borrowing money.
Self-Efficacy	Iran (16)	Author-developed (16)	3-arm cluster randomized controlled trial	n=90 urban mothers of full-term singleton, exclusively breastfed infants 4 mo to 4 mo and 29 days old. Infants average age 130.08 days ¬± 7.48. Mean age of mothers 29.87 (years) ¬± 4.68. 30 mothers in each arm.	Changes made based on the opinions of health education experts, guidelines on complementary feeding by Iranian Ministry of Health. Example item: “I can prepare a food suitable to the age of my infant.”	Gain-framed and loss-framed messages equally increased self-efficacy in observing appropriate complementary feeding. Both experimental groups were similar in terms of self-efficacy score, but loss-framed messages increased attitude more. Cannot conclude that effect of message framing type is always directly related to higher self-efficacy.
Self-Efficacy in Infant Care Scale (SICS)	Thailand (17)	Author-developed (17)	Cross-sectional sample	n=397 mothers 16-47 years old (mean 26.8 +/- 6.3 y) of infants <12 mo old who attended well-baby clinics for immunizations in urban area. 82% self-identified as primary caregiver, 60% first-time mothers, education level ranged from primary school to graduate school (mean education = 10 ¬± 3.6 years); 54% were employed.	Individual and focus group interviews with 20 mothers (pregnant, or with infants 1-3, 4-6, 7-9, and 10-12 months old) used to develop tasks, competencies, barriers, and challenges for measure. Also, literature, previous work on similar measures, and authors' clinical experience. Used Bandura's guide for constructing a self-efficacy scale (217).	Factor analysis with 67 items of five expected factors was carried out, resulting in a five-factor solution with 42 items and explained 53% of the variance. Diet was split into two factors: the first factor (diet 1), consisting of six items, was related to bottle- feeding and solid food, and the other factor (diet 2), consisting of four items, was related to breast-feeding, burping, and position during feeding. The 42-item SICS was composed of five factors: developmental promotion (14 items), general health care (13 items), safety (5 items), diet 1 (6 items), and diet 2 (4 items).

CR term used by authors	Location of included paper	Measure (citation)	Study Design	Sample size and description of population where measure implemented	Adaptation, translation, cross-cultural equivalency, or pretesting	Caregiver Resource Specific Findings
Social Power	Mali (18)	Author-developed (18)	Cross-sectional	n=261 Fulbe mothers of children <5 years of age in rural Mali.	Not Stated.	Mothers with a higher score in passivity/helplessness were more likely to have wasted or stunted children (controlling for confounders). Mothers who scored better in felt control were more likely to have a WHZ in the normal range.
**Perceived Physical Health**						
Health-Related Quality of Life	Pakistan (20)	Euro-Qol (EQ 5D) (19)	RCT	n=107 mothers of children <= 30mo (54 intervention, 53 control), at outpatient pediatric departments of participating hospitals.	Translated to Urdu (218)	Scores for health-related quality of life improved significantly for the intervention group as compared to the control group.
Perceived Health Status	Zimbabwe (6)	RAND 36-item Health Survey (21)	2x2 cluster RCT	n=3181 mother-infant pairs; recruited at pregnancy and enrolled in SHINE trial, followed through 18mo in rural Zimbabwe.	Modified survey items to make them contextually relevant and dropped survey items that could not be modified (5).	No significant association between perceived health status and infants fed diverse diet (OR 0.99 (0.91-1.07); p = 0.856)
Perceived Physical Health	Zimbabwe (7)	RAND 36-item Health Survey (21)	2x2 cluster RCT	n=4025 mothers (measured growth in 4,073 children); recruited at pregnancy and enrolled in SHINE trial, followed through 18mo in rural Zimbabwe.	Not stated.	Mothers who perceived themselves to be in better health compared to mothers who perceived themselves to be in worse health had children with better linear growth at 1 and 12 months of age.
Caregiver and Family Quality of Life (QoL)	Ghana (23)	PedsQL Family Impact Module (22)	Cross-sectional	n=76 caregivers and their children with cerebral palsy (CP) 18mo to 12yo in eight rural communities in Ghana. Majority of children (72%) were under 5yo and had severe cerebral palsy.	Pilot tested and verbally translated into three local languages.	QoL scores were significantly lower among caregivers whose children had the greatest difficulties with feeding (median score 9.0) compared to those with least difficulties (24.6, p=0.004) even with adjustment for potential confounders (caregiver and child age, socio-economic status, north/south, and CP severity). Similar QoL for caregivers with a child with CP who was underweight (10.8) and caregivers of not under-weight child (11.8, p=0.12).
Quality of Life (QoL)	Ghana (24)	PedsQL Family Impact Module (22)	Pre/post evaluation, 11-month participatory training	n=75 caregivers of children with cerebral palsy 18mo to 12yo, at baseline (n=64 caregivers at endline)	Translated to three languages Twi, Gruni, and Kusaal, including forward/ back-translations by linguistic experts. Modified following cognitive field testing in the three sites.	Assessed impact of intervention on QoL and on child nutrition, but did not assess relationship between QoL and child nutrition. All domains of QOL (including physical functioning) improved significantly baseline to endline.
Caregivers' Health-Related Quality of Life	Pakistan (26)	WHO Quality of Life- Brief version (WHOQoL) (25)	Two-arm, single-blinded, individual RCT	Pregnant women in third trimester with increased risk of postnatal depression.	n/a [study protocol]	n/a [study protocol]
**Mental Health**						

CR term used by authors	Location of included paper	Measure (citation)	Study Design	Sample size and description of population where measure implemented	Adaptation, translation, cross-cultural equivalency, or pretesting	Caregiver Resource Specific Findings
Maternal Depressive Symptoms	Pakistan (28)	Aga Khan University Anxiety and Depression Scale (27)	Cross Sectional	n=325 mothers (18-44 years old) with children <2 years old; recruited from urban and rural districts.	n/a	Maternal depressive symptoms are related to higher risks of child underweight status.
Depressive Symptoms (Maternal Depression)	Brazil (30)	Beck Depression Inventory (29)	Cohort study	n=360 mothers with infants 0-12m; recruited from Sao Leopoldo Hospital.	n/a	After adjustment for confounders, moderate and severe maternal depression was significantly associated with breastfeeding cessation.
Maternal Depression/Depression Symptoms	Malaysia (32)	Beck Depression Inventory Second Edition (BDI II) (31)	Case control study	n=124 mothers with children 6mo to 5yo (62 malnourished children; 62 well-nourished children from eight governmental health clinics in Kuala Langat district.	Used Malay translated version (219).	Current maternal depression was a significant risk factor to malnutrition. Mothers who experienced depression were 2.2 times more likely to have malnourished children (OR 2.2, 95% CI 1.03, 4.61), according to binary logistic regression.
Depressive Symptoms (Maternal Depression)	Bangladesh (35)	Center for Epidemiologic Studies Depression Scale (CES-D) (33)	Stratified cluster field trial	n=463 mothers of IYC 4-14mo in rural and peri-urban areas; recruited from Save the Children and randomized into 10-month parenting intervention or standard care.	Conducted preliminary interviews and modified several items.	Depressive symptoms were significantly reduced among women who met as a group twice monthly compared to those who had mostly home or clinic visits (p=0.004). Mean scores among intervention group were 13.28 (SD 14.3) vs 18.07 (SD 15.5) in standard care. Depressive symptoms were not measured in relation to IYCF/child nutrition, though nutrition indicators were collected as outcome measures of the intervention.
Maternal Depression	Jamaica (10)	CES-D (33)	Case control study	n=210 mothers of IYC 9-30mo. Participants were recruited from government health centres in parishes and urban areas.	After piloting, the wording of the questions was adapted to be more culturally appropriate and several of the questions were omitted.	Mothers of undernourished children reported more depressed symptoms (p<0.01).
Maternal Depressive Symptoms	Bangladesh (36)	CES-D (33)	Cohort study	n=221 mothers of IYC 6-12mo. Participants were recruited from 6-mo double-blinded RCT	Clinical cutoff of 16 was used in descriptive analyses, but because the cutoff has not been validated in Bangladesh, CES-D was used as a continuous variable to test hypotheses	No differences in infants' LAZ or WLZ at 6mo based on mothers' depression status at 12 mo. At 12 mo, significant depression-by-time effect on length-for-age, which indicated worse growth for infants of mothers with depressive symptoms. Correlation matrix showed that maternal depressive symptoms were significantly related to length-for-age at 12 mo (r = -0.13, p = 0.05), but not to other anthropometric indexes at 6 or 12 mo
Maternal Depression	Ethiopia (39)	CES-D (33)	Cluster RCT	n=1220 pregnant mothers and their offspring; Women were recruited starting at 2-3 months gestation. Child	n/a	There was no difference between the intervention and control children on any anthropometric indicator.

CR term used by authors	Location of included paper	Measure (citation)	Study Design	Sample size and description of population where measure implemented	Adaptation, translation, cross-cultural equivalency, or pretesting	Caregiver Resource Specific Findings
				data was collected 2 to 13 months.		
Maternal Depressive Symptoms	Bangladesh (37)	CES-D (33)	Community-based randomized trial	n=507 mothers of severely underweight (WAZ < -3) IYC 6-24mo, who had recovered from an acute infection at Dhaka hospital of icddrb.	Adapted after piloting: two questions from the CES-D scale were omitted because the questions were not understood and added five re-worded questions were added.	There was no significant relationship between maternal depressive symptoms and the nutritional status of the children
Maternal Depressive Symptoms	India (42)	CES-D (33)	Cross-sectional	n=2930 mothers and their IYC 6-48mo in rural India	Adapted through the use of flexible, local cut-offs and a binary indicator to capture heterogeneity. Translated into Hindi; back-translation and pilot testing.	Children of mothers with high MDS also had poorer dietary diversity. Children of mothers with high MDS had a higher rate of stunting and under-weight than their counterparts, with the odds ~2 times in models adjusted for child age and sex, and ~1.4 to 1.5 times in fully adjusted models. There was no significant association between maternal MDS and wasting.
Risk of Probable Depression	Tanzania (43)	CES-D (33)	Cluster-randomized effectiveness trial	n=Women in 591 food insecure households as defined by the community and had a child aged <1 y.	Used a cutoff value that has been validated for use among similar populations in East Africa.	At baseline, 41.2% of women were experiencing probable depression; the intervention reduced this by11.6pp (P=0.04).
Maternal Depression	Brazil (38)	CES-D (33)	Cross-sectional study	n=595 mothers of children 6-24mo; recruited from four low-income rural areas.	Used as a dichotomous variable with cut-off of 16 to indicate depressive symptoms. Developed a Portuguese-language version through translation and back-translation.	No significant association between maternal depression and HAZ.
Maternal Depressive Symptoms	Brazil (3)	CES-D (33)	Randomized Control Trial	n=660 Mothers; pregnant women aged >18 were selected from this urban region to participate in the trial	n/a	Maternal depression negatively modified impacts of the intervention on child development
Depression Status	Ghana (40)	CES-D ((33)	Cross Sectional	n=384 caregivers of IYC <5yo seeking CWC services at health center or its 2 outreach posts.	Translated and administered in Akan or Dagbani language.	In adjusted model, the risk of stunting to children of depressed mothers was statistically significant (AOR = 2.48, 95 % CI 1.29, 4.77, p= 0.0011).
Maternal Depressive Symptoms	Ghana (41)	CES-D (33)	Cross Sectional	n=595 low-income mothers with children 6-24 m were recruited from an urban area	n/a	Maternal depressive symptoms correlated with higher odds of stunting amongst children but were not related to children's underweight status
Depressive Symptoms	St. Lucia, Antigua, and Jamaica (34)	CES-D (33)	Prospective cohort study using data from cluster	n= 601 mother-child pairs; Recruited at 6 week postnatal clinics in Jamaica (n=396) and St.Lucia	Previously piloted and adapted for use in the Jamaican setting.	Maternal depressive symptoms were associated with higher scores on uninvolved, restrictive and forceful feeding styles and lower responsive feeding.

CR term used by authors	Location of included paper	Measure (citation)	Study Design	Sample size and description of population where measure implemented	Adaptation, translation, cross-cultural equivalency, or pretesting	Caregiver Resource Specific Findings
			randomized parenting trial	(n=103), and at 8-10 weeks in Antigua (n=102). Feeding data collected at 12mo.		
Parental mental health	Vietnam (45)	Everyday Feelings Questionnaire (EFQ) (44)	Cohort study	n=2,000 Children and caregivers, 96% of whom were mothers; mix of urban/ rural areas	n/a	Poor parental mental health is positively correlated with child underweight status.
Postpartum Depression (PPD)	India (52)	Edinburgh Postnatal Depression Scale (EPDS) (46)	Prospective cohort study	n=287 mothers and their infants in urban area	Translated, pilot tested in Gujarati language, and adapted.	Mean feeding index score was lower in depressed than non-depressed (6.72¬±1.22 vs 7.92¬±0.6, p<0.001) mothers, which was significantly different. Infants of depressed mothers were more in risk of malnutrition compared to non-depressed mothers (32.0% vs 12.7%). Mean MUAC values of the infant were significantly low in depressed mothers (13.70¬±0.43) than non-depressed (13.93¬±0.38)
Postpartum Depression (PPD)	Ethiopia (50)	EPDS (46)	Cohort Study	n=458 Mother Child pairs; Adolescent (mean age 16) and adult (mean age 26) mothers were recruited from rural areas after giving birth in hospital or clinic	n/a	Did not examine child growth outcomes in relation to mental health status
Maternal depressive symptoms	South Africa (54)	EPDS (46)	Intervention trial (cluster randomized)	N=1,111 pregnant women	Adapted; used short and ultrashort versions of EPDS (220)	Pattern of maternal depressed mood was not associated with HAZ or being stunted (HAZ <-2 SD), but was significantly associated with WAZ (F =3.9, p =0.01). Children of never depressed mothers had higher WAZ scores than children of mothers with recurrent episodes of depressed mood (p =0.01; ES =-0.07). Being malnourished significantly varied based on maternal depressed mood (F =3.1, p =0.03). Children of mothers with depression during pregnancy had a lower probability of being malnourished compared to children of mothers with depression post-partum (p =0.02; ES =-0.02).
Postnatal Depression	Tanzania (58)	EPDS (46)	Cohort Study	n=1128 mother child pairs; pregnant women were recruited from two urban health clinics	Locally translated and piloted Swahili version implemented.	Postpartum depression is associated with impaired child growth in the form of significantly lower HAZ scores at 24months.
Maternal Depression	India (53)	EPDS (46)	Cohort study	n= 652 mother-infant pairs; Mothers recruited from a rural region in third trimester of pregnancy; followed with infants up to 6-8m postpartum	n/a	In bivariate analysis, maternal postpartum depressive symptoms predicted infant underweight and antepartum depressive symptoms predicted infant stunting

CR term used by authors	Location of included paper	Measure (citation)	Study Design	Sample size and description of population where measure implemented	Adaptation, translation, cross-cultural equivalency, or pretesting	Caregiver Resource Specific Findings
Maternal Depression	South Africa (55)	EPDS _(46)	Prospective, pragmatic non-randomized two-group cohort study	n=1,310 pregnant women attending ANC at one of four clinics included in the study	n/a	Rates of depressed mood were low (12.6%), as was potential clinical depression (4.4%); similar in the two groups at 12 months. At 12 months and over time, mean depression scores were significantly higher for comparison group compared to intervention, but proportions with depressed mood (EPDS > 13) were similar at 12 months across groups.
Mental Health	Zimbabwe (6)	EPDS (46)	2x2 cluster randomized community-based trial	n=3181 mother-infant pairs; Pregnant women enrolled in SHINE trial and followed through 18 months	Adaptation included modifying survey items to make them contextually relevant or dropping survey items that could not be modified (5).	No significant association between maternal mental health and infant being fed diverse diet observed (OR 0.85 (0.64-1.19); p=0.249).
Maternal depression	Gambia (51)	EPDS _(46)	Cross Sectional	n= 116 postpartum mothers aged 20-25 were recruited from urban and rural regions	Adapted; detail not stated.	Significant association between maternal PPD and child nutritional status.
Maternal depressive symptoms	Bangladesh (47)	EPDS _(46)	Cross Sectional	n=300 Mother child pairs were recruited from attendees of a rural medical college	Piloted in neighboring rural district.	Significant relationship between maternal depression and responsive feeding
Postnatal depression	Bangladesh (48)	EPDS _(46)	Case control study	n=280 mothers including cases (children with WLZ < - 3) and controls (children with WLZ > - 3). All were in ENID trial (Early Nutrition and Immune Development).	Adapted and used the Bangladesh version (221).	Overall prevalence of maternal depressive symptoms was similar between mothers of cases and controls: 13% vs 12%. Maternal depressive symptoms not significantly associated with severe wasting in infants (OR 1.37 (0.32, 6.00), p= 0.67).
Depressed mood	South Africa (56)	EPDS (46)	Cross Sectional	n= 238 Mothers aged 25-34 were recruited from rural areas	n/a	Maternal depression was found to predict child stunting and inappropriate complementary feeding
Mental Health/Depression	Zimbabwe (7)	EPDS _(46)	2x2 cluster randomized community-based trial	n=4,025 mothers; Pregnant women enrolled in SHINE trial and followed through 18 months	Adapted from Matare et al. (2015)(5). Translated and validated in Shona (222).	Children of mothers with fewer depressive symptoms had significantly better linear growth outcomes (LAZ and/or stunting) at 18 months.
Maternal Depressed Mood	South Africa (57)	EPDS _(46)	RCT	n=958 Mother child pairs (including 457 Control and 502 Intervention), recruited as pregnant women at peri-urban community level and followed up 36 months	n/a	Children of mothers with depressed mood were more likely to have low WAZ scores

CR term used by authors	Location of included paper	Measure (citation)	Study Design	Sample size and description of population where measure implemented	Adaptation, translation, cross-cultural equivalency, or pretesting	Caregiver Resource Specific Findings
Depression	China (49)	EPDS (46)	Cohort study	n=201 Mother child pairs	Adapted; detail not stated.	Children of depressed mothers did not meet adequate energy, protein, and carbohydrate intakes
Depression in Mothers and Severity of Depression	Pakistan (20)	EPDS (46)	RCT	n=107 mothers with children up to 30mo, presenting at the outpatient pediatric departments of participating hospitals. 54 included in intervention and 53 in control.	Previously translated into Urdu (218).	EPDS used to screen mothers for depression to be included in intervention.
Maternal Mental Health	Brazil (60)	General Health Questionnaire (GHQ-12) (59)	Cross Sectional	n=238 Mother-infant pairs; 6-month-old infants and their mothers recruited from primary healthcare units in urban hospitals	Measure adapted and piloted in Portuguese.	Poor maternal mental health is positively associated with inadequate child nutritional status at 6 months.
Common Mental Disorders (CMD)/"Suspected Depression"	Brazil (61)	GHQ-12 (59)	Cross-sectional study	n=217 mothers and their children up to 15mo; Data derived from a prospective cohort study that followed the first year of life of newborns attended at four primary care facilities of Rio de Janeiro from June 2005 to December 2009.	Used translated and adapted version in Portuguese.	Significant associations were observed in the bivariate analyses between overall physical violence and non-breastfeeding; in adjusted multivariate analyses, the associations remained significant.
Maternal Depression	South Africa (62)	GHQ-12 (59)	RCT	n=1200 mother child pairs; HIV positive mothers (mean age 26.5y) with infants 0-12m were recruited from rural and urban areas	n/a	Intervention mothers reported a larger decease in depressed mood and their infants reported a larger increase in weight-for-age z scores
Maternal Depression	Pakistan (64)	Hamilton Depression Rating Scale (HDRS) (63)	Cluster-RCT	n=903 pregnant women, married and in third trimester of pregnancy. All women who fulfilled the criteria for a DSM-IV major depressive episode were recruited.	n/a	Infants whose mothers were still depressed at 6 months had significantly lower Z scores at both 6 months and12 months than infants whose mothers had recovered.
Depression in Mothers and Severity of Depression	Pakistan (20)	HDRS (63)	RCT	n=107 mothers with children up to 30mo, presenting at the outpatient pediatric departments of participating hospitals. 54	Previously translated into Urdu (218)	Results show statistically significant improvement in depression measured by HDRS in the intervention group at 3 and 6 months compared to the control group.

CR term used by authors	Location of included paper	Measure (citation)	Study Design	Sample size and description of population where measure implemented	Adaptation, translation, cross-cultural equivalency, or pretesting	Caregiver Resource Specific Findings
				included in intervention and 53 in control.		
Depression	Pakistan (65)	HDRS (63)	Cross Sectional	n=302 children <2 years, and their mothers were recruited to participate (N=658 children)	n/a	None reported
Maternal Psychiatric Illness (Anxiety & depression)	Pakistan (67)	Hospital Anxiety and Depression Scale (HADS) (66)	Case Control study	n=100 mothers and children 3-36mo: 50 children admitted in nutritional rehabilitation unit with moderate and severe malnutrition were selected as cases while 50 children with normal weight admitted for other illnesses were selected as controls.	n/a	50% of mothers of malnourished cases received high HADS scores reflecting severe psychiatric illness while only 46% of mothers of controls received high scores. Significant relationship observed between child malnutrition and maternal psychiatric illness.
Depression	Rwanda (70)	Hopkins Symptom Checklist (HSCL) (68)	RCT	n= 1498 Primary Caregivers (mean age 36y) of children 6-36mo (n=1084 children), recruited from rural/urban areas	Adapted; detail not stated.	Not reported
Maternal psychological distress	Ethiopia (69)	HSCL (68)	Cross Sectional	n=1,006 Mother child pairs recruited through household sampling	Developed in English and translated and back-translated into Amharic and Affan-Oromifa.	High levels of maternal distress were associated with child underweight
Maternal Depression and anxiety	Tanzania (72)	HSCL (68)	RCT	n=699 Pregnant women aged 20-29 living with HIV were recruited from an urban region of Tanzania	n/a	Maternal depression was positively associated with underweight, stunting, and wasting
Maternal Depressive Symptoms	South Africa (71)	HSCL (68)	RCT	n=958 Mother child pairs; Recruited as pregnant women at peri-urban community level; followed up 36 months. 456 Control and 502 Intervention.	n/a	Primary results report on effectiveness of intervention rather than relationship between maternal mental health and child nutrition
Life Satisfaction	Chad (73)	Author-developed (73)	Cross Sectional	n=98 Caregiver Child Pairs; Female caregivers were interviewed in each rural household and children 12-71 m were evaluated	n/a	Dissatisfied caregivers have shorter children
Satisfaction with Life (Life	India (74)	Author-developed (74)	Cross-sectional	n=451 mothers of children aged 6-59mo in slum areas	Pretested in 25 households	None of the maternal factors was significantly associated with children's dietary diversity.

CR term used by authors	Location of included paper	Measure (citation)	Study Design	Sample size and description of population where measure implemented	Adaptation, translation, cross-cultural equivalency, or pretesting	Caregiver Resource Specific Findings
Satisfaction Scale)						
Emotional, social, cognitive and communication functioning (sub-scales of life satisfaction)	Ghana (24)	PedsQL Family Impact Module (223)	Pre and post evaluation of an 11-month participatory training programme	n=75 caregivers of children with cerebral palsy aged 18mo to 12yo, enrolled at baseline (64 caregivers at endline)	Translated to three languages (Twi, Gruni, and Kusaal); forward and back-translations. Modified following cognitive field testing in three sites.	Emotional, social, cognitive, and communication functioning were all significantly improved from baseline to endline (p<0.0001).
Parental Depression	Nepal (79)	Patient Health Questionnaire (PHQ-9) (75)	Cross-sectional study	n=3158 mothers and children; n=826 fathers and children; Randomly selected mothers and fathers living in rural regions with children between 6-59m	Adapted; detail not stated.	Neither maternal nor paternal depression were associated with child nutritional status.
Maternal depressive symptoms	Peru (80)	PHQ-9 (75)	Cross-sectional study (using DHS 2015)	n=6683 women aged 18 to 49 years old and their children aged 6 to 59 months old	Spanish translation and cultural adaptation using existing version of tool (INEI, 2016)	No association between child anaemia and maternal depressive symptoms in adjusted multivariate models.
Maternal Depression	Kenya (78)	PHQ-9 (75)	Case control study	n=77 Mother child pairs (Cases were 38 children 6-60m admitted to urban hospital with severe acute malnutrition. Matched controls were 38 normal-weight children admitted for other acute ailments. Mean age of mothers: 27.7 years.)	n/a	Data and subsequent multivariable analyses revealed that the odds of maternal depression were significantly higher amongst malnourished cases than normal-weight controls.
Maternal depression	Sudan (81)	PHQ-9 (75)	Case control study	n=178 mothers of children 6-59 admitting to urban hospital. Cases were defined as children aged 6-59 months, admitted with SAM. Controls were age- and sex-matched, well-nourished children admitted to the same hospital with other ailments.	n/a	Overall prevalence of depression was significantly higher among the mothers of cases than controls (41.5% vs. 19.1%, p = 0.001), with 38% displaying severe or moderately severe depression in the case group compared to 17.7% in the control group. Mean score of depression among mothers in the case group (4.47 ¬± 0.48) was significantly higher than those in the control group (2.46 ± 0.33) (p = 0.001).

CR term used by authors	Location of included paper	Measure (citation)	Study Design	Sample size and description of population where measure implemented	Adaptation, translation, cross-cultural equivalency, or pretesting	Caregiver Resource Specific Findings
Primary Care Giver (PCG) Depression	Botswana (76)	PHQ-9 (75)	Case control study	n= 171 children and PCGs (84 malnourished children (cases) and 87 normal weight children matched for age and gender (controls) were recruited from urban primary care clinics along with their primary care givers (mean age: 34y, >95% female))	Translated into local language (Setswana); pilot tested in a smaller local clinic.	Diagnoses of depression in PCGs is significantly associated with child malnutrition (P=0.001)
Depressive symptoms	Tanzania (82)	PHQ-9 (75)	Cross sectional	n=1031 mothers and their children 18-36 months of age	n/a	No association between maternal depression and child nutritional status
Maternal Depressive Symptoms	Ethiopia (77)	PHQ-9 (75)	Prospective, community-based study	n=1560 pregnant women recruited from Empowering New Generations to Improve Nutrition and Economic opportunities (ENGINE) program[7]	Score of 8 or above was taken as a cut off to define depressive symptoms.	Postnatal maternal depressive symptoms were negatively associated with infant feeding practices in the linear mixed effects model (P= 0.001). Prenatal maternal depressive symptoms were not associated with infant feeding practices (P= 0.953)
Psychosocial Distress (Pictogram Suffering Scale)	Cameroon (83)	Author-developed (83)	RCT	n=203 pregnant women, with a mean age of 23.2; n=819 lactating women, with mean age 24.6, with infants at 6m of age, were recruited from urban and rural areas	n/a	The intervention was associated with declines in the psychosocial suffering of pregnant (p=0.000) and lactating (p=0.000) women
Maternal Depression	South Africa (85)	Pitt Inventory (84)	Cohort study	n=1860 Mothers from an urban area with children 6-24 m were recruited to participate	n/a	Children of depressed mothers are at a higher risk for stunting
Psychological Wellbeing (Psychological Satisfaction Scale)	Uganda (86)	Author-developed (86)	Cross Sectional	n=195 Mother with children 0-24m were recruited from rural areas	Adapted from OECD Guidelines (162).	A higher psychological satisfaction scale score was associated with higher probability of stunting (OR [95%CI] = 1.30 [1.06, 1.63])
Maternal Depression	Pakistan (88)	Schedules for Clinical Assessment in	Cohort study	n= 630 mothers with infants were followed-up and	n/a	After adjustment for confounding, there was a positive relationship between maternal depression and risk of infant underweight or stunting at 6 and 12 months postpartum

[7] ENGINE was a 5-year nutrition program funded by United States Agency for International Development (USAID) and implemented from September 2011 to September 2016

CR term used by authors	Location of included paper	Measure (citation)	Study Design	Sample size and description of population where measure implemented	Adaptation, translation, cross-cultural equivalency, or pretesting	Caregiver Resource Specific Findings
		Neuropsychiatry (SCAN) (87)		assessed at 2, 6, and 12m postpartum		
Depression	Pakistan (65)	Structured Clinical Interview for DSM-IV Axis I Disorders (SCID-I) (89,90)	Case control study	n=242 mothers attending infant immunization clinics at the 5 health centers in urban and rural areas	Translated, culturally adapted into Urdu following procedure from previous studies (224).	Infants with depressed mothers were found to have significantly poorer growth rates (based on weight and length z-scores) than infants with non-depressed mothers at 3- and 6-months post-partum
Postnatal depression	Nigeria (92)	SCID-I (89,90)	Case Control study	n=72 Children with Malnutrition, n=72 matched Children without malnutrition. Data was drawn from the Central Community Health and Development database to identify cases as children with malnutrition and age, gender, and residence-matched controls	n/a	Major depression in the postpartum period and current major depression are risk factors associated with child malnutrition
Maternal Depression	India (91)	SCID-I (89,90)	Cohort study	n=211 mother-child pairs; Mothers in this rural, low-income setting were assessed during late pregnancy or 4-6 weeks postpartum and again with their children 15 months later	n/a	Maternal CMD is significantly associated with child stunting (LAZ) at follow-up
Maternal Common Mental Disorders (CMD)	Vietnam (93)	SCID-I (89,90)	Cluster-randomized controlled trial	n=903 pregnant women, married and in their third trimester of pregnancy. All women who fulfilled the criteria for a DSM-IV major depressive episode were recruited into the trial.	n/a	At 6mo and 12mo, prevalence was much higher in control than in intervention. Cluster-adjusted OR for major depression among women in control clusters compared with those in intervention clusters was highly significant and remained so after adjusting for covariates.
Overall Anxiety (Psychological Distress)	Bangladesh (95)	Self-Report Questionnaire (SRQ-20) (94)	Cohort study	n= 1065 women in third trimester of pregnancy, followed up to one year postnatal; rural.	n/a	Infant exposure to maternal CMD was not significantly associated with nutritional status at six months in fully adjusted multivariable analysis
Maternal Mental Health (CMDs)	Nigeria (110)	SRQ-20 (94)	Cross-sectional study	n=204 mothers of children <5yo with SAM, attending CMAM out-patient mal-nutrition clinics in Jigawa	Translated to Hausa and back-translated to English.	Mothers with high SRQ scores (>8) had infants with lower weights on average compared to those with low SRQ scores (5.5 kg versus 6.0 kg, p= 0.003).

CR term used by authors	Location of included paper	Measure (citation)	Study Design	Sample size and description of population where measure implemented	Adaptation, translation, cross-cultural equivalency, or pretesting	Caregiver Resource Specific Findings
Psychological Wellbeing	Bangladesh, Ethiopia, Vietnam (96)	SRQ-20 (94)	Cross Sectional	Mothers of children 0-5yo from all urban and rural households in Bangladesh (n= 4400), Vietnam (n= 4010), Ethiopia (n= 2962)	n/a	High levels of maternal CMD were associated with an elevated risk of child stunting in Bangladesh and risk of child underweight in Vietnam.
Risk of Maternal Common Mental Disorders	Ethiopia, India, Peru, Vietnam (101)	SRQ-20 (94)	Randomized Control Trial	n=37 caregivers and their children with moderate-to-severe cerebral palsy aged 1-11 years in peri-urban Bangladesh	n/a	About three-quarters of caregivers scored above the threshold of 7 points for psychological disturbance on the SRQ-20 anxiety scale (Harding et al. 1980)
Maternal Depression, Mental Distress	Pakistan (111)	SRQ-20 (94)	Cross Sectional	n=11,175 mothers with children under 5 were recruited to participate across the three countries	n/a	Maternal wellbeing was not associated with child Minimum Meal Frequency or Dietary Diversity
Maternal Common Mental Disorders	Brazil (97)	SRQ-20 (94)	Case-control study	n= 172 mothers and infants (82 malnourished cases, 90 controls) recruited from an urban measles clinic	n/a	After controlling for potential confounding factors, maternal mental distress was associated with increased risk of infant undernutrition
Maternal Psychological Distress	Malawi (107)	SRQ-20 (94)	Cross Sectional	n= 2000 mothers of infants age 6-18 m were randomly selected from each country	Adapted; detail not stated.	Results suggest a significant association between high maternal CMD and poor child nutritional status in Vietnam and India but not Peru and Ethiopia
Maternal Common Mental Disorders (CMD)	Peru, Ethiopia, Vietnam, India (102)	SRQ-20 (94)	Case-control study	n= 294 mothers were recruited from an urban area with low rates of childhood malnutrition	n/a	After controlling for confounders, a strong association was observed between maternal CMD and child malnutrition as it doubled the risk of moderate or severe malnutrition in children under 5
Maternal Mental Health	Peru (112)	SRQ-20 (94)	Cohort Study	n=899 HIV positive and negative pregnant women from a low-income peri-urban region; women with live births included with their infants in analysis	n/a	No association observed between maternal psychological distress and continued breastfeeding
Maternal Common Mental Disorders (CMD)	Ethiopia (103)	SRQ-20 (94)	Cohort study	n=7722 Mother child pairs. Over the course of the Young Lives Study, mothers and children age 6-18m were selected from 20 urban and rural sites across the four countries	n/a	In India and Vietnam, a significant association was observed between maternal CMD and child growth from birth through age 8. No significant association was found in Ethiopia or Peru.

CR term used by authors	Location of included paper	Measure (citation)	Study Design	Sample size and description of population where measure implemented	Adaptation, translation, cross-cultural equivalency, or pretesting	Caregiver Resource Specific Findings
Maternal Common Mental Disorders (CMD)	Ethiopia, India, Vietnam, Peru (104)	SRQ-20 (94)	Cross Sectional	n=268 Mother of child 12-59 mo admitted to inpatient pediatric oncology ward and pediatric high dependency unit and their mothers were recruited to participate	Pilot study conducted in each country to adapt the measure to local contexts.	Childhood malnutrition was not found to be a predictor of maternal mental distress; however, mental distress was found to be associated with child stunting
Maternal Distress/Postpartum Depression	Pakistan (26)	SRQ-20 (94)	Two-arm, single-blinded, individual RCT	Pregnant women in third trimester with distress (screen positive on SRQ-20, cut-off score of 9)	n/a [study protocol]	n/a [study protocol]
Maternal Common Mental Disorders	Ethiopia (105)	SRQ-20 (94)	Cluster-randomized controlled trial	n=1302 Mothers and their children (0-4y) were followed from birth to 4 years. Maternal mental health was measured at 1y.	n/a	Maternal depression was associated with decreased HAZ at 2 and 4 years
Maternal Common Mental Disorders (CMD)	Bangladesh, Vietnam, Ethiopia (98)	SRQ-20 (94)	Case-control study	n=234 mother-child pairs in rural area (78 cases, stunted children; 156 controls, non-stunted children); Median child age: 17.2mo.	n/a	Significant relationship between maternal CMD and childhood stunting as children of mothers with CMD were found to be at 3x higher risk for malnutrition.
Maternal Depression	Ghana, Malawi, Burkina Faso (100)	SRQ-20 (94)	Cohort Study	n=5846 mother child pairs; recruited from urban and rural areas to participate in the International Lipid-Based Nutrient Supplements Project	n/a	Maternal Depression was not associated with child growth (LAZ scores)
Maternal psychological distress	Pakistan (88)	SRQ-20 (94)	Cross Sectional	n=125 Mother with children aged 12-60m were recruited from a nutritional rehabilitation center and day care facility in an urban area	n/a	Malnourished children were more than 2x as likely to have a mother with a disability associated with a severe maternal CMD
Maternal Common Mental Disorders	Brazil (99)	SRQ-20 (94)	Cross Sectional	n=7242 Biological mothers and their 1y old children were recruited from urban and rural areas	n/a	Not reported
Maternal Common Mental	Malawi (108)	SRQ-20 (94)	Cross Sectional	n=501 Mothers and their infants with a median age of 9.9 m were recruited	Translated, piloted, and administered in Chichewa.	There was no association between maternal CMD and child underweight; however, there was a relationship between maternal CMD and child stunting

CR term used by authors	Location of included paper	Measure (citation)	Study Design	Sample size and description of population where measure implemented	Adaptation, translation, cross-cultural equivalency, or pretesting	Caregiver Resource Specific Findings
Disorders (CMD)				from a rural district health clinic		
Maternal psychological distress	Malawi (109)	SRQ-20 (94)	Cohort study	n=244 Mother with children admitted to the Nutritional Rehabilitation Unit of a regional hospital were included	Adapted; detail not stated.	Differences in early child weight-gain were not associated with maternal psychological distress
Psychological Distress	South Africa (113)	SRQ-20 (94)	Cohort study	n=1833 Mother-child pairs of children age 5 - 21m were recruited from urban and rural areas	n/a	Childhood stunting was associated with postnatal depressive symptoms
Postnatal depressive symptoms	India (106)	SRQ-20 (94)	Cross Sectional	n=2015 Biological mothers and their 1y old children were recruited from urban and rural areas	n/a	Not reported
Depression	China (115)	Zung Self-Rating Depression Scale (ZSDS) (114)	Cross Sectional	n=2514 Mothers of children under 3 years old	n/a	Depression amongst caregivers has a negative impact on child development
**Healthy Stress Levels**						
Caregiver Stress (regarding feeding)	Bangladesh (95)	Author-developed (95)	RCT	n=37 caregivers and their children with moderate-to-severe cerebral palsy aged 1-11 years in peri-urban Bangladesh	Not stated.	Caregivers had decreased stress with regard to feeding post intervention (P<0.001)
Distress Scale (Maternal Distress)	Vanuatu (116)	Author-developed (116)	Prenatal and postpartum questionnaire and assessments	n=54 Mothers and their infants at 4-12 months of age	Translated to Bislama; Removed redundant questions from Kessler-10 Distress Scale and the CES-D Scale.	No relationship between prenatal distress or diet with BMI Z-scores
Maternal Stress Status	Iran (117)	Author-developed (117)	Cross-sectional study	n=600 mother-infants pairs (12-24 mo) in rural and urban areas	Not stated.	Women with high stress tended to have greater risk of weaning compared with those without stress (RR = 1.67, 95% CI: 0.76–3.65, p = 0.20).
Parenting Stress	South Africa (62)	Parenting Stress Index Short Form (118)	RCT	n=1200 women with HIV with infants 0-12mo, clinic-based support study (656 women under standard care; 544 women under enhanced intervention)	Not stated.	No significant influence of intervention on stress. Parental Stress Index Score <= median of 85 (12 months) was 48.5% in intervention and 44.9% in standard care. OR 0.84 (0.54,1.30, p=0.426).

CR term used by authors	Location of included paper	Measure (citation)	Study Design	Sample size and description of population where measure implemented	Adaptation, translation, cross-cultural equivalency, or pretesting	Caregiver Resource Specific Findings
Worry (sub-scale of quality of life)	Ghana (24)	PedsQL Family Impact Module (22)	Pre and post evaluation of participatory training programme	n=75 caregivers of children with cerebral palsy aged 18mo to 12yo (n=64 at 11-mo endline)	Forward and back-translations by linguistic experts in 3 local languages. Modified after cognitive field testing in 3 sites.	Worry was significantly improved from baseline to endline (p<0.0001)
Maternal Self-Reported Stress	Ghana, Malawi and Burkina Faso (100)	Perceived Stress Scale (PSS) (107,119)	Prospective cohort	Cohorts of mother-child pairs participated in trials conducted as part of the International Lipid-Based Nutrient Supplements Project in Ghana (n=1039), Malawi (n=684 and 1504) and Burkina Faso (n=2619)	Not stated.	Maternal stress was not associated with child LAZ score at 18 months.
Women's Overall Appraisal of the Stressfulness of Their Lives	Mexico (120)	PSS (119)	Group comparison study design	90 mothers of children approximately 2 years old; Non-pregnant women with children approximately two years old in urban setting	Not stated.	No significant group differences were found in the mother's stress perception. Median response in the whole sample comprised being stressed "every now and then" with the median score of 22.5 suggesting moderate stress levels.
Maternal Stress	Burkina Faso (121)	SRQ-20 (94)	Cross-sectional survey	n=1210 caregivers of children 6-12 months old; Used baseline data from a longitudinal cluster randomized trial of HKI's Creating Homestead Agriculture for Nutrition and Gender Equity (CHANGE)	Not stated.	Maternal stress was associated with a lower proportion of children being introduced early to complementary foods
Stressors	Jamaica (10)	Author-developed (10)	Case control study	n=210 mothers of IYC aged 9-30mo (139 undernourished cases; 71 nourished controls), recruited from government health centres in urban areas	Not stated.	Mothers of under-nourished children reported more economic stress than mothers of adequately nourished children(p<0.001). No difference between groups in stressors relating to mother's partner and domestic/community violence.
**Equitable Gender Attitudes**						
Acceptance of domestic violence	Nigeria (126)	DHS Questionnaire (225)	Cross-sectional survey (Secondary data analysis)	n=7532 Mothers of children born in the last five years living in a variety of settings	Pretested, adapted, and translated for Nigerian context.	Women with acceptance of domestic violence (low autonomy) were less likely to have stunted and underweight children.

CR term used by authors	Location of included paper	Measure (citation)	Study Design	Sample size and description of population where measure implemented	Adaptation, translation, cross-cultural equivalency, or pretesting	Caregiver Resource Specific Findings
Tolerance of domestic violence	Malawi (124)	DHS Questionnaire, Several Surveys (122)	Cross-sectional survey (Secondary data analysis)	n= 7348 mother-child pairs Mothers of children born in the last five years living in a variety of settings	Sub-scale of maternal autonomy. Original questionnaires were in English and were translated into the local Chichewa and Tumbuka languages then back-translated into English and pretested in training settings.	Unadjusted model: Mothers with high maternal autonomy were less likely to have stunted children (OR=0.84, 95% CI=0.73, 0.96; p=0.011). Adjusted model: Association between maternal autonomy and child stunting was moderately attenuated by the unfavorable effect of older age and favorable effect of the female sex on stunting (aOR=0.85, 95% CI =0.74, 0.97; p=0.020; Model 2)
Acceptance of domestic violence, as a proxy for women's empowerment	Ghana (123)	DHS Questionnaire (226)	Cross-sectional survey (Secondary data analysis)	Women aged 15 - 49 years with at least one child under 5 years	Pretested, adapted, and translated for Ghanaian context.	Domestic violence, as a proxy for women's empowerment, is negatively associated with child malnutrition.
Domestic violence	28 countries globally[8] (128)	DHS Questionnaire, Several Surveys (122)	Repeated cross-sectional survey (Secondary data analysis)	(Multiple years, years not cited) n = 515639 Mothers of children born in the last five years living in a variety of settings	Pre-testing and translation for each country context.	Acceptability of wife beating is negatively associated with children’s nutritional status.
Beliefs about domestic violence	Uganda (127)	DHS Questionnaire (227)	Cross-sectional survey (Secondary data analysis)	Mothers of children born in the last five years living in a variety of settings: n=1009 in 2006; n=888 dyads in 2011	Pretested, adapted, and translated for Ugandan context.	Beliefs about domestic violence were not associated with child anthropometry in either survey.
Intrinsic Agency	5 countries in sub-Saharan Africa[9] (129)	DHS Questionnaire, Several Surveys (122)	Cross-sectional survey (Secondary data analysis)	n=13780 Mothers of children in 2016, born in the last five years living in a variety of settings	Pre-testing and translation for each country context	Intrinsic agency was positively associated with HAZ in low wealth group (0.03 [0.01, 0.05]), with anemia in middle wealth group (0.03 [0.01, 0.05]), and WHZ in high wealth group (0.05 [0.03, 0.18]).
Attitude towards domestic violence	Lao PDR (132)	MICS Questionnaire (131)	Cross-sectional survey (Secondary data analysis)	n=2970 Mothers of children in 2006, born in the last five years living in a variety of settings	Translated and pre-tested in four villages.	Attitudes towards domestic violence was significantly and negatively associated with height- and weight-for-age
Tolerance towards domestic violence	Lao PDR (132)	DHS Questionnaire (228)	Cross-sectional survey (Secondary data analysis)	n=100 Mothers 17-46 years of age living in semi-urban setting	4 items to assess acceptability of domestic violence in certain scenarios; Categorical variable (High self-esteem, low self-esteem); Answer of "Yes" for at least one item used as composite score	Mothers without stunted children had higher self-esteem than did mothers with stunted children.

[8] Bangladesh, Burkina Faso, Benin, Bolivia, Cambodia, Cameroon, Colombia, Dominican Republic, Egypt, Ethiopia, Ghana, Haiti, Jordan, Kenya, Lesotho, Madagascar, Mali, Malawi, Mozambique, Nigeria, Namibia, Nepal, Rwanda, Senegal, Tanzania, Uganda, Zambia, Zimbabwe.
[9] Ethiopia, Kenya, Rwanda, Tanzania, and Uganda

CR term used by authors	Location of included paper	Measure (citation)	Study Design	Sample size and description of population where measure implemented	Adaptation, translation, cross-cultural equivalency, or pretesting	Caregiver Resource Specific Findings
					for low self-esteem; answer of “No” for all four items used as composite score for high self-esteem.	
Attitude towards domestic violence	Myanmar (125)	DHS Questionnaire (229)	Cross-sectional survey (Secondary data analysis)	n=12885 Mothers of children born in the last five years living in a variety of settings	Translated and pre-tested in one urban and two rural locations.	Odds of child anaemia were higher in children born to mothers who had a tolerant attitude toward domestic violence than those born to mothers who were intolerant of domestic violence
Socio-familial empowerment, (acceptability of domestic violence sub-scale)	10 countries in sub-Saharan Africa[10] (130)	DHS Questionnaire, with Women's Status Module (122)	Cross-sectional survey (Secondary data analysis)	n=15153 Mothers of children born in the last five years living in a variety of settings	Pre-testing and translation for each country context	Attitudes towards domestic violence were a sub-scale in the socio-familial empowerment construct. Except for Zimbabwe, all statistically significant relationships between socio-familial empowerment and appropriate feeding practices were negative
Attitudes towards domestic violence	India (134)	National Family Health Survey (NFHS-2) (133)	Cross-sectional survey (Secondary data analysis)	n=821 mothers of children born in the last five years living in a variety of settings	National pretest in one district; questionnaires for each state were bilingual, in local language and English	There was not a significantly significant difference in the percentage of stunted children between women who approved and did not approve of gender-based violence
Justification of domestic violence	India (136)	National Family Health Survey (NFHS-3) (133)	Cross-sectional survey (Secondary data analysis)	n=9092 mothers of children born in the last five years living in a variety of settings	National pretest in two districts; translated in 18 languages	Children of maternal caregivers who did not justify violence against them for going out without permission, neglecting her children, arguing with her husband, refusing to have sex with her husband and burning food had better nutritional status
Gender Norm Attitudes	Zimbabwe (6)	Gender Norm Attitudes Scale (230)	Cluster-randomized community-based trial	n=3181 mother-infant pairs	Adapted; not stated.	No significant association between gender norm attitudes and infant diet diversity was observed (OR 1.03 (0.89–1.19); p=0.673)
Gender norm attitudes	Zimbabwe (7)	Gender Norm Attitudes Scale (230)	Cluster-randomized community-based trial	n=4025 mothers and growth measured in their 4,073 children	Adapted; not stated	Children of mothers with more egalitarian gender norm attitudes had significantly better linear growth outcomes (LAZ and/or stunting) at 18 months. Results from the trial show that intervention improved adverse associations between child linear growth and maternal inequitable gender norm attitudes and maternal depression.
Perception of Equality Score	Zambia (138)	Author-developed (138)	Cluster-randomised, controlled, non-blinded, impact evaluation	Mothers of children born in the last five years living in a rural setting (baseline n=3044; endline n=3536)	n/a	No impact observed on perceived gender equality score
**Safety & Security**						

[10] Benin, Burkina Faso, Ethiopia, Mali, Niger, Nigeria, Rwanda, Sierra Leone, Uganda and Zimbabwe

CR term used by authors	Location of included paper	Measure (citation)	Study Design	Sample size and description of population where measure implemented	Adaptation, translation, cross-cultural equivalency, or pretesting	Caregiver Resource Specific Findings
Intimate partner violence	Bangladesh (140)	Conflict Tactics Scale (142)	Longitudinal	n=19,874 mothers of children <5yo (from 2007,2011 and 2014 waves of the survey)	Shortened and modified.	Proportions of only one severe form of malnutrition were significantly higher among children of mothers who had experienced IPV (12.66%). Significantly higher proportions of SAM were observed among the children of mothers who had experienced IPV (8.81%)
Intimate partner physical violence	Brazil (61)	Conflict Tactics Scales - Form R (CTS-1) (139,231)	Cross-sectional study	n=217 mothers and their children up to 15 mo old; Data derived from a prospective cohort study that followed the first year of life of newborns attended at four primary care facilities of Rio de Janeiro from June 2005 to December 2009.	18-items used to ask mothers about strategies used by them and their partners to resolve possible disagreements in the last 12 months, and to indirectly identify a situation of violence. Used Portuguese version of CTS-1, applied on the 13th-month of the child's life.	Significant associations were observed between overall physical violence and non-breastfeeding. Couples mutually physical abuse each other are 2,14 times more likely not to offer breastmilk (CI 1,06-4,31), compared to couples who do not physically abuse each other.
Intimate partner violence	Tanzania (82)	Abbreviated IPV module of the Tanzania DHS (232)	Cross-sectional	n=1031 mothers of children 18-36 months of age in Town of Ifakara and surrounding villages	Abbreviated 4 category exposure variables (any IPV, both physical and sexual IPV, only sexual IPV, and only physical IPV)	Exposure to only sexual IPV and both physical and sexual IPV was associated with stunting
Intimate partner violence	Tanzania (58)	Adapted Swahili version of domestic violence module, DHS 2010 (233)	Prospective cohort study	n=1,128 mother-child dyads; Pregnant women over the age of 18 who sought antenatal care at one of two health clinics	Pretested and translated	IPV appeared to be an effect modifier and was therefore included in the stratified analysis; The association between PPD and HAZ was stronger among mother & child pairs where mothers were exposed to IPV during pregnancy (0.50, 95% CI:0.75 to 0.25) than among those without IPV exposure.
Intimate partner violence	Rwanda (70)	Adapted version of domestic violence module, DHS Rwanda (234)	Cluster randomized trial	n=1,498 caregivers (mothers and fathers) with at least one child 6–36 months	Developed and tested during pilot research; forward- and back-translated. Categorical exposure variable to measure perpetration of intimate partner violence by male caregivers (y/n) and victimization of female caregivers (y/n)	Caregivers in program communities were less likely to report victimization to intimate partner violence. No differences observed in father reports of intimate partner perpetration.
Intimate partner violence	28 countries globally[11] (146)	Domestic violence module, DHS (122)	Cross-sectional survey (Secondary data analysis)	n=204,159 women Mothers aged 15 - 49 years old with at least one child under five	32 items; Self- reported maternal lifetime exposure to intimate partner violence was separated into four categories: (i) any; (ii) physical only; (iii) sexual only; and (iv) both physical and sexual. Pre-testing and translation for each country context	Maternal exposure to any intimate partner violence significantly increased the odds of childhood stunting.

[11] Azerbaijan, Bangladesh, Bolivia, Burkina Faso, Cambodia, Cameroon, Colombia, Dominican Republic, Gabon, Ghana, Haiti, Honduras, India, Kenya, Liberia, Malawi, Mali, Mozambique, Nepal, Nigeria, Peru, Republic of Moldova, Rwanda, Sao Tome and Principe, Timor-Leste, Uganda, United Republic of Tanzania, Zambia, Zimbabwe

CR term used by authors	Location of included paper	Measure (citation)	Study Design	Sample size and description of population where measure implemented	Adaptation, translation, cross-cultural equivalency, or pretesting	Caregiver Resource Specific Findings
Domestic violence	Tanzania (145)	Domestic violence module, DHS (232)	Cross-sectional	Mothers of children aged 0-59mo	Five yes or no questions: whether women were justifiably beaten by their partners when (1) they went out without telling their partner, (2) they neglected their children, (3) they argued with their partner, (4) they refused to have sex with their partner, and (5) they burnt food.	Child stunting rates are significantly higher for violence prone households. Stunting is 5 percentage points higher in households where women are beaten when they went out without telling their partners.
Maternal experience of physical and sexual IPV	5 countries globally[12] (147)	Domestic violence module, DHS (DHS, 2021) (122)	Cross-sectional survey (Secondary data analysis)	n=24,905 Ever-married women 15 - 49 years with at least 1 child younger than 5 years	Pre-testing and translation for each country context	When IPV was considered by type, associations between IPV and malnutrition and stunting in Honduras, Kenya, and Malawi remained, although there was no consistent trend whereby specific types of violence were more strongly associated than others with the outcomes.
Maternal exposure to domestic violence	Liberia (144)	Domestic violence module, DHS (122)	Secondary data analysis	n=2467 Women 15-49 years with children 0-4 years of age	Authors created three composite variables: women's experience of physical or emotional DV in prior year; women's experience of any sexual domestic violence in prior year; and whether women witnessed their father beating mother. Variables were analyzed separately and not combined into a scale. Pretested and translated.	Women's experience of sexual domestic violence negatively and significantly associated with child anthropometric status.
Intimate partner violence	Ethiopia (143)	Domestic violence module, DHS (122)	Cross-sectional survey (Secondary data analysis)	n=1307 Women 15 - 49 years old with a child 6 - 23month of age	Women's responses were used to create one exposure variable: exposure to any physical, emotional, or sexual violence vs none. Pretested and translated	Children of mothers who had intimate partner violence had 65% lesser odds of getting a minimum acceptable diet as compared to those who were children of women who did not have an intimate partner violence (AOR: 0.35; 95% CI: 0.16, 0.77)
Maternal exposure to physical or sexual IPV	Bangladesh (142)	Domestic violence module, DHS (235)	Cross-sectional survey (Secondary data analysis)	n=1851 Ever-married women 15 - 49 years with at least 1 child younger than 5 years	8 items; Women's responses were used to create a 4-level categorical variable depending if they had experienced: physical IPV only, sexual IPV only, both physical and sexual IPV, or neither type of IPV. Also created a binary variable measuring whether a mother reported any form of IPV (physical, sexual, or both). Pretested and translated	Maternal experiences of IPV were associated with an increased risk of stunting and underweight among children but not with an increased risk of wasting.
Intimate partner violence	Bangladesh (141)	Bangladesh DHS, IPV questionnaire module (235)	Cross-sectional survey (Secondary data analysis)	n=2041 Ever-married women with at least one under 5 child in a variety of settings	Shortened and modified version of conflict tactic scale (NIPORT et al., 2007). 8 items; Three binary exposure variables were developed based on	Having any stunted children under 5 years remained significantly associated with women ever experiencing any physical (OR, 1.48; 95%CI, 1.23, 1.79), sexual (OR, 1.28;

[12] Egypt, Honduras, Kenya, Malawi, Rwanda

CR term used by authors	Location of included paper	Measure (citation)	Study Design	Sample size and description of population where measure implemented	Adaptation, translation, cross-cultural equivalency, or pretesting	Caregiver Resource Specific Findings
					lifetime experience of any physical partner violence (y/n), sexual partner violence (y/n) and physical and sexual partner violence (y/n).	95% CI, 1.02, 1.61) and physical and/or sexual (OR, 1.51; 95% CI, 1.25, 1.84) violence by their partners.
Intimate partner violence	South Africa (55)	Domestic Violence (148)	Prospective cohort study design	n=458 Mothers who recently gave birth at hospital or one of 10 closest clinics.	Adapted; details not stated.	Both adolescents and adults experienced IPV at the same rate with one in five women having been beaten in the past 12 months before learning about pregnancy. This rate increased significantly once the pregnancy was recognized for both adults and adolescents. (No CR specific findings)
Domestic violence	Pakistan (149)	Author-developed (149)	Cross-sectional study	n=400 Mothers aged 18-49 with at least one child (under five years)	Pretested before use.	Gender based violence was found to have a significant effect on mothers' and children's' health.
Domestic Violence Index, (within Stressors Questionnaire)	Jamaica (10)	Author-developed (10)	Case control study	n=210 Mothers of IYC 9-30mo. Participants were recruited from government health centres in parishes and urban areas.	Not stated	No difference between the nourished and undernourished groups in stressors relating to the mother's partner and domestic and community violence.
Family Violence Against Women	Bangladesh (150)	Author-developed (150)	Longitudinal cohort study	n=3164 Pregnant women living in rural areas	Adapted from Conflict Tactic Scale. Short modified version of WHO Collaborative Study Questionnaire (151,152).	Statistically significant association between mother's exposure to any violence and lower WAZ and HAZ scores at birth, as well as 24 months of age for boys and girls.
Intimate partner violence	Ethiopia (77)	Hurt, Insult, Threaten and Scream (HITS) (153)	Prospective, community-based study	n=1560 pregnant women; Based on ENGINE birth cohort study data (Empowering New Generations to Improve Nutrition and Economic opportunities), a 5-year nutrition intervention program funded by USAID	Not stated.	IPV was negatively associated with infant feeding practices (P= 0.001)
Intimate partner violence	India (154)	Author-developed (154)	Cross-sectional	n=3578 mothers with children under two; February 2014 to September 2015, in a census after the trial intervention covering all households with married women 15-49 years.	n/a.	If a mother had experienced physical violence in the last 2 years, her child had higher odds of being stunted (AOR 1.83; 95% CI 1.21, 2.77)
Presence of domestic violence	India (157)	NFHS (NFHS-1, NFHS-2, NFHS-3), Women's Empowerment	Cross-sectional survey (Secondary data analysis)	Mothers of children born in the last five years living in a variety of settings including NFHS-1, n=89,777; NFHS-2,	National pretest and translation for each survey. 1 item; Dichotomous (Beats wife, does not beat wife); Score refers to whether or not a husband beats a wife if she is unfaithful to him and assess the	Domestic violence is statistically associated with the short-term measures of children’s undernourishment in 1998–99. In 2005–06, domestic violence is positively associated with height-for-age measure.

CR term used by authors	Location of included paper	Measure (citation)	Study Design	Sample size and description of population where measure implemented	Adaptation, translation, cross-cultural equivalency, or pretesting	Caregiver Resource Specific Findings
		Module (133,135)		n=89,199; NFHS-3, n=121,120	extent to which a wife is threatened by physical violence as a proxy for women's empowerment.	
Domestic violence	India (155)	NFHS-2, Domestic violence module (133)	Cross-sectional survey (Secondary data analysis)	n=69,072 Ever-married women aged 15-49	National pretest in one district; questionnaires for each state were bilingual, in local language and English	Statistically significant association between mother's exposure to violence and childhood wasting, stunting, severe underweight for age, and severely low BMI for age.
Emotional violence	India (156)	NFHS-3, Domestic violence module (135)	Cross-sectional survey (Secondary data analysis)	n= 79,729 Ever-married women aged 15-49	Pretested and translated	Mothers' experience of emotional violence was significantly associated with child's low weight for age ($p<.001$) and child's stunting ($p<.001$). Mothers who experienced emotional violence at higher risk of having underweight-U2 children (OR:1.317).
Intimate partner violence	India (159)	NFHS-4, Domestic violence module (158)	Cross-sectional survey (Secondary data analysis)	n=6443 Mothers of IYC 0-12mo	Pretested and translated	No statistically significant associations between experiencing any of the three forms of IPV and infant feeding among mothers of children aged 7 to 12 months.
Intimate partner violence	India (160)	NFHS-4, Domestic violence module (158)	Cross-sectional	n=29,558 women with children <5yo	Not stated	Significantly higher proportion of children were stunted or underweight whose mothers had been emotionally or sexually abused ($p< 0.001$). Children of mothers who experienced at least one form of violence were more likely to suffer from stunting, underweight and wasting conditions compared to those whose mothers did not face any violence.
Prevalence of domestic violence	India (161)	Author-developed (161)	Cross-sectional study	n=820 Women in rural and tribal communities with at least one child 6 - 24 months old	Formative qualitative research. Translated and back-translated into local language; field-tested piloted on random subsample.	In bivariate analysis, mother's experience of psychological abuse and sexual coercion had significant association with child weight-for-age z score ($F=5.2$ $p<.05$). In multivariate analysis, mother's experience of psychological abuse and sexual coercion significantly increased risk of low weight-for-age ($p<.05$).
Freedom from physical violence (Psychological wellbeing subscale)	Uganda (86)	Author-developed (86)	Cross-sectional survey	n=195 Mothers with children 0-24 months in a rural setting	Adapted from OECD Guidelines (162).	A higher psychological satisfaction scale score was associated with higher probability of stunting (OR [95%CI] = 1.30 [1.06, 1.63]).
Intimate partner violence	Togo (164)	WHO's Violence Against Women Instrument (VAWI) (163)	Non-blinded parallel-cluster-RCT	n=2031 mother-child pairs (1035 intervention; 996 controls); Women at least 3 months pregnant and mothers of IYC 0-23mo; mothers of children 24-59mo suffering	n/a.	Women receiving the cash transfers had lower odds of having experienced physical violence in the last 12 (DD=7.9pp, ROR: 0.60, 95% CI: 0.36 0.99, p=0.048). No impact on controlling behavior or emotional violence was observed.

CR term used by authors	Location of included paper	Measure (citation)	Study Design	Sample size and description of population where measure implemented	Adaptation, translation, cross-cultural equivalency, or pretesting	Caregiver Resource Specific Findings
				from SAM were also eligible for the cash transfer program		
**Social Support**						
**Social Capital**						
Group membership	Peru (112)	Author-developed (112)	Longitudinal cohort (Young Lives cohort)	n=2015 mothers of children around 1 year of age, rural and urban; born in 2001and were aged around 1 and 5 years old in 2002 (Round 1) and 2006 (Round 2) respectively.	n/a	Overall maternal group participation was not significantly correlated with the child's HAZ at age 1. Correlation between maternal group participation and the child's HAZ at the age of 1 varies significantly across maternal education. Suggestive evidence of a positive association between maternal group membership and child nutritional status.
Maternal Social Capital and Grandmother Childcare Support	Mexico (120)	Author-developed (120)	Group comparison study design	90 mothers of children approximately 2 years old; Non-pregnant women with children approximately two years old in urban setting	All questions in the questionnaires were based on literature review and long-term experience of the research team within Merida.	Positive association of grand-mothers providing informational support during pregnancy/infancy with one measured of child nutritional, WLZ, status at 2 years.
Religious affiliation	Mozambique (165)	Author-developed (165)	Cross-sectional	n=1811 Mothers with children 6-23 months, rural	n/a	Children of non-affiliated women display the highest level of chronic malnutrition, with 41.7% of them being stunted
Individual social capital	Ethiopia, India, Peru, Vietnam (102)	Short Adapted Social Capital Assessment Tool (166)	Cross-sectional analysis of longitudinal cohort study (Young Lives)	n=7242 children from 4 countries (98% of total Young Lives sample); Biological mother of children aged 6 - 18 months (referred to as 1-year-olds), urban and rural	Shortened version of the Adapted Social Capital Tool (A-SCAT) by Harpham et al. (2002) (166).	Mixed results. Cognitive social capital and support from individuals consistently associated with higher z-scores. Group membership showed no association. Involvement in citizenship activities associated with lower z-scores in some settings. Both acute (weight-for-age) and chronic (height-for-age) indicators of nutritional status show similar patterns of association with maternal social capital. Maternal group membership not associated with either height or weight for age after adjusting. Children whose mothers are involved in citizenship activities had lower HAZ scores in Andhra Pradesh (B.25, 95% CI.43,.07), and lower WAZ scores in Ethiopia (B.17, 95% CI.33,.01), than children of mothers who are not involved. More consistent patterns seen between support from individuals and cognitive social capital. In all locations apart from Andhra Pradesh, receiving support from one or more individuals is associated with increased HAZ and in Vietnam it is also associated with increased WAZ. Similarly, high levels of maternal cognitive social capital are associated with increased HAZ and WAZ in Vietnam and India, and increased WAZ in Andhra Pradesh. No association between maternal

CR term used by authors	Location of included paper	Measure (citation)	Study Design	Sample size and description of population where measure implemented	Adaptation, translation, cross-cultural equivalency, or pretesting	Caregiver Resource Specific Findings
						cognitive social capital and child nutritional status is evident in Peru. Interactions between social capital and poverty.[13]
Social capital of mothers	Philippines (168)	Short Adapted Social Capital Assessment Tool (166)	Cross-sectional survey, random sample	n=413 Children age 6-24 months, rural and urban	n/a	Maternal social capital was found to be significantly associated with nutritional status of children 6 to 24 months old. The association indicated that as the level of maternal social capital increased, the nutritional status of the child also got better, in the same manner that a low level of maternal social capital was significantly associated with poor nutritional status
Maternal Social Capital	Ethiopia (167)	Short Adapted Social Capital Assessment Tool (166)	Cross-sectional survey	n=870 infants aged 6 - 12 months and their mother pairs, rural	Shortened version of the Adapted Social Capital Tool (ASCAT). Adapted for this study population, translated into Afan, Oromo; tested in the field.	Having support from two or more people was associated with higher DDS (OR = 1.84) and minimum dietary diversity (MDD: OR = 5.20) but not with MMF, compared to those without support. Having two or more group memberships was associated with higher DDS (OR = 2.2) but not MDD or MMF, compared to those without group membership. Findings about citizenship activities were mixed for MMF and had no association with DDS or MDD. Cognitive social capital showed no association with DDS or MDD and lower odds of MMF (OR = 0.56). Findings showed that a few components of maternal structural social capital (e.g., having two or more group membership or individual support from two or more people) were related to increased dietary diversity in infants. However, associations with meal frequency were inconsistent or even lower with some social capital components
Social Capital	Myanmar (170)	Social Capital (169)	Cross-sectional study	n=1546 Biological fathers of children 6-59 months, urban and rural	Performed literature review, consulted experts and academics in Myanmar to check adapt to local setting. Pretesting was performed on 150 participants from a township in Myanmar.	Both individual and community levels of social capital of fathers had an influence on child stunting, while only the individual level of social capital of fathers had an influence on child wasting. Relationship between individual-level social capital and child stunting was varied by community-level social capital. Inverse relationship of social supports from the father's social networks at the individual level with child wasting. The community-level social supports related negatively with child stunting, there was no association between cognitive social capital and child undernutrition.
Social Capital Score and Spouse	Zambia (138)	Author-developed (138)	Cluster-randomized, controlled, non-	Households with children 0 - 59.5 months of age in rural areas as part of the RAIN	n/a	see EGNA Section.

[13] In India the impact of household poverty on child height-for-age is much less pronounced among those children whose mother has high levels of cognitive social capital. In Vietnam, support from individuals and household poverty group interact in the prediction of weight-for-age While support from one individual is associated with increased WAZ among all children, the association is more pronounced among children living in non-poor households. Interestingly, support from two or more individuals is associated with a slight reduction in WAZ among all children apart from those living in non-poor households.

CR term used by authors	Location of included paper	Measure (citation)	Study Design	Sample size and description of population where measure implemented	Adaptation, translation, cross-cultural equivalency, or pretesting	Caregiver Resource Specific Findings
Relationship Score			blinded, impact evaluation	project. At baseline, n= 3044 mothers; at endline, n=3536.		
Social Participation	Nepal (171)	Author-developed (171)	Cross-sectional survey	n=1787 mothers of children under 24 months; mothers had an average age of 24.9 years, with range from 15 - 52 years, rural	Adapted, translated and field tested. See Cunningham et al. (2015) for more info on the design/conducting of survey (212).	No significant association between social participation score and child's MMF/DD. During path analysis, social participation was associated with higher exposure to IYCF information, which was associated with dietary diversity.
Trust in religious leaders and institutions	Philippines (172)	Author-developed (172)	Opportunistic, retrospective study	n=1192 Caregivers of the children enrolled in the MCO program - Malnourished Child Outreach (MCO), was a 16-week site-based feeding program for moderately and severely wasted children between the ages of 6 and 60 months, Urban/rural	n/a	Caregivers' trust in religious leaders or church was negatively associated with dropout, suggesting that each increased level of satisfaction or trust was associated with a decreased proportion of dropouts from the treatment program. Authors suggest that when households experiencing extreme poverty trust local religious leaders and institutions, they are more likely to remain enrolled in services provided through these networks.
**Social Network**						
Composition of Mothers' Social Networks	India (173)	Author-developed (173)	Cross-sectional data combined with data from Young Lives study	n=279 Mothers participating in Young Lives with children approximately 1 year of age, rural and urban	n/a	The adjusted results showed that network size and network literacy rate remained positively associated with child nutrition (b¬º0.18,P¬º0.007 and b¬º0.57,P¬º0.028, respectively), and that the association with network non-family (%) was still weak and negative (b¬º20.01,P¬º0.049). The model with all three indicators showed the same results. An increase in network size of one member was associated with an increase of 0.21 in HAZ. Meanwhile, a 50% increase in network literacy rate was associated with an increase of 0.28 in Z-score. First, there was a positive interaction between network size and mother's age (P¬º0.093) only children of mothers younger than 25 years old were unaffected by network size. There was a negative interaction between HH wealth and network literacy rate (P¬º0.088) only among the poorest of the poor (housing quality score,0.20) was there a statistically significant association with network literacy.
In-Camp Network Size	Uganda (174)	Author-developed (174)	Mixed methods observational[14]	n=207 Children under 5 who were born at least 1 year after the household was displaced, IDP camps in Northern Uganda	n/a	Local social networks affect HAZ by increasing the resources available for or directed to child health and nutrition inputs. Increasing local network size by one household (a 25% increase in average network size) leads to between a 0.065 to 0.22 z-score improvement, showing a positive and significant effect of network cohesion on the health and nutrition of the

[14] Using data from Uganda Food for Education Evaluation; and administrative data from WFP qualitative data from focus groups conducted with parents and local leaders.

CR term used by authors	Location of included paper	Measure (citation)	Study Design	Sample size and description of population where measure implemented	Adaptation, translation, cross-cultural equivalency, or pretesting	Caregiver Resource Specific Findings
						youngest household members. Maintaining an additional pre-displacement network member as part of the in-camp network was associated with a .20 HAZ improvement
Kin network	Tanzania (175)	Author-developed (175)	Series of cross-sectional surveys	n=132 Women were all monogamous, all parous, rural	n/a	In a multivariate regression model controlling for baseline age and child sex, children with mothers who had more kin in the study area gained more weight across the 16-month study period than children with fewer kin in the village (partial p = 0.02). Disaggregating the kin term and estimating three additional models showed that the first model including only brothers was not significant. The second model including the term for sisters was significant (F = 3.49, r2= 0.16, p = 0.02), and a third model investigating the effect of subadult female kin showed that the female kin effect was driven almost entirely by the number of sisters a mother had that were under 18 years of age For all ages: These data suggest that, for the majority of mothers, having kin living in the study area is associated with increasing numbers of children surviving to age five, having greater relative weight, and having greater weight gain, and suggest that this effect was modified by household wealth: wealthy households benefit the least from kin and poor households the most.
Personal Network (Social Network Ties)	Mexico (176)	Author-developed (176)	Cross-sectional	n=47 mothers who had participated in the IMSS Nationwide Survey on the Health and Nutritional Status of Children under Two Years of Age in Oaxaca, Mexico	n/a	Of the respondent-level and network-level variables tested here, the percentage of indigenous alters in mother’s networks was found to be the best predictor of the introduction of complementary foods. While they saw some associations between network level variables and introduction of foods, "it is equally important to note that the introduction of a large number of foods was not associated with the respondent-level and network-level variables. For foods that are introduced early, particularly fruits, vegetables, smoothies and juice, there was very little variation in the age of introduction, and thus no effect to explain. However, some foods, such as beans, dairy, eggs and other meats, did have a wider variation in the age of introduction and simply were not explained by the respondent-level and network-level variables studied
Social Network	Brazil (60)	Author-developed (60)	Cross-sectional study	n=228 infant-mother pairs in urban area; Six-month-old infants who used primary health care units and their mothers	n/a	Not statistically significant.
Social Networks	Mexico (177)	Author-developed (177)	Case-control	n=656 Urban and rural mothers with children between	n/a	In rural area, social networks for child care showed statistically significant differences when comparing cases and controls. In

CR term used by authors	Location of included paper	Measure (citation)	Study Design	Sample size and description of population where measure implemented	Adaptation, translation, cross-cultural equivalency, or pretesting	Caregiver Resource Specific Findings
				6 and 23 months of age. Cases comprised stunted children aged between 6 and 23 months. Controls were well-nourished children.		the urban area size of social networks was a statistically significant variable. In rural area household allocation of resources and family organization showed that child being cared for exclusively by mother had a protective effect. Social networks showed that having family networks to provide care for the child entailed a higher risk. In urban area having a small family network increased risk of stunting (OR 2.1 CI1.27-349)
Social Support/ Network	South Africa (62)	Author-developed (62)	Randomized Controlled Trial	n=656 women with HIV under standard care; 544 women with HIV under enhanced intervention; Urban/rural; Infants 0-12m; HIV positive mother	n/a	n/a (did not examine relationship between social support and BF--both outcomes reported but not in connection to each other, only as longitudinal outcomes in intervention and comparison).
Mothers' Social Networks	Bangladesh (178)	Author-developed (178)	Cross-sectional surveys to asses intervention impact (intensive vs. non-intensive and change at 2 yr follow up)	n=2001 mothers of children <2 yrs at endline in 2014 and n=2400 at follow up in 2016; rural.	n/a	In the path analyses for minimum dietary diversity, mothers exposed to 3 components of the interventions had higher scores for social networks of adopters (Œ≤=0.20, 0.44), receiving information (Œ≤=0.72, 1.31), and sharing information (Œ≤=0.32,, 1.10). The social network of adopters and diffusion of information, in turn, were positively associated with both descriptive norms (Œ≤=0.03, 0.08) and injunctive norms (Œ≤=0.06, 0.10), which were associated with higher minimum dietary diversity (Œ≤=0.02,0.09). The indirect effects, obtained by adding the products of the regression coefficients for each path, show that, for minimum dietary diversity, 34%, 42%, and 43% of the total effects of IPC, MM, and CM, respective were explained by improved social networks, diffusion of information, and social norms. The indirect effects for IPC were 61% for early initiation of breastfeeding and 39% for EBF; the indirect effect of MM was 78% for EBF
Social Network/ Support Network	Brazil (61)	Social Network (179)	Cross-sectional study	n=217 mothers and their children up to 15 mo old; Data derived from a prospective cohort study that followed the first year of life of newborns attended at four primary care facilities of Rio de Janeiro from June 2005 to December 2009.	Not stated	In multivariate analyses, associations between overall physical violence and non-breastfeeding remained significant even after adjusting for co-variates (possession of household items, mother current working status, maternal age, social network, and suspected depression).
**Social Support**						

CR term used by authors	Location of included paper	Measure (citation)	Study Design	Sample size and description of population where measure implemented	Adaptation, translation, cross-cultural equivalency, or pretesting	Caregiver Resource Specific Findings
Adequacy of Social Support	Tanzania (43)	Author-developed (43)	Cluster-randomized effectiveness trial	n=Women in 591 households; Households included were food insecure as defined by the community and had a child aged<1 y access to land and planning to farm in the coming year.	Adapted from the Perceived Social Support Scale and a subscale of the A-WEAI.	AWEAI: At baseline, women reported that their husbands had helped with 2.1¬±1.8 household chores in the past month; the intervention increased this by 0.36 tasks (P=0.05). PSSS: At baseline, 78.4% of women reported adequate social support; the intervention increased this proportion by 13.3pp (P=0.01).
Breastfeeding Support	Vietnam (182)	Author-developed (182)	Cross-sectional survey	n=10,834 mothers of children <24 mo old, urban and rural	n/a	No significant relationships between BF support during perinatal period and continued BF at 12 or 24 months
Community Membership and Community Cohesion	India (183)	Author-developed and Community Cohesion (183,184)	Cross-sectional survey, using a two-stage, 30 cluster random sampling design	n=922 Children over 6 months, rural; Patharpratima block of the Sundarbans in West Bengal, India,	Community Cohesion measure available at Paina et al. (2016) (184).	Mean perceived community cohesion score, out of a possible maximum score of nine, was5.41 for children who received minimum acceptable diet and 4.57 for children who did not receive minimum acceptable diet and the difference between these two groups was statistically significant (p= 0.000). The percentage of the head of households reporting being members of at least one community group did not differ significantly (p= 0.90) between household where the children received and did not receive minimum acceptable diet.
Social support scale	Uganda (86)	Duke UNC Functional Social Support Scale (185)	Cross-sectional survey	n=195 Mothers with children 0-24 months, rural	n/a	Mothers with greater social support were more likely to meet the four remaining complementary feeding indicators. Each unit increase in social support was associated with a higher odds of MMF (odds ratio [OR] [95% CI] = 1.38 [1.10,1.73]), the MDD (OR [95% CI] = 1.56 [1.15, 2.11]), and MAD (OR [95% CI] = 1.55 [1.10, 2.21]).
Social Support	Tanzania (72)	Duke UNC Functional Social Support Scale (185)	RCT	n=1,078; n=699 included at the end of the study. Pregnant women with HIV, 71.4% of pregnant women were between the ages of 20 and 29 years and nearly12% were 15-19 years old, urban	n/a	Perceived social support was not significantly associated with underweight or stunting. Although low social support was positively associated with wasting (RR1.50, P<0.01) in the univariate analysis, this effect was no longer significant after adjusting for confounding variables (RR 1.12, P= 0.75).
Social support	Tanzania (187)	Duke UNC Functional Social Support Scale (185)	Randomized placebo-controlled trial	n=795 HIV positive women between 12 and 27 weeks of gestation, urban	Previously validated.	Material and social support was associated with decreased likelihood of BF cessation (RR 0.83; 95%CI 0.68,1.02) Emotional and affective support, however, was not related with breastfeeding cessation. Instrumental and material support was significant in the univariate analysis (RR 0.76; 95% CI 0.63,0.91;P-value=0.004) and when adjusting for other predictors using the Cox proportional hazards model was consistent with the univariate although insignificant at an alpha level of 0.05 (RR 0.83; 95% CI 0.68,1.02;P-value=0.07)

CR term used by authors	Location of included paper	Measure (citation)	Study Design	Sample size and description of population where measure implemented	Adaptation, translation, cross-cultural equivalency, or pretesting	Caregiver Resource Specific Findings
Social Support	Nicaragua (186)	Duke UNC Functional Social Support Scale (185)	Cross-sectional study	n=1371 Caregivers with children 0-35 months of age, rural	Used adapted Spanish version (236).	Children of women with the lowest social support were more likely to consume highly processed snacks and/or SSB, but also had marginally significant higher odds of meeting dietary diversity. No other associations were found between women's social support and infant and young child feeding practices in the adjusted models. With regard to social support, adjusted analyses children aged 6-35 months of women in the lowest social support tertile had significantly higher HAZ and lower odds of stunting compared with women in the highest social support tertile. No significant associations found between women's social support and IYCF. Women in the lowest tertile of social support had infants with the highest HAZ and also the lowest occurrence of stunting.
Family Support Scale	Indonesia (188)	Author-developed (188)	Cross-sectional	n=153 mothers with babies aged 6-12 mo; Conducted in one of the public health centers with the highest incidence of malnutrition in Surabaya, Indonesia.	n/a	All independent variables (knowledge, attitudes, beliefs, income, and family support) had a significant yet a weak correlation with maternal behavior in selecting complementary feeding for family support spearman-rho test (p=0.006, r=0.222)
Household Bargaining Power Sub-scale (social participation)	Nepal (189)	Author-developed (189)	Cross-sectional study	n=2170 mothers/fathers of children 0-59mo; Interviews were conducted with the mother and father of the index child. Our final study sample for this analysis consisted of households which had complete information on both women's and men's bargaining domains (n=2170) for children aged 0-59 mo	Subscale of household bargaining power	Women's social participation were not associated with child HAZ (P>0.94). Men's social participation, however, was positively associated with HAZ (P<0.001).
Male Involvement in Housework	Benin (190)	Author-developed (190)	Cross-sectional survey	n=767 mothers of Children 6 - 59 mo. Mothers age range 15 - 49, rural	n/a	No significant association between male involvement in housework measure and child's dietary diversity score, height for age, weight for height or weight for age.
Maternal Social Support	Ethiopia (77)	Maternity Social Support Scale (MSSS) (191)	Prospective, community-based study	n=1560 pregnant women; Based on ENGINE birth cohort study data.	n/a	Maternal social support (P= 0.002) and social participation (P< 0.001) were positively associated with infant feeding practices
Social Support (tangible and emotional)	Jamaica (10)	Medical Outcomes Study (MOS) Social Support Survey (192)	Case control study	cases (n=139), controls (n=71); IYC were aged 9-30mo. Two groups, undernourished and adequately nourished controls, were enrolled.	Authors made modifications but not described in paper.	No difference between nourished and undernourished groups in the availability of social support

CR term used by authors	Location of included paper	Measure (citation)	Study Design	Sample size and description of population where measure implemented	Adaptation, translation, cross-cultural equivalency, or pretesting	Caregiver Resource Specific Findings
Level of Social Support	Pakistan (26)	Multi-Dimensional Scale for Perceived Social Support (193)	Two-arm, single-blinded, individual RCT	Pregnant women in third trimester with distress (screen positive on Self-Reporting Questionnaire (SRQ), cut-off score of 9)	n/a [study protocol]	n/a [study protocol]
Perceived Social Support	Pakistan (65)	Multi-Dimensional Scale for Perceived Social Support (193)	Cluster-RCT	n=903 Pregnant women aged 16-45 years (married and in their third trimester of pregnancy). All women fulfilled the criteria for a DSM-IV major depressive episode, rural.	Used questionnaires that had been translated and adapted in previous studies.	n/a
Non-Maternal Adult Female Household Members	Ethiopia (194)	Author-developed (194)	Cross-sectional (DHS)	n=7100 mothers with children aged 6-59 months	n/a	Prevalence of child undernutrition decreases with the number of non-maternal AFHMs living in the household. having an additional non-maternal AFHM is associated with, on average, a 0.22 unit of SD increase in height or 0.10 unit of SD increase in weight, after controlling for potential confounders.
Social Support Scale	Pakistan (20)	Oslo Social Support Scale (OSSS-3) (195)	RCT	n=107 mothers with children up to 30mo (54 in intervention, 53 in control); All mothers with children up to 30 months old, presenting at the outpatient pediatric departments of the participating hospitals were approached and invited to participate	Translated to Urdu.	Scores for social support improved significantly for the intervention group as compared to the control group.
Paternal Involvement in Childcare	Vietnam (196)	Author-developed (196)	Cross-sectional study	n=547 random sample of 547 children under 3 years of age from intact families and their biological parents, Urban/rural	Includes involvement in daily child-care activities, involvement in health care, and indirect involvement by assisting with housework.	Lack of paternal involvement in providing access to preventive health care for children was significantly associated with underweight among children (POR = 1.75, p = .025), controlling for child's age, household economic status, mother's education. Other factors of paternal involvement were not significantly associated with prevalence of underweight, they all showed tendency to reduce likelihood of child's being under-weight. Larger positive coefficient of association between "not involved" and underweight in children was found in the urban setting. Paternal involvement in child care and housework was not associated with stunting. Two factors representing paternal involvement in child health were marginally associated with stunting; p values equal to .06 and .03, respectively. Children whose fathers were not involved in providing health care were about 1.7 times more likely to be

CR term used by authors	Location of included paper	Measure (citation)	Study Design	Sample size and description of population where measure implemented	Adaptation, translation, cross-cultural equivalency, or pretesting	Caregiver Resource Specific Findings
						stunted than children whose fathers were involved. Paternal involvement in housework and daily child care was not significantly associated with prevalence of stunting
Perceived Social Support (to employ a particular feeding method)	Nigeria (197)	Author-developed (197)	Cross-sectional survey	n=240 mothers and 246 grandmothers; women who currently had a child aged 4-24 months. Also included were grandmothers who would have had a grandchild in the same age range, rural town	Pretested whole questionnaire, not specific to social support.	Grandmothers perceived significantly more support and encouragement to practice hand/forced-feeding while opposite case for bottle feeding. Both sets of respondents felt equally encouraged to use cups and spoons and the level of support perceived was highest for this method. Perceived social support from older women and other people in the house to forced-feed was the main factor associated with its use by mothers
Perceived Social Support	Zimbabwe (6)	Perceived Social Support (5)	2x2 cluster randomized community-based trial	n=3181 mother-infant pairs followed to 12 months; Pregnant women enrolled in SHINE trial	Adapted from Interpersonal Support Evaluation List (198) and the Medical Outcomes Study Social Support Survey (192).	Social support was not significantly associated with infant diet diversity (unadjusted OR 1.23(1.08-1.42); p=0.003; adjusted OR 1.18 (1.01-1.37); p=0.039)
Perceived Social Support	Zimbabwe (7)	Perceived Social Support (5)	2x2 cluster randomized community-based trial	n=4,025 mothers; Pregnant women enrolled in SHINE trial.	Adapted from Interpersonal Support Evaluation List (198) and the Medical Outcomes Study Social Support Survey (192).	Similar to 18-month findings, social support was associated with child linear growth at 12 months.
Social support	Brazil (38)	Author-developed (38)	Cross-sectional study	n=595 mothers of children 6-24m in urban area	Adapted from Medical Outcomes Study (MOS) Social Support Survey and Adams' Social Network measure (192,199).	Having a source of material support was associated with higher child WHZ and WAZ. Higher maternal affectionate support was positively associated with child WHZ and WAZ. Negative relationship between mothers' access to support when dealing with a relationship problem and children's HAZ. Mothers without material support had children with a 0.05 WAZ on average, compared to 0.4 for those who had at least one person (p 0.01). Children of mothers who reported at least one person to borrow food/money had 0.3 higher average WHZ compared to children of mothers with no one to borrow from. Scoring in highest range of affectionate support was associated with a 0.2 higher average WHZ, than mothers reporting lowest levels of affectionate support. Children whose mothers had higher levels of social interaction on the MOS subscale (scoring 71 - 85), had on average 0.3 higher WHZ; no significant relationship between other MOS domains
Self-Reported receiving social support from others	Kenya (78)	Author-developed (78)	Matched Case Control	n=39 cases and 39 controls (78 Mother-Child Dyads); Cases were malnourished children ages 6-60 months admitted with severe acute malnutrition. The controls were gender and age-matched children who	n/a	The majority of the mothers of cases (71.8%) and controls (84.6%) reported that they were receiving social support from family members or friends.

CR term used by authors	Location of included paper	Measure (citation)	Study Design	Sample size and description of population where measure implemented	Adaptation, translation, cross-cultural equivalency, or pretesting	Caregiver Resource Specific Findings
				were normal weight and admitted to the same hospital for acute ailments. Urban		
Social support, informational support	Uganda (127)	Author-developed (127)	Cross-sectional (Secondary data analysis)	n=1009 women with child 0-23 months in 2006; n= 888 dyads in 2011. Included BF and non-BF, Urban and rural.	Conducted formative research to inform the survey but not specific to social support measure (237).	n/a
Social Support System Adequacy (degree of social interaction, perceived social support, received social support)	Brazil (200)	Author-developed (200)	Case control	n=101 cases [malnourished children WAZ below 5th percentile]; 200 control [WAZ above 25th percentile]; children aged 12 - 23 months, predominantly urban	n/a	The only variable related to the presumed availability of support (support variable) independently related to malnutrition was the absence of a partner living with the mother. This effect was significant among children of all levels of per capita family income
Social Support	Chad (73)	Author-developed (73)	Cross-sectional survey	n=98 Children 12-71 months of ages, rural	n/a	Caregivers who received more help to accomplish their domestic or productive tasks had taller children. Consultation of older women was a significant predictor of child height status.
Social Support Index	Kenya (201)	Author-developed (201)	Quasi-experimental	n=554 people at baseline (258 mothers, 165 grandmothers, and 131 fathers) and n=509 at endline; mothers, fathers, and grandmothers from households with infants 6 - 9 months old, rural	Based on previous infant feeding research conducted in Kenya (238,239).	As number of social support actions increased in the 3 study groups, likelihood of a MMF in the past 24 hours increased significantly. Significant association in grandmother intervention area on MDD (OR, 1.19; CI, 1.01 to 1.40; P=.04). No significant effects were found on MAD.
Social Support Measurement	India (106)	Author-developed (106)	Longitudinal (Cohort)	n=1833 mothers of children aged 5 - 21months old, Urban/rural.	Description of measures' development as part of the Young Lives Study (240).	Social Support not associated with stunting; effect of pregnancy intention and postnatal depressive symptoms on early childhood stunting were not mediated by social support
Social Support Resources	Jamaica (202)	Author-developed (202)	Longitudinal study	n=109 completed study (150 recruited); Mothers were heads of household, had engaged in income-generating activity during at least the first two trimesters of pregnancy, had another child five years of age or younger, and had just given birth by normal delivery to an infant whose birthweight was 2,500 g or more. Urban/rural	Formative interviews with 15 postpartum women in the area, including open-ended questions concerning present work situation and work histories, current household structure, how they managed to combine income generating work and childcare, childcare and infant feeding practices.	There is some indication that infants whose mothers received very little in cash contributions from other household members fared less well than those infants whose mothers received $100J or more. Infants whose mothers shared food preparation with persons less than 15 years old had a lower percent of median weight-for-age than those whose mothers were assisted by food preparers over 16 years of age

CR term used by authors	Location of included paper	Measure (citation)	Study Design	Sample size and description of population where measure implemented	Adaptation, translation, cross-cultural equivalency, or pretesting	Caregiver Resource Specific Findings
Social Support	Myanmar (203)	Social Support System Adequacy (200)	Cross-sectional study	n=320 children aged 6-59 months living at the IDP camp; youngest child in a selected household	Adapted; details not provided.	The bivariate analyses showed no types of social supports were significantly associated with stunting
Social Support	South Africa (205)	Social Support Scale (204)	RCT	n=1144 at baseline, post-birth assessment n=1152, 18-month assessment n=1039; Urban; participants in the Philani project with children 0-24 months of age	n/a	Social support was significantly associated with better growth
Sources of Social Support (Maternal Grandmother and Partner Support for Breastfeeding)	Brazil (206)	Author-developed (206)	Secondary data analysis from a RCT	n=228, 237, and 207 mothers at 6, 12, and 24 months; Adolescent mothers participating in a RCT (that involved maternal grandmothers)	n/a	Maternal grandmother supports breastfeeding was positively associated with BF at 6 and 12 months. Partner supports breastfeeding at 12 months only
Support from Husband	India (74)	Author-developed (74)	Cross-sectional study	n=451 children aged 6-59mo in slum settlements (of which 248 were 6-23mo); Data were collected from 506 households with at least one child under 5 years whose mother was alive and staying with child. If more than one child, last born was selected for data collection. Excluded were acutely ill children or those with congenital defects, those whose mothers had migrated to city in past 6 months or not willing to participate.	Pretested in 25 households. All interviews were conducted in local languages.	Univariate analyses: As to feeding practices for children under 24 months, not many maternal factors demonstrated association.<br>Multivariate analyses: In model 1, leisure time/day (OR 2.47; 95% CI 1.2, 5.07) demonstrated a significant association with meal frequency. None of the maternal factors was significantly associated with children's dietary diversity except for media exposure and BMI, which showed a non-significant association (p<0.1). <0.1). In model 2, available leisure time (OR 2.75; 95% CI 1.25, 6.06) and participation in budgeting (OR 1.97; 95% CI 1, 3.86) demonstrated significant associations with children's meal frequency. Except for BMI demonstrating a weak association (OR 1.7; 95% CI 0.92, 3.13), none of the maternal factors was associated with dietary diversity.
Support in Household Chores and Perceived Instrumental Support	Bangladesh, Ethiopia, Vietnam (96)	Author-developed (96)	Cross-sectional study	Mothers and their <5 years old children in Bangladesh n= 4,400, Vietnam n= 4,029, and Ethiopian=2,746	n/a	n/a
Women's Perceived Social Networks	Mali (18)	Author-developed (18)	Cross-sectional survey	n=505 caregivers ever-married women, ages 15 - 49; rural (total of 402 children under five years of age)	Formative qualitative research conducted with women prior to survey design.	Felt control was significantly greater among women who reported support from mother-in-law, but other social support variables did not have significant relationship with women's self-efficacy. However, consistent across all the models is the

CR term used by authors	Location of included paper	Measure (citation)	Study Design	Sample size and description of population where measure implemented	Adaptation, translation, cross-cultural equivalency, or pretesting	Caregiver Resource Specific Findings
						deleterious effect on child nutrition of having a supportive mother-in-law. Whereas women who perceive mothers-in-law as sources of support have greater social power, the informational and/or material resources controlled by the mother-in-law do not appear to benefit the nutritional well-being of children. Also, the likelihood of wasting among children 5 years of age and younger increases as the size of their mother's social network increases.
**Time Sufficiency** (n=17)						
Caregiver Workload	Chad (73)	Author-developed (73)	Cross-sectional survey	n=64 rural households with children 12-71 months (n=136 children); Interviewed heads of household (on economic resources) and all women in household about resources for care and childcare variable.	Based on previous survey in same community and literature. Pre-tested with 10 households.	Caregiver workload was not significant predictor of child nutritional status and therefore were not included in the models, no descriptive results reported. Women involved in handicrafts spent less time on domestic activities (P=0.07) and had less leisure time (P<0.01), often mentioned in interviews as spent in child care.
Employment Status (Number of income-related work hours per week)	Nepal (207)	Author-developed (207)	Cross-sectional	n=451 mothers of children 6-59 months in slum settlements of Hyderabad, India (n=248 IYC were 6-23mo). Excluded children with acute illness or congenital defects, and mothers who had migrated to city in past 6 months.	Full survey pre-tested in pilot study n=26 in one of the study sites.	Eighty-four (19%) mothers did not get even 2 hours leisure time per day.
Leisure Time/Day	India (74)	Author-developed (74)	Mixed methods, descriptive	n=92 rural households across three groups representing increasing levels of dairy intensification	Pretested in 25 households.	Found increased workload, especially for medium-intensity households. (Did not report results related to IYCF but said women's reports in FGDs suggest that dairy intensification could have an impact on women's time use and impact their care giving activities and child nutrition.)
Maternal Time Use	Mali (208)	Author-developed (208)	Cross-sectional survey	n=65 Bambara mothers of children aged 12 -36 month, excluding twins, with complete data. Rural (within 20 mi of town)	Not stated.	Women spent a mean of 5.3 hours doing domestic work, including child-care, and 2.9 hours in income-generating activities. Time spent preparing food depended on number of women in compound taking turns to cook for extended family.
Maternal Work Patterns	Jamaica (202)	Author-developed (202)	Longitudinal study -- survey	n=109 urban and semi-rural mothers who were heads of household, engaged in income-generating activity	Based on open-ended interviews with 15 postpartum women from health facilities in the area.	12 women were working at six weeks postpartum, 7 working at home. From 3 months, the majority reached place of work in under one-half hour, but some traveled > 1 hour, and two who, at nine months postpartum, reported

CR term used by authors	Location of included paper	Measure (citation)	Study Design	Sample size and description of population where measure implemented	Adaptation, translation, cross-cultural equivalency, or pretesting	Caregiver Resource Specific Findings
				during at least first two trimesters of pregnancy.		traveling >2 two hours. Most women reported working very long hours: at 3 months postpartum, over 80% of working women reported spending 6h/d or longer on income-generating activities; women with 2 occupations reported working up to 16 hours per day.
Number of days mothers spent in farming	Tanzania (209)	Author-developed (209)	Cross-sectional	n=152 main caretakers of child under 5 y and head of household, in one rural village	Based on study in 1987/1988 (241) and pretested in 15 households.	Nearly all women (>90%) reported farming as their main occupation, and most women (70%) reported additional income-generating activities. Women worked a median of 48 (range 5-208) d in fields in agricultural season of 2009/2010.
Perceived Time Stress	Zimbabwe (7)	Perceived Time Stress (5)	2x2 cluster randomized community-based trial	n=3181 mother-infant pairs followed to 12 months; Pregnant women enrolled in SHINE trial.	n/a	No association was observed between perceived time stress infants fed diverse diet in unadjusted model (OR 1.03 (0.94-1.13); p= 0.500)
Perceived Time Stress	Zimbabwe (7)	Perceived Time Stress (5)	2x2 cluster randomized community-based trial	n=4,025 mothers; Pregnant women enrolled in SHINE trial	n/a	Greater prenatal time stress was associated with lower child LAZ at 18 months, but this association did not reach statistical significance (0.04; 95% CI: 0.08, 0.004, p= 0.07).
Time Use (including workload and leisure)	Nepal (212)	WEIA Time Allocation Module (210)	Cross-sectional survey (from Suaahara; secondary data analysis)	n=1787 rural mothers of children <24 months; mean age of children = 12 months; mean age of mothers = 25 y. 32 % of mothers had no formal education or less than one year of schooling.	2 indicators, each scored adequate/inadequate and each weighted 0.10 of overall empowerment score. 1. Workload (productive and domestic workload based on detailed 24-hr time diary, summing hours in work-related tasks as primary activity + 50% of time in work-related tasks as secondary activity; inadequate = excessive workload if >10.5 hours worked in past 24 hrs); 2. Satisfaction with available time for leisure activities (visiting, TV, radio, movies, or sports: one item, 10 pt scale. Inadequate = <5; Adequate = 5- 10).	Workload greater than 10.5h/d was among the 5 indicators with lowest proportion of women scoring adequate (38%). Satisfaction with amount of time available for leisure activities (83 %) was among the highest 5 indicators.
Women's Time Use (by categories of activity).	5 countries[15] (214)	Workload indicator from WEIA (210)	Cross-sectional survey (secondary	Primary female and male household members in Bangladesh (n=3503), Cambodia (n=1491) Ghana (n=1574), and Mozambique	n/a (secondary data analysis)	In countries other than Bangladesh: women's total work time > than men's. In all 5 countries, women's time on reproductive work > than men's; men's time on productive work > women's.

[15] Bangladesh, Nepal, Cambodia, Ghana, Mozambique

CR term used by authors	Location of included paper	Measure (citation)	Study Design	Sample size and description of population where measure implemented	Adaptation, translation, cross-cultural equivalency, or pretesting	Caregiver Resource Specific Findings
			data analysis)	(n=1707); and mothers of children <5, and their husbands, if available, in Nepal (n=3807). Restricted to agricultural households.		
Workload Domain	Nepal (171)	Workload indicator from WEIA (210)	Cross-sectional survey (from Suaahara baseline data)	n=1787 children < 24 m and their mothers, in rural area. Mothers average age 24.9 y, range 15 - 52 y.	Used as indicator of workload.	Mean number of hours worked per day: 11 (SD:2.98). Range: 0.58-18.9 hours.
Workload (Time spent on work activities)	Nepal (189)	WEIA Time Allocation Module (210)	Cross-sectional study	n=2170 mothers/fathers of children 0-59mo; households with complete information on both women's and men's bargaining domains.	Not stated.	Women's workload (P<0.001) was negatively associated with HAZ. There was no association between men's workload and child HAZ.
Time Allocation	Zambia (138)	WEIA Time Allocation Module (210)	Cluster-randomized, controlled, non-blinded, impact evaluation design	rural households with children <5 yrs; Baseline n= 3044 mothers; Endline n= 3536 mothers	Time allocation was measured only at end so for this variable, compared intervention and control at endline.	Time spent on leisure I: 14h10m; C: 14h35m; agriculture work I: 3h32m; C: 2h55m; domestic work I: 4h41m. C: 5h0m; other work I: 1h10m. C:0h59m.
Time Use	Nepal (213)	WEIA Time Allocation Module (210)	Cross-sectional survey (secondary data analysis)	n=3332 rural households with children < five years, in three agroecological zones of mountains, hills, and terai, in which the female respondent reports working in agriculture as primary or secondary occupation.	2 indicators, each scored adequate/inadequate and each weighted 0.10 of overall empowerment score. 1. Workload (productive and domestic workload based on detailed 24-hr time diary, summing hours in work-related tasks as primary activity + 50% of time in work-related tasks as secondary activity; inadequate = excessive workload if >10.5 hours worked in past 24 hrs); 2.	Hours spent in paid & unpaid work (all households, n=3117): 11.19hrs (SD: 2.95).
Women's Time Use	Tanzania (43)	A-WEAI (210)	Cluster-randomized effectiveness trial	n=591 food insecure households (as defined by the community) with child aged <1yo; access to land and planning to farm in the	Not stated	AWEAI: The intervention did not increase women's time spent on household work and child care.

CR term used by authors	Location of included paper	Measure (citation)	Study Design	Sample size and description of population where measure implemented	Adaptation, translation, cross-cultural equivalency, or pretesting	Caregiver Resource Specific Findings
				coming year; interviewed women in household		
Time Poverty	Bangladesh (211)	Time poverty (242)	Cross-sectional survey (secondary data analysis)	n=5124 rural households with 6-23-month-old; Representative sample (from Bangladesh Integrated Household Survey)	Secondary data analysis based on time poverty approach of Bardasi & Wodon (2006) (242).	No clear evidence of a relationship between time poverty and household dietary diversity or of a multiplicative effect between the income and time poverty on household dietary diversity.
Women's Time Use	Burkina Faso (215)	Author-developed (215)	Longitudinal cluster-RCT and process evaluation	n=1,242 women with children 3-12 months old in rural area	n/a	For women in intervention villages, an increase in time spent on agriculture activities was not associated with any of the child health and nutrition indicators for which a positive programme impact was found in the primary analyses including wasting

References

1. Zongrone A, Menon P, Pelto G, et al. The pathways from a behavior change communication intervention to infant and young child feeding in Bangladesh are mediated and potentiated by maternal self-efficacy. *J Nutr*. 2018;148(2):259–66.

2. Teti DM, Gelfand DM. Behavioral competence among mothers of infants in the first year: The mediational role of maternal self-efficacy. *Child Development*. 1991 Oct 1;62(5):918–29.

3. Surkan P, Kawachi I, Ryan L, et al. Maternal depressive symptoms, parenting self-efficacy, and child growth. *Am J Public Health*. 2008;98(1):125–32.

4. Salarkia N, Omidvar N, Zaeri F, et al. Mother's self-efficacy mediates the relationship between household food insecurity and maternal infant feeding styles. *Matern Child Health J*. 2016;20(3):602–12.

5. Matare CR, Mbuya MNN, Pelto G, et al. Assessing maternal capabilities in the SHINE trial: Highlighting a hidden link in the causal pathway to child health. *Clin Infect Dis*. 2015;61(suppl 7):S745–51.

6. Matare CR, Mbuya MNN, Dickin KL, Constas MA, Pelto G, Chasekwa B, et al. Maternal capabilities are associated with child caregiving behaviors among women in rural Zimbabwe. *J Nutr*. 2021;151(3):685–94.

7. Tome J, Mbuya M, Makasi R, et al. Maternal caregiving capabilities are associated with child linear growth in rural Zimbabwe. *Matern Child Nutr*. 2021;17(2):e13122.

8. Gilmore L, Cuskelly M. Factor structure of the Parenting Sense of Competence scale using a normative sample. *Child Care Health Dev*. 2009 Jan;35(1):48–55.

9. Dumka LE, Stoerzinger HD, Jackson KM, et al. Examination of the cross-cultural and cross-language equivalence of the Parenting Self-Agency Measure. *Family Relations.* 1996;45(2):216–22.

10. Baker-Henningham H, Powell C, Walker S, et al. Mothers of undernourished Jamaican children have poorer psychosocial functioning and this is associated with stimulation provided in the home. *Eur J Clin Nutr*. 2003;57(6):786–92.

11. Ajzen I. Perceived behavioral control, self-efficacy, locus of control, and the Theory of Planned Behavior. *Journal of Applied Social Psychology*. 2002;32(4):665–83.

12. Mutiso J, Okello J, Lagerkvist C, et al. Effect of nutrition education and psychosocial factors on child feeding practices: findings of a field experiment with biofortified foods and different women categories. *Ecol Food Nutr*. 2018;57(4):346–71.

13. Sarrassat S, Ganaba R, Some H, et al. Suboptimal infant and young child feeding practices in rural Boucle du Mouhoun, Burkina Faso: Findings from a cross-sectional population-based survey. *PLoS One*. 2019;14(11):e0224769.

14. Anjomshoa H, Mirzaee M, Iranpour A. The application of Social Cognitive Theory on mothers' feeding practices for children aged 6 to 24 months old in Iran. *International Journal of Pediatrics-Mashhad*. 2018;6(7):7983–97.

15. Kamiya Y, Nomura M, Ogino H, et al. Mothers' autonomy and childhood stunting: evidence from semi-urban communities in Lao PDR. *BMC Womens Health*. 2018;18(1):70.

16. Rafieyan-Kopaei Z, Fathian-Dastgerdi Z, Tarrahi M, et al. Effectiveness of message-framing intervention on complementary feeding related behaviors among mothers with infants aged 4-8 months: a 3-arm randomized controlled trial. *Ital J Pediatr*. 2019;45(1):158.

17. Prasopkittikun T, Tilokskulchai F, Sinsuksai N, et al. Self-Efficacy in Infant Care Scale: Development and psychometric testing. *Nurs Health Sci*. 2006;8(1):44–50.

18. Simon D, Adams A, Madhavan S. Women's social power, child nutrition and poverty in Mali. *J Biosoc Sci*. 2002;34(2):193–213.

19. Herdman M, Gudex C, Lloyd A, et al. Development and preliminary testing of the new five-level version of EQ-5D (EQ-5D-5L). *Qual Life Res*. 2011;20(10):1727–36.

20. Husain N, Kiran T, Shah S, et al. Efficacy of learning through play plus intervention to reduce maternal depression in women with malnourished children: A randomized controlled trial from Pakistan. *J Affect Disord*. 2021;278:78–84.

21. Hays RD, Sherbourne CD, Mazel RM. The RAND 36-Item Health Survey 1.0. *Health Econ.* 1993;2(3):217–27.

22. Varni JW, Sherman SA, Burwinkle TM, et al. The PedsQL Family Impact Module: Preliminary reliability and validity. *Health Qual Life Outcomes*. 2004;2(1):55.

23. Polack S, Adams M, O'banion D, et al. Children with cerebral palsy in Ghana: malnutrition, feeding challenges, and caregiver quality of life. *Dev Med Child Neurol*. 2018;60(9):914–21.

24. Zuurmond M, O'Banion D, Gladstone M, et al. Evaluating the impact of a community-based parent training programme for children with cerebral palsy in Ghana. *PLoS One.* 2018;13(9):e0202096.

25. WHO. WHOQOL-100: The 100 Questions With Response Scales. Geneva: World Health Organization; 1995.

26. Huma Z, Gillani A, Shafique F, et al. Evaluating the impact of a common elements-based intervention to improve maternal psychological well-being and mother-infant interaction in rural Pakistan: study protocol for a randomised controlled trial. *BMJ Open.* 2021;11(7):e047609.

27. Ali BS, Reza H, Khan MM, et al. Development of an indigenous screening instrument in Pakistan: the Aga Khan University Anxiety and Depression Scale. *J Pak Med Assoc*. 1998;48(9):261–5.

28. Saeed Q, Shah N, Inam S, et al. Maternal depressive symptoms and child nutritional status: A cross-sectional study in socially disadvantaged Pakistani community. *J Child Health Care.* 2017;21(3):331–42.

29. Beck AT. An Inventory for Measuring Depression. *Arch Gen Psychiatry*. 1961;4(6):561.

30. Feldens C, Vitolo M, Rauber F, et al. Risk factors for discontinuing breastfeeding in southern Brazil: a survival analysis. *Matern Child Health J.* 2012;16(6):1257–65.

31. Beck AT, Steer RA, Brown GK. BDI-II Manual. San Antonio, TX: The Psychological Corporation.; 1996.

32. Chee Din MA, Fahmi Teng NIM, Manaf ZA. Is maternal depression a risk factor for malnutrition among malay children? A case-control study in Selangor, Malaysia. *Malaysian Journal of Medicine and Health Sciences*. 2021;17:168–74.

33. Radloff LS. The CES-D Scale: A Self-Report Depression Scale for Research in the General Population. *Applied Psychological Measurement*. 1977;1(3):385–401.

34. Wright A, Tulloch-Reid M, Chang S, et al. Maternal characteristics influence infant feeding styles in Caribbean women. *Public Health Nutr*. 2021;1–12.

35. Aboud F, Singla D, Nahil M, et al. Effectiveness of a parenting program in Bangladesh to address early childhood health, growth and development. *Soc Sci Med*. 2013;97:250–8.

36. Black M, Baqui A, Zaman K, et al. Maternal depressive symptoms and infant growth in rural Bangladesh. *Am J Clin Nutr*. 2009;89(3):951S-957S.

37. Nahar B, Hossain I, Hamadani J, et al. Effect of a food supplementation and psychosocial stimulation trial for severely malnourished children on the level of maternal depressive symptoms in Bangladesh. *Child Care Health Dev*. 2015;41(3):483–93.

38. Surkan P, Ryan L, Carvalho Vieira L, et al. Maternal social and pyschological conditions and physical growth in low-income children in Piauí, Northeast Brazil. *Soc Sci Med*. 2007;64(2):375–88.

39. Mohammed H, Marquis G, Aboud F, et al. Pre-pregnancy iodized salt improved children’s cognitive development in randomized trial in Ethiopia. *Matern Child Nutr*. 2020;16(3):e12943.

40. Wemakor A, Mensah K. Association between maternal depression and child stunting in Northern Ghana: a cross-sectional study. *BMC Public Health*. 2016;16(1):869.

41. Wemakor A, Iddrisu H. Maternal depression does not affect complementary feeding indicators or stunting status of young children (6-23 months) in Northern Ghana. *BMC Res Notes*. 2018;11(1):408.

42. Nguyen P, Friedman J, Kak M, Menon P, Alderman H. Maternal depressive symptoms are negatively associated with child growth and development: Evidence from rural India. *Matern Child Nutr*. 2018;14(4):e12621.

43. Santoso M, Bezner Kerr R, Kassim N, Martin H, Mtinda E, Njau P, et al. A nutrition-sensitive agroecology intervention in rural Tanzania increases children's dietary diversity and household food security but does not change child anthropometry: results from a cluster-randomized trial. *J Nutr*. 2021;151(7):2010–21.

44. Uher R, Goodman R. The Everyday Feeling Questionnaire: The structure and validation of a measure of general psychological well-being and distress. *Social Psychiatry and Psychiatric Epidemiology*. 2010;45(3):413–23.

45. Trinh TA, Feeny S, Posso A. Rainfall shocks and child health: the role of parental mental health. *Climate and Development*. 2021;13(1):34-48.

46. Cox JL, Holden JM, Sagovsky R. Detection of postnatal depression: Development of the 10-item Edinburgh Postnatal Depression Scale. *Br J Psychiatry*. 1987;150(6):782–6.

47. Nasreen H, Kabir Z, Forsell Y, et al. Impact of maternal depressive symptoms and infant temperament on early infant growth and motor development: results from a population based study in Bangladesh. *J Affect Disord*. 2013;146(2):254–61.

48. Sharmin K, Sarwar N, Mumu S, et al. Postnatal depression and infant growth in an urban area of Bangladesh. *Midwifery*. 2019;74:57–67.

49. Wang H, Zhou H, Zhang Y, et al. Association of maternal depression with dietary intake, growth, and development of preterm infants: a cohort study in Beijing, China. *Front Med*. 2018;12(5):533–41.

50. Anato A, Baye K, Tafese Z, Stoecker B. Maternal depression is associated with child undernutrition: A cross-sectional study in Ethiopia. *Matern Child Nutr*. 2020;16(3):e12934.

51. Nabwera H, Moore S, Mwangome M, et al. The influence of maternal psychosocial circumstances and physical environment on the risk of severe wasting in rural Gambian infants: a mixed methods approach. *BMC Public Health*. 2018;18(1):109.

52. Amipara T, Baria H, Nayak S. A study on postpartum depression and its association with infant feeding practices and infant nutritional status among mothers attending the anganwadi centers of Valsad District, Gujarat, India. *Indian Journal of Community Medicine.* 2020;45(3):299–302.

53. Joshi M, Raut A. Maternal depression and its association with responsive feeding and nutritional status of infants: A cross-sectional study from a rural medical college in central India. *J Postgrad Med*. 2019;65(4):212–8.

54. Gordon S, Rotheram-Fuller E, Rezvan P, et al. Maternal depressed mood and child development over the first five years of life in South Africa. *J Affect Disord*. 2021 Nov 1;294:346–56.

55. Leroux X, Grose RG, Roof KA, et al. Mental health, empowerment, and violence against young women in lower-income countries: A review of reviews. *Aggression and Violent Behavior*. 2019 May;46:25–36.

56. Stansert Katzen L, le Roux K, Almirol E, et al. Community health worker home visiting in deeply rural South Africa: 12-month outcomes. *Glob Public Health*. 2020;1–14.

57. Tomlinson M, Rotheram-Borus M, Scheffler A, et al. Antenatal depressed mood and child cognitive and physical growth at 18-months in South Africa: a cluster randomised controlled trial of home visiting by community health workers. *Epidemiol Psychiatr Sci*. 2018;27(6):601–10.

58. Holm-Larsen C, Madsen F, Rogathi J, et al. Postpartum depression and child growth in Tanzania: a cohort study. *BJOG*. 2019;126(5):590–8.

59. Goldberg D, Bridges K, Duncan-Jones P, et al. Detecting anxiety and depression in general medical settings. *BMJ*. 1988;297(6653):897–9.

60. Hassan B, Werneck G, Hasselmann M. Maternal mental health and nutritional status of six-month-old infants. *Rev Saude Publica*. 2016;50:7.

61. Mezzavilla R de S, Barros Vianna GV de, Lindsay AC, et al. Intimate partner violence, breastfeeding, breastmilk substitutes and baby bottle use in the first year of life. *Ciência & Saúde Coletiva*. 2021;26(5):1955.

62. Rotheram-Borus M, Richter L, van Heerden A, et al. A cluster randomized controlled trial evaluating the efficacy of peer mentors to support South African women living with HIV and their infants. *PLoS One*. 2014;9(1):e84867.

63. Hamilton M. A rating scale for depression. *Journal of Neurology, Neurosurgery & Psychiatry*. 1960;23(1):56–62.

64. Mehnaz A, Shah N, Mala A, et al. Psychosocial determinants of nutritional neglect in a developing country. *Journal of the College of Physicians and Surgeons Pakistan.* 2014;24(5):335–9.

65. Rahman A, Malik A, Sikander S, et al. Cognitive behaviour therapy-based intervention by community health workers for mothers with depression and their infants in rural Pakistan: a cluster-randomised controlled trial. *Lancet*. 2008;372(9642):902–9.

66. Zigmond AS, Snaith RP. The Hospital Anxiety and Depression Scale. *Acta Psychiatr Scand.* 1983;67(6):361–70.

67. Ejaz MS, Sarwat A, Aisha T. Maternal psychiatric morbidity and childhood malnutrition. *Pakistan Journal of Medical Sciences*. 2012;28(5):874–8.

68. Derogatis LR, Lipman RS, Rickels K, Uhlenhuth EH, Covi L. The Hopkins Symptom Checklist (HSCL): A self-report symptom inventory. *Behavioral science*. 1974;19(1):1-5.

69. Hadley C, Tessema F, Muluneh A. Household food insecurity and caregiver distress: equal threats to child nutritional status? *Am J Hum Biol*. 2012;24(2):149–57.

70. Betancourt T, Jensen S, Barnhart D, et al. Promoting parent-child relationships and preventing violence via home-visiting: a pre-post cluster randomised trial among Rwandan families linked to social protection programmes. *BMC Public Health*. 2020;20(1):621.

71. Tomlinson M, Rotheram-Borus M, le Roux I, et al. Thirty-six-month outcomes of a generalist paraprofessional perinatal home visiting intervention in South Africa on maternal health and child health and development. *Prev Sci*. 2016;17(8):937–48.

72. Kaaya S, Garcia M, Li N, et al. Association of maternal depression and infant nutritional status among women living with HIV in Tanzania. *Matern Child Nutr*. 2016;12(3):603–13.

73. Bégin F, Frongillo E, Delisle H. Caregiver behaviors and resources influence child height-for-age in rural Chad. *J Nutr*. 1999;129(3):680–6.

74. Peter R, Kumar K. Mothers' caregiving resources and practices for children under 5 years in the slums of Hyderabad, India: a cross-sectional study. *WHO South East Asia J Public Health*. 2014;3(3):254–65.

75. Kroenke K, Spitzer RL, Williams JBW. The PHQ-9: Validity of a brief depression severity measure. *J Gen Intern Med.* 2001 Sep;16(9):606–13.

76. Motlhatlhedi K, Setlhare V, Ganiyu A, et al. Association between depression in carers and malnutrition in children aged 6 months to 5 years. *Afr J Prim Health Care Fam Med*. 2017;9(1):e1–6.

77. Woldetensay Y, Belachew T, Ghosh S, et al. The effect of maternal depressive symptoms on infant feeding practices in rural Ethiopia: community based birth cohort study. *Int Breastfeed J*. 2021;16(1):27.

78. Haithar S, Kuria M, Sheikh A, et al. Maternal depression and child severe acute malnutrition: a case-control study from Kenya. *BMC Pediatr*. 2018;18(1):289.

79. Adhikari R, Williamson R, Sparling T, et al. Parental depression and nutrition: findings from a cross-sectional household survey in Nepal. *Public Health Nutr*. 2020;23(16):2983–93.

80. Alarcón-Guevara S, Peñafiel-Sam J, Chang-Cabanillas S, et al. Maternal depressive symptoms are not associated with child anaemia: A cross-sectional population study in Peru, 2015. *Child Care Health Dev*. 2020;

81. Mohammedahmed A, Koko A, Arabi A, Ibrahim M. Maternal depression, a hidden predictor for severe acute malnutrition in children aged 6-59 months: a case-control study at Omdurman Paediatrics Teaching Hospital, Sudan. *Sudan J Paediatr*. 2020;20(2):111–21.

82. Neamah H, Sudfeld C, McCoy D, et al. Intimate partner violence, depression, and child growth and development. *Pediatrics*. 2018;142(1).

83. Dozio E, Le Roch K, Bizouerne C. Baby friendly spaces: an intervention for pregnant and lactating women and their infants in Cameroon. *Intervention*. 2020;18(1):78–84.

84. Pitt B. "Atypical" depression following childbirth. *Br J Psychiatry*. 1968;114(516):1325–35.

85. Avan B, Richter L, Ramchandani P, et al. Maternal postnatal depression and children's growth and behaviour during the early years of life: exploring the interaction between physical and mental health. *Arch Dis Child*. 2010;95(9):690–5.

86. Ickes S, Wu M, Mandel M, et al. Associations between social support, psychological well-being, decision making, empowerment, infant and young child feeding, and nutritional status in Ugandan children ages 0 to 24 months. *Matern Child Nutr*. 2018;14(1).

87. WHO. Schedules for Clinical Assessment in Neuropsychiatry: Manual. Version 2. Geneva: World Health Organization; 1994. 331 p. (SCAN 2.1). Available from: http://apps.who.int/iris/

88. Rahman A, Lovel H, Bunn J, et al. Mothers' mental health and infant growth: a case-control study from Rawalpindi, Pakistan. *Child Care Health Dev*. 2004;30(1):21–7.

89. First MB. User's guide for the structured clinical interview for DSM-IV axis II personality disorders: SCID-II. American Psychiatric Pub; 1997.

90. First MB, Spitzer RL, Gibbon M, et al. The structured clinical interview for DSM-III-R personality disorders (SCID-II). Part II: Multi-site test-retest reliability study. *Journal of Personality Disorders*. 1995;9(2):92–104.

91. Anoop S, Saravanan B, Joseph A, et al. Maternal depression and low maternal intelligence as risk factors for malnutrition in children: a community based case-control study from South India. *Arch Dis Child*. 2004;89(4):325–9.

92. Adewuya A, Ola B, Aloba O, et al. Impact of postnatal depression on infants' growth in Nigeria. *J Affect Disord*. 2008;108(1–2):191–3.

93. Fisher J, Tran T, Nguyen T, et al. Common mental disorders among women, social circumstances and toddler growth in rural Vietnam: a population-based prospective study. *Child Care Health Dev*. 2015;41(6):843–52.

94. Beusenberg M, Orley J, WHO. A User's Guide to the Self Reporting Questonnaire (SRQ). WHO; 1994. Available from: https://apps.who.int/iris/handle/10665/61113

95. Adams M, Khan N, Begum S, et al. Feeding difficulties in children with cerebral palsy: low-cost caregiver training in Dhaka, Bangladesh. *Child Care Health Dev*. 2012;38(6):878–88.

96. Basnet S, Frongillo E, Nguyen P, et al. Associations of maternal resources with care behaviours differ by resource and behaviour. *Matern Child Nutr*. 2020;16(3):e12977.

97. Cavalcante-Neto J, Paula C, Florêncio T, et al. Disability due to maternal common mental disorders (CMDs) as a risk factor for chronic childhood malnutrition: cross-sectional study. *Sao Paulo Med J*. 2016;134(3):228–33.

98. Nguyen P, Saha K, Ali D, et al. Maternal mental health is associated with child undernutrition and illness in Bangladesh, Vietnam and Ethiopia. *Public Health Nutr*. 2014;17(6):1318–27.

99. Santos D, Santos D, Silva Rde C, Hasselmann M, Barreto M. Maternal common mental disorders and malnutrition in children: a case-control study. *Soc Psychiatry Psychiatr Epidemiol*. 2011;46(7):543–8.

100. Prado E, Yakes Jimenez E, Vosti S, et al. Path analyses of risk factors for linear growth faltering in four prospective cohorts of young children in Ghana, Malawi and Burkina Faso. *BMJ Glob Health*. 2019;4(1):e001155.

101. Bennett IM, Schott W, Krutikova S, et al. Maternal mental health, and child growth and development, in four low-income and middle-income countries. *Journal of Epidemiology and Community Health*. 2015;70(2):168–73.

102. De Silva M, Harpham T. Maternal social capital and child nutritional status in four developing countries. *Health Place*. 2007;13(2):341–55.

103. Girma S, Fikadu T, Abdisa E. Maternal common mental disorder as predictors of stunting among children aged 6-59 months in Western Ethiopia: A case-control study. *Int J Pediatr*. 2019:4716482.

104. Harpham T, Huttly S, De Silva M, et al. Maternal mental health and child nutritional status in four developing countries. *J Epidemiol Community Health*. 2005;59(12):1060–4.

105. Medhin G, Hanlon C, Dewey M, et al. The effect of maternal common mental disorders on infant undernutrition in Butajira, Ethiopia: The P-MaMiE study. *BMC Psychiatry*. 2010;10.

106. Upadhyay A, Srivastava S. Effect of pregnancy intention, postnatal depressive symptoms and social support on early childhood stunting: findings from India. *BMC Pregnancy Childbirth*. 2016;16:107.

107. Colman S, Stewart R, MacArthur C, et al. Psychological distress in mothers of children admitted to a nutritional rehabilitation unit in Malawi: A comparison with other paediatric wards. *Matern Child Nutr*. 2015;11(4):915–25.

108. Stewart RC, Umar E, Kauye F, et al. Maternal common mental disorder and infant growth - a cross-sectional study from Malawi. *Maternal and Child Nutrition*. 2008;4(3):209–19.

109. Stewart R, Bunn J, Vokhiwa M, et al. A prospective study of psychological distress among mothers of children admitted to a nutritional rehabilitation unit in Malawi. *Child Care Health Dev*. 2011;37(1):55–63.

110. Abdullahi A, Farouk Z, Imam A. Common mental disorders in mothers of children attending out-patient malnutrition clinics in rural North-Western Nigeria: a cross-sectional study. *BMC Public Health*. 2021;21(1):185.

111. Brown N, Finch J, Obradović J, et al. Maternal care mediates the effects of nutrition and responsive stimulation interventions on young children's growth. *Child Care Health Dev*. 2017;43(4):577–87.

112. Favara M. Maternal group participation and child nutritional status in Peru. *Review of Development Economics*. 2018;22(2):459–83.

113. Thomas E, Kuo C, Cohen S, et al. Mental health predictors of breastfeeding initiation and continuation among HIV infected and uninfected women in a South African birth cohort study. *Prev Med*. 2017;102:100–11.

114. Zung WWK. A Self-Rating Depression Scale. *Arch Gen Psychiatry*. 1965;12(1):63.

115. Zhang JX, Guo SF, Li Y, et al. Factors influencing developmental delay among young children in poor rural China: a latent variable approach. *BMJ Open*. 2018;8(8).

116. Therrien A, Buffa G, Roome A, et al. Relationships between prenatal distress and infant body mass index in the first year of life in a lower-middle income country. *Int J Environ Res Public Health*. 2020;17(19).

117. Hajian-Tilaki K. Factors associated with the pattern of breastfeeding in the north of Iran. *Ann Hum Biol*. 2005;32(6):702–13.

118. Abidin RR. Parenting stress index third edition: Professional manual. Lutz: Psychological Assessment Resources Lutz: Inc. 1995;

119. Cohen S, Kamarck T, Mermelstein R. A global measure of perceived stress. *Journal of Health and Social Behavior*. 1983;24(4):385–96.

120. Vázquez-Vázquez A, Fewtrell MS, Chan-García H, et al. Does maternal grandmother's support improve maternal and child nutritional health outcomes? Evidence from Merida, Yucatan, Mexico. *Philos Trans R Soc Lond B Biol Sci*. 2021;376(1827):20200035.

121. Bliznashka L, Arsenault J, Becquey E, Ruel M, Olney D. Using structural equation modelling to understand the contributors to anaemia among young Burkinabe children. *Matern Child Nutr*. 2020;16(1):e12881.

122. DHS. Demographic and Health Surveys (DHS) Program. 2021. DHS Questionnaires. Available from: https://dhsprogram.com/methodology/survey-types/dhs-questionnaires.cfm

123. Essilfie G, Sebu J, Annim SK. Women’s empowerment and child health outcomes in Ghana. *African Development Review-Revue Africaine De Developpement*. 2020;32(2):200–15.

124. Chilinda Z, Wahlqvist M, Lee M,. Higher maternal autonomy is associated with reduced child stunting in Malawi. *Scientific Reports*. 2021;11(1):3882.

125. Kang Y, Kim J. Age-specific risk factors for child anaemia in Myanmar: Analysis from the Demographic and Health Survey 2015-2016. *Matern Child Nutr*. 2019;15(4):e12870.

126. Agu N, Emechebe N, Yusuf K, et al. Predictors of early childhood undernutrition in Nigeria: the role of maternal autonomy. *Public Health Nutr*. 2019;22(12):2279–89.

127. Ickes S, Hurst T, First V. Maternal literacy, facility birth, and education are positively associated with better infant and young child feeding practices and nutritional status among Ugandan children. *J Nutr*. 2015;145(11):2578–86.

128. Heaton T. Are improvements in child health due to increasing status of women in developing Nations? *Biodemography Soc Biol*. 2015;61(3):252–65.

129. Jones R, Haardörfer R, Ramakrishnan U, et al. Women’s empowerment and child nutrition: The role of intrinsic agency. *SSM Popul Health*. 2019;9:100475.

130. Na MZ, Jennings L, Talegawkar SA, et al. Association between women’s empowerment and infant and child feeding practices in sub-Saharan Africa: an analysis of Demographic and Health Surveys. *Public Health Nutrition*. 2015;18(17):3155–65.

131. MICS. Lao PDR multiple indicator cluster survey 2006: final report. Vientiane: Department of Statistics, Lao PDR; 2008.

132. Kamiya Y. Socioeconomic determinants of nutritional status of children in Lao PDR: effects of household and community factors. *J Health Popul Nutr*. 2011;29(4):339–48.

133. IIPS, ORC MACRO. National Family Health Survey (NFHS-II) 1998-99. Mumbai, India. Calverton, Maryland, USA: International Institute for Population Sciences; ORC MACRO; 2000.

134. Shroff M, Griffiths P, Adair L, et al. Maternal autonomy is inversely related to child stunting in Andhra Pradesh, India. *Matern Child Nutr*. 2009;5(1):64–74.

135. IIPS, ORC MACRO. National Family Health Survey (NFHS-III), 2005-06. Mumbai, India. Calverton, Maryland, USA: International Institute for Population Sciences; ORC MACRO; 2006. Available from: https://dhsprogram.com/pubs/pdf/frind3/frind3-vol1andvol2.pdf

136. Sinha A, McRoy RG, Berkman B, et al. Drivers of change: Examining the effects of gender equality on child nutrition. *Children & Youth Services Review*. 2017;76:203–12.

137. Waszak C, Severy LJ, Kafafi L, Badawi I. Fertility Behavior and Psychological Stress: the Mediating Influence of Gender Norm Beliefs Among Egyptian Women. Psychology of Women Quarterly. 2001 Sep;25(3):197–208.

138. Kumar N, Nguyen PH, Harris J, Harvey D, Rawat R, Ruel MT. What it takes: evidence from a nutrition- and gender-sensitive agriculture intervention in rural Zambia. *Journal of Development Effectiveness*. 2018;10(3):341–72.

139. Straus MA. Measuring Intrafamily Conflict and Violence: The Conflict Tactics (CT) Scales. *Journal of Marriage and Family*. 1979;41(1):75–88.

140. Chowdhury M, Khan H, Mondal M, et al. Socio-demographic risk factors for severe malnutrition in children aged under five among various birth cohorts in Bangladesh. *Journal of Biosocial Sciences*. 2020;1–16.

141. Ziaei S, Naved R, Ekström E. Women's exposure to intimate partner violence and child malnutrition: findings from demographic and health surveys in Bangladesh. *Matern Child Nutr*. 2014;10(3):347–59.

142. Rahman M, Poudel K, Yasuoka J, et al. Maternal exposure to intimate partner violence and the risk of undernutrition among children younger than 5 years in Bangladesh. *Am J Public Health*. 2012;102(7):1336–45.

143. Tsedal D, Yitayal M, Abebe Z, et al. Effect of intimate partner violence of women on minimum acceptable diet of children aged 6-23 months in Ethiopia: evidence from 2016 Ethiopian Demographic and Health Survey. *BMC Nutr*. 2020;6:28.

144. Sobkoviak R, Yount K, Halim N. Domestic violence and child nutrition in Liberia. *Soc Sci Med*. 2012;74(2):103–11.

145. Mukong A, Burns J. Parental bargaining and rural-urban child health differential in Tanzania. *Development Southern Africa*. 2021; 38(6):938-953.

146. Chai J, Fink G, Kaaya S, et al. Association between intimate partner violence and poor child growth: results from 42 demographic and health surveys. *Bull World Health Organ*. 2016;94(5):331–9.

147. Rico E, Fenn B, Abramsky T, et al. Associations between maternal experiences of intimate partner violence and child nutrition and mortality: Findings from Demographic and Health Surveys in Egypt, Honduras, Kenya, Malawi and Rwanda. *J Epidemiol Community Health*. 2011;65(4):360–7.

148. Jewkes R, Levin J, Penn-Kekana L. Risk factors for domestic violence: findings from a South African cross-sectional study. *Social Science & Medicine*. 2002;55(9):1603–17.

149. Batool Z, Zafar MI, Maann AA, Ali T. Socio-cultural factors affecting anemia and their effects on mother, and child health in rural areas of district Faisalabad, Punjab, Pakistan. *Pakistan Journal of Agricultural Sciences*. 2010;47(1):59–65.

150. Asling-Monemi K, Naved RT, Persson LA. Violence against women and the risk of fetal and early childhood growth impairment: a cohort study in rural Bangladesh. *Archives of Disease in Childhood*. 2009;94(10):775–9.

151. Straus MA, Douglas EM. A short form of the Revised Conflict Tactics Scales, and typologies for severity and mutuality. *Violence Vict.* 2004;19(5):507–20.

152. Naved RT, Azim S, Bhuiya A, et al. Physical violence by husbands: Magnitude, disclosure and help-seeking behavior of women in Bangladesh. *Social Science & Medicine.* 2006;62(12):2917–29.

153. Deshpande NA, Lewis-O'Connor A. Screening for intimate partner violence during pregnancy. *Rev Obstet Gynecol.* 2013;6(3–4):141–8.

154. Das S, Chanani S, Shah More N, et al. Determinants of stunting among children under 2 years in urban informal settlements in Mumbai, India: evidence from a household census. *J Health Popul Nutr.* 2020;39(1):10.

155. Ackerson L, Subramanian S. Domestic violence and chronic malnutrition among women and children in India. *Am J Epidemiol*ogy. 2008;167(10):1188–96.

156. Adhikari T, Vir SC, Yadav RJ, et al. Undernutrition in children under-two years (u2) in India: An analysis of determinants. *Statistics and Applications*. 2015;13(1–2):25–36.

157. Imai KS, Annim SK, Kulkarni VS, et al. Women's empowerment and prevalence of stunted and underweight children in rural India. *World Development*. 2014;62:88–105.

158. IIPS, ICF. National Family Health Survey (NFHS-IV), 2015-16. Mumbai, India: International Institute for Population Sciences, ICF International; 2017.

159. Metheny N, Stephenson R. Is Intimate Partner Violence a Barrier to Breastfeeding? An Analysis of the 2015 Indian National Family Health Survey. *Journal of Family Violence.* 2020;35(1):53–64.

160. Mondal D, Paul P. Association between intimate partner violence and child nutrition in India: Findings from recent National Family Health Survey. *Children and Youth Services Review*. 2020;119.

161. Sethuraman K, Lansdown R, Sullivan K. Women's empowerment and domestic violence: the role of sociocultural determinants in maternal and child undernutrition in tribal and rural communities in South India. *Food Nutr Bull*. 2006;27(2):128–43.

162. OECD. OECD Guidelines on Measuring Subjective Well-being. 2013. Available from: https://www.oecd-ilibrary.org/content/publication/9789264191655-en

163. Garcia-Moreno C, Jansen HAFM, Ellsberg M, et al. Prevalence of intimate partner violence: findings from the WHO multi-country study on women's health and domestic violence. *Lancet*. 2006; 368(9543):1260–9.

164. Briaux J, Martin-Prevel Y, Carles S, et al. Evaluation of an unconditional cash transfer program targeting children's first-1,000–days linear growth in rural Togo: A cluster-randomized controlled trial. *PLOS Medicine*. 2020;17(11):e1003388.

165. Agadjanian V, Jansen N. Historical Legacies, Social Capital, and Women's Decision-Making Power: Religion and Child Nutrition in Mozambique. J Relig Health. 2018;57(4):1458–72.

166. Harpham T, Grant E, Thomas E. Measuring social capital within health surveys: Key issues. *Health Policy and Planning*. 2002;17(1):106-111.

167. Kang Y, Kim J, Seo E. Association between maternal social capital and infant complementary feeding practices in rural Ethiopia. *Matern Child Nutr*. 2018;14(1): e12484.

168. Domingo DGC, Barba CVC, Talavera MTM, et al. Association of maternal social capital with nutritional status of 6 to 24-month-old children living in urban and rural areas in Laguna, Philippines. *Malaysian Journal of Nutrition*. 2017;23(1):53–63.

169. Grootaert C, Narayan D, Jones VN, Woolcock M. Measuring Social Capital: An Integrated Questionnaire. In Washington, DC: World Bank; 2004. (World Bank Working Paper;No. 18). Available from: https://openknowledge.worldbank.org/handle/10986/15033

170. Win T, Tun Sein T, Ikeda W, et al. Does father's social capital matter to child undernutrition in Myanmar? *Asia Pac J Public Health*. 2020;1010539520951717.

171. Kulkarni S, Frongillo E, Cunningham K, et al. Women's bargaining power and child feeding in Nepal: Linkages through nutrition information. *Matern Child Nutr*. 2020;16(1):e12883.

172. Lau L, Dodd W, Qu H, et al. Exploring trust in religious leaders and institutions as a mechanism for improving retention in child malnutrition interventions in the Philippines: a retrospective cohort study. *BMJ Open*. 2020;10(9):e036091.

173. Moestue H, Huttly S, Sarella L, et al. "The bigger the better": Mothers' social networks and child nutrition in Andhra Pradesh. *Public Health Nutrition*. 2007;10(11):1274–82.

174. Adelman S. Keep your friends close: The effect of local social networks on child human capital outcomes. *Journal of Development Economics*. 2013;103:284–98.

175. Hadley C. The costs and benefits of kin: Kin networks and children's health among the Pimbwe of Tanzania. *Hum Nat*. 2004;15(4):377–95.

176. Wutich A, McCarty C. Social networks and infant feeding in Oaxaca, Mexico. *Matern Child Nutr*. 2008;4(2):121–35.

177. Reyes H, Pérez-Cuevas R, Sandoval A, et al. The family as a determinant of stunting in children living in conditions of extreme poverty: A case-control study. *BMC Public Health.* 2004;4:57.

178. Nguyen PH, Frongillo EA, Kim SS, et al. Information diffusion and social norms are associated with infant and young child feeding practices in Bangladesh. *J Nutr*. 2019;149(11):2034–45.

179. Chor D, Griep RH, Lopes CS, et al. Social network and social support measures from the Pró-Saúde Study: pre-tests and pilot study. *Cad Saude Publica.* 2001;17(4):887–96.

180. Santoso MV, Kerr RB, Hoddinott J, et al. Role of women’s empowerment in child nutrition outcomes: A systematic review. *Advances in Nutrition*. 2019;10(6):1138–51.

181. Santoso MV. Evaluating the impact of a participatory nutrition-sensitive agriculture intervention on women’s empowerment and child’s diet in Singida, Tanzania. [Dissertation]. Ithaca, NY: Cornell University; 2019.

182. Tuan N, Nguyen P, Hajeebhoy N, et al. Gaps between breastfeeding awareness and practices in Vietnamese mothers result from inadequate support in health facilities and social norms. *J Nutr*. 2014;144(11):1811–7.

183. Barman D, Vadrevu L. How is perceived community cohesion and membership in community groups associated with children’s dietary adequacy in disadvantaged communities? A case of the Indian Sundarbans. *BMC Health Services Research*. 2016;16, 73-82

184. Paina L, Vadrevu L, Hanifi SMMA, et al. What is the role of community capabilities for maternal health? An exploration of community capabilities as determinants to institutional deliveries in Bangladesh, India, and Uganda. *BMC Health Services Research*. 2016;16(7):621.

185. Broadhead W, Gehlbach S, De Gruy F, et al. The Duke-UNC Functional Social Support Questionnaire: Measurement of social support in family medicine patients. *Medical Care*. 1988;26(7):709–23.

186. Ziaei S, Contreras M, Zelaya Blandón E, et al. Women’s autonomy and social support and their associations with infant and young child feeding and nutritional status: community-based survey in rural Nicaragua. *Public Health Nutr*. 2015;18(11):1979–90.

187. Petraro P, Duggan C, Msamanga G,. Predictors of breastfeeding cessation among HIV-infected women in Dar es Salaam, Tanzania. *Matern Child Nutr*. 2011;7(3):273–83.

188. Pradanie R, Rachmawati PD, Cahyani MD. Factors Associated with mothers' behaviors in selecting complementary feeding in Surabaya, Indonesia. *Nurse Media Journal of Nursing*. 2020;10(3):306–16.

189. Kulkarni S, Frongillo E, Cunningham K, et al. Gendered intrahousehold bargaining power is associated with child nutritional status in Nepal. *J Nutr*. 2021;151(4):1018–24.

190. Alaofè H, Zhu M, Burney J, et al. Association Between women's empowerment and maternal and child nutrition in Kalalé District of Northern Benin. *Food Nutr Bull*. 2017;38(3):302–18.

191. Webster J, Linnane JWJ, Dibley LM, et al. Measuring social support in pregnancy: Can it be simple and meaningful? *Birth*. 2000;27(2):97–101.

192. Sherbourne CD, Stewart AL. The MOS Social Support Survey. *Social Science & Medicine.* 1991 Jan;32(6):705–14.

193. Zimet G, Dahlem N, Zimet S, Farley G. The Multidimensional Scale of Perceived Social Support. *Journal of Personality Assessment*. 1988;52(1):30–41.

194. Usman MA, Kornher L, Sakketa TG. Do non-maternal adult female household members influence child nutrition? Empirical evidence from Ethiopia. *Maternal and Child Nutrition* 2021;17(S1): e13123.

195. Kocalevent RD, Berg L, Beutel ME, et al. Social support in the general population: standardization of the Oslo social support scale (OSSS-3). *BMC Psychology*. 2018;6(1):31.

196. Tran B. Relationship between paternal involvement and child malnutrition in a rural area of Vietnam. *Food Nutr Bull.* 2008;29(1):59–66.

197. Titiloye M, Brieger W. Infant weaning methods: an intergenerational view from Igbo-Ora Nigeria. *Int Q Community Health Educ*. 2008 2009;29(4):323–34.

198. Cohen S, Mermelstein R, Kamarck T, et al. Measuring the functional components of social support. In: Social support: theory, research, and applications. Sarason, I.G. & Sarason, B.R. The Hague, Netherlands: Martinus Niijhoff; 1985.

199. Adams AM, Madhavan S, Simon D. Women's social networks and child survival in Mali. *Social Science & Medicine*. 2002;54(2):165–78.

200. de BL Carvalhaes M, D'Aquino Benício M, Barros A. Social support and infant malnutrition: a case-control study in an urban area of Southeastern Brazil. *Br J Nutr*. 2005;94(3):383–9.

201. Mukuria A, Martin S, Egondi T, et al. Role of social support in improving infant feeding practices in Western Kenya: A quasi-experimental study. *Glob Health Sci Pract*. 2016;4(1):55–72.

202. Leslie J, Powell D, Jackson J, et al. The relationship between maternal work patterns and infant feeding practices in Jamaican female headed households. *Ecology of Food and Nutrition*. 2001;40(3):215–52.

203. Hein A, Hong S, Puckpinyo A, et al. Dietary diversity, social support and stunting among children aged 6-59 months in an internally displaced persons camp in Kayin State, Myanmar. *Clin Nutr Res*. 2019;8(4):307–17.

204. Barrera M, Sandler IN, Ramsay TB. Preliminary development of a scale of social support: Studies on college students. *American Journal of Community Psychology*. 1981;9:435–47.

205. Leroux I, Rotheram-Borus M, Stein J, et al. The impact of paraprofessional home visitors on infants' growth and health at 18 months. *Vulnerable Child Youth Stud*. 2014;9(4):291–304.

206. Muelbert M, Giugliani E. Factors associated with the maintenance of breastfeeding for 6, 12, and 24 months in adolescent mothers. *BMC Public Health*. 2018;18(1):675.

207. Nakahara S, Poudel KC, Lopchan M, et al. Availability of childcare support and nutritional status of children of non-working and working mothers in Urban Nepal. *American Journal of Human Biology*. 2006;18(2):169–81.

208. Pierre-Louis J, Sanjur D, Nesheim M, et al. Maternal income-generating activities, child care, and child nutrition in Mali. *Food Nutr Bull*. 2007;28(1):67–75.

209. Nordang S, Shoo T, Holmboe-Ottesen G, et al. Women's work in farming, child feeding practices and nutritional status among under-five children in rural Rukwa, Tanzania. *Br J Nutr*. 2015;114(10):1594–603.

210. Alkire S, Meinzen-Dick R, Peterman A, et al. The Women's Empowerment in Agriculture Index. Oxford: Oxford Poverty & Human Development Initiative; 2013. Available from: http://www.ophi.org.uk/wp-content/uploads/ophi-wp-58.pdf?cda6c1

211. Seymour G, Masuda YJ, Williams J, et al. Household and child nutrition outcomes among the time and income poor in rural Bangladesh. *Global Food Security-Agriculture Policy Economics and Environment*. 2019;20:82–92.

212. Cunningham K, Ploubidis GB, Menon P, et al. Women's empowerment in agriculture and child nutritional status in rural Nepal. *Public Health Nutrition*. 2015;18(17):3134–45.

213. Malapit HJL, Kadiyala S, Quisumbing AR, et al. Women's empowerment mitigates the negative effects of low production diversity on maternal and child nutrition in Nepal. *Journal of Development Studies*. 2015;51(8):1097–123.

214. Komatsu H, Malapit HJL, Theis S. Does women's time in domestic work and agriculture affect women's and children's dietary diversity? Evidence from Bangladesh, Nepal, Cambodia, Ghana, and Mozambique. *Food Policy*. 2018;79:256–70.

215. van den Bold M, Bliznashka L, Ramani G, et al. Nutrition-sensitive agriculture programme impacts on time use and associations with nutrition outcomes. *Matern Child Nutr*. 2021;17(2):e13104.

216. Lakshman RR, Landsbaugh JR, Schiff A, et al. Development of a questionnaire to assess maternal attitudes towards infant growth and milk feeding practices. *International Journal of Behavioral Nutrition and Physical Activity*. 2011;8(1):35.

217. Bandura A. Health promotion from the perspective of social cognitive theory. *Psychology & Health*. 1998;13(4):623–49.

218. Husain N, Zulqernain F, Carter LA, et al. Treatment of maternal depression in urban slums of Karachi, Pakistan: A randomized controlled trial (RCT) of an integrated maternal psychological and early child development intervention. *Asian Journal of Psychiatry*. 2017;29:63–70.

219. Mahmud WMRW, Awang A, Herman I, et al. Analysis of the psychometric properties of the malay version of Beck Depression Inventory II (BDI-II) among postpartum women in Kedah, North West of Peninsular Malaysia. *Malays J Med Sci*. 2004;11(2):19–25.

220. Rochat TJ, Tomlinson M, Newell ML, et al. Detection of antenatal depression in rural HIV-affected populations with short and ultrashort versions of the Edinburgh Postnatal Depression Scale (EPDS). *Archives of Women's Mental Health*. 2013;16(5):401–10.

221. Gausia K, Fisher C, Algin S, et al. Validation of the Bangla version of the Edinburgh Postnatal Depression Scale for a Bangladeshi sample. *Journal of Reproductive and Infant Psychology*. 2007;25(4):308–15.

222. Chibanda D, Mangezi W, Tshimanga M, et al. Validation of the Edinburgh Postnatal Depression Scale among women in a high HIV prevalence area in urban Zimbabwe. *Arch Womens Ment Health*. 2010;13(3):201–6.

223. Raina P. The health and well-being of caregivers of children with cerebral palsy. *Pediatrics*. 2005;115(6):e626–36.

224. Rahman A, Iqbal Z, Waheed W, et al. Translation and cultural adaptation of health questionnaires. *J Pak Med Assoc*. 2003;53(4):142–7.

225. National Population Commission, ICF International. Nigeria Demographic and Health Survey 2013. Abuja, Nigeria: NPC/Nigeria and ICF International; 2014. Available from: http://dhsprogram.com/pubs/pdf/FR293/FR293.pdf

226. Ghana Statistical Service (GSS), Ghana Health Service (GHS), ICF International. Ghana Demographic and Health Survey 2014. Rockville, Maryland, USA: GSS, GHS, and ICF International; 2015. Available from: https://dhsprogram.com/pubs/pdf/fr307/fr307.pdf

227. Uganda Bureau of Statistics (UBOS), Macro International Inc, ICF International Inc. Uganda DHS Questionnaire. Rockville, Maryland, USA: ICF.

228. Kishor S, Subaiya L. Understanding women's empowerment: a comparative analysis of Demographic and Health Surveys (DHS) data. Calverton, Maryland, USA: Macro International; 2008. (DHS Comparative Reports No. 20). Available from: http://dhsprogram.com/pubs/pdf/CR20/CR20.pdf

229. MHoS, ICF. Myanmar Demographic and Health Survey 2015-16. Nay Pyi Taw, Myanmar: Ministry of Health and Sports - MoHS/Myanmar and ICF; 2017. Available from: http://dhsprogram.com/pubs/pdf/FR324/FR324.pdf

230. Nanda G. Compendium of Gender Scales. Washington, DC: FHI 360/C-Change; 2011.

231. Hasselmann MH, Reichenheim ME. Cross-cultural adaptation of the Portuguese version of the Conflict Tactics Scales Form R (CTS-1) used to assess marital violence: semantic and measurement equivalence. *Cad Saúde Pública*. 2003;19(4).

232. MoHCDGEC, MoH, NBS, OCGS, ICF. Tanzania Demographic and Health Survey and Malaria Indicator Survey 2015-2016. Dar es Salaam, Tanzania: Ministry of Health Community Development, Gender, Elderly and Children (MoHCDGEC), Ministry of Health, National Bureau of Statistics (NBS), Office of Chief Government Statistician (OCGS), and ICF; 2016. Available from: http://dhsprogram.com/pubs/pdf/FR321/FR321.pdf

233. National Bureau of Statistics, ICF Macro. Tanzania Demographic and Health Survey 2010. Dar es Salaam, Tanzania: NBS/Tanzania and ICF Macro; 2011. Available from: http://dhsprogram.com/pubs/pdf/FR243/FR243.pdf

234. NISR, MOH, ICF. Rwanda Demographic and Health Survey 2019-20. Kigali, Rwanda and Rockville, Maryland, USA: National Institute of Statistics of Rwanda (NISR), Ministry of Health (MOH), ICF; 2021. Available from: https://www.dhsprogram.com/pubs/pdf/FR370/FR370.pdf

235. NIPORT, Mitra and Associates, Macro International. Bangladesh Demographic and Health Survey 2007 (BDHS 2007). Dhaka, Bangladesh and Calverton, MD: National Institute of Population Research and Training (NIPORT), Mitra and Associates, & Macro International; 2007.

236. Alvarado BE, Zunzunegui MV, Delisle H. Validation of food security and social support scales in an Afro-Colombian community: Application on a prevalence study of nutritional status in children aged 6 to 18 months. *Cad Saude Publica*. 2005;21(3):724–36.

237. Ickes SB, Heymsfield GA, Wright TW, et al. "Generally the young mom suffers much:" Socio-cultural influences of maternal capabilities and nutrition care in Uganda. Maternal & Child Nutrition. 2017;13(3):e12365.

238. Mukuria A. Exclusive breastfeeding and the role of social support and social networks in a low-income urban community in Nairobi, Kenya [Dissertation]. Baltimore, MD: Johns Hopkins University, Bloomberg School of Public Health; 1999.

239. USAID Infant & Young Child Nutrition Project. Engaging Grandmothers and Men in Infant and Young Child Feeding and Maternal Nutrition: Report of a Formative Assessment in Eastern and Western Kenya. Washington, DC: USAID; 2011.

240. Galab S, Reddy MG, Antony P, et al. Young Lives Preliminary Country Report: Andhra Pradesh, India. 2003.

241. Wandel M, Holmboe-Ottesen G. Maternal work, child feeding, and nutrition in rural Tanzania. *Food Nutr Bull*. 1992 Mar 1;14(1):1–6.

242. Bardasi E, Wodon Q. Measuring Time Poverty and Analyzing its Determinants: Concepts and Application to Guinea. In: Gender, Time Use, and Poverty in sub-Saharan Africa. 2006. p. 75–95. (World Bank Working Paper). Available from: https://mpra.ub.uni-muenchen.de/11082/
